# Direct Hydrodecarboxylation of Aliphatic Carboxylic Acids: Metal- and Light-Free

**DOI:** 10.1021/acs.orglett.1c04079

**Published:** 2022-01-07

**Authors:** Euan B. McLean, David T. Mooney, David J. Burns, Ai-Lan Lee

**Affiliations:** † Institute of Chemical Sciences, Heriot-Watt University, Edinburgh EH14 4AS, Scotland, United Kingdom; ‡ Syngenta, Jealott’s Hill International Research Centre, Bracknell, Berkshire RG42 6EY, United Kingdom

## Abstract

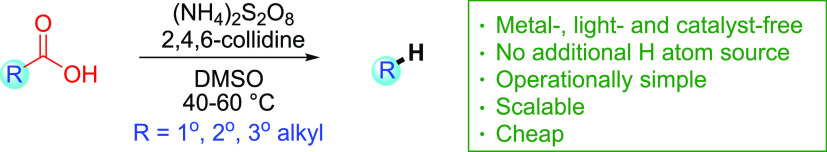

A mild and inexpensive method for direct hydrodecarboxylation of aliphatic carboxylic acids has been developed. The reaction does not require metals, light, or catalysts, rendering the protocol operationally simple, easy to scale, and more sustainable. Crucially, no additional H atom source is required in most cases, while a broad substrate scope and functional group tolerance are observed.

The carboxylic acid moiety, and its derivatives, is one of the most abundant and synthetically versatile functional groups that is present in many naturally occurring compounds.^1^ The ability of carboxylic acids to promote a range of different chemical transformations, particularly C–C bond-forming reactions, makes these compounds highly valuable starting materials for organic synthesis.^2^ However, the carboxylic acid functionality is often unwanted in later synthetic intermediates, so methods for removing the carboxylic acid functionality via hydrodecarboxylation are highly sought after.

The most famous of these is the Barton decarboxylation,^3^ but the reaction suffers from several notable drawbacks. The reaction requires two steps (via activated ester), harsh reaction conditions, and the use of notoriously noxious H atom donors [(^*n*^Bu)_3_SnH]. In recent years, progress has been made to render hydrodecarboxylations of aliphatic carboxylic acids more palatable to modern synthetic chemists. For example, less toxic hydrogen atom donors have been used (e.g., Scheme 1A), such as silanes,^4^ thiols,^5^ and chloroform;^6^ however, drawbacks such as harsh reaction conditions, the use of toxic or unsustainable transition metals, complex reaction mixtures, two-step protocols, and poor atom economy remain.^7^ Meanwhile, milder reactions were also developed by harnessing visible light through the use of photocatalysis^8^ and electron–donor–acceptor complexes,^9^ although these still mainly proceed via activated esters. A notable and seminal example of direct decarboxylation is that of Nicewicz (Scheme 1B);^8b^ however, it requires a photocatalytic setup with associated scalability issues,^10^ use of a glovebox, extended reaction times, and odorous thiols (formed *in situ*) as the H atom source. A significant advancement in the field would therefore be a direct hydrodecarboxylation that addresses all of the limitations of the original Barton decarboxylation, while also being operationally simple, scalable, and more sustainable. We herein describe the first metal-, catalyst-, and light-free direct hydrodecarboxylation procedure for aliphatic carboxylic acids that not only fits all of the criteria mentioned above but also crucially does not require an additional H atom source (Scheme 1C).^11^

**Scheme 1:**
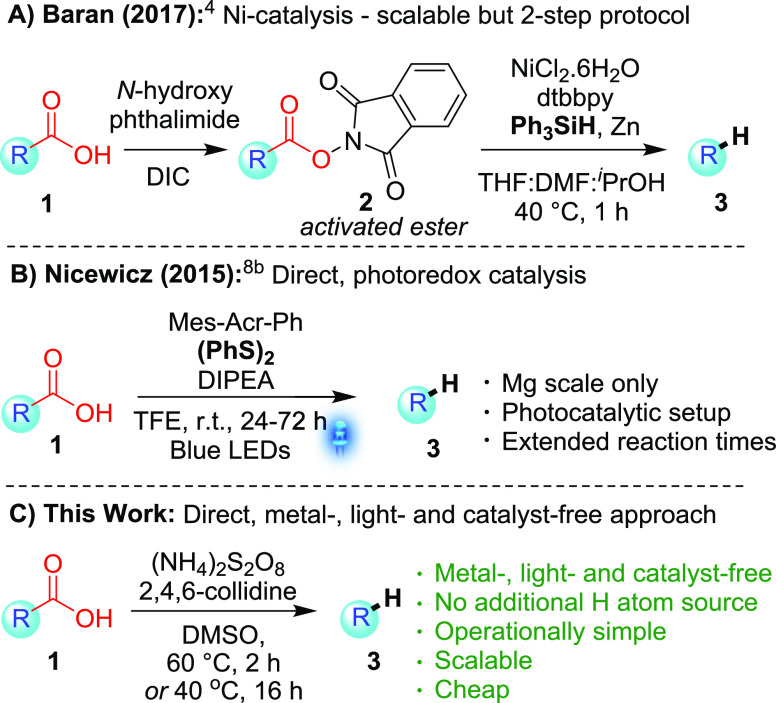
Notable Developments in Hydrodecarboxylations

The inspiration for our work was our recent discovery that Minisci-type reactions can proceed under mild conditions without any metal, photocatalyst, or light.^12^ The use of DMSO as solvent was thought to allow for the breakdown of S_2_O_8_^2–^ to the active SO_4_^–**•**^ under mild conditions, without the need for the previously used metal mediation or photolysis.^13^ We were also inspired by the original Kochi hydrodecarboxylation [Ag(II), S_2_O_8_^2–^, and heat], although low yields, poor selectivity, a limited substrate scope, and expensive/unsustainable use of silver have so far limited any widespread utility in synthetic applications.^14^

We commenced our investigations with substrate **1a**, using conditions based on our previously reported Minisci-type alkylation,^12a^ but in the absence of the heterocycle radical acceptor. Disappointingly, these initial conditions failed to produce the desired **3a** (Table 1, entry 1). To our delight, the inclusion of 2,4,6-collidine allowed us to observe **3a** in a moderate yield of 22% (entry 2). The desired reactivity could be obtained from other basic additives (see the Supporting Information for the full study), indicating that 2,4,6-collidine is acting as a base to promote the reaction (entries 3–5). Changes to the stoichiometry of 2,4,6-collidine did not have a positive impact (entries 6 and 7). Increasing the temperature to 60 °C proved to be beneficial, increasing the yield of **3a** to 57% [entry 8 (see the Supporting Information for rates at different temperatures)]. The water content had no appreciable impact (entries 8–10). Pleasingly, the yield increased to 68% with 3 equiv of (NH_4_)_2_S_2_O_8_ (entry 11). Other persulfates also promote the transformation (entries 12 and 13), with (NH_4_)_2_S_2_O_8_ giving the best performance presumably due to increased solubility. Control reactions show that the reaction performs equally well in the dark (entry 14) and that both 2,4,6-collidine and (NH_4_)_2_S_2_O_8_ are crucial (entries 15 and 16, respectively). Running the reaction under air also forms desired **3a**, albeit with a decrease in yield from 68% to 46% (entry 17). It should be noted that *d*_6_-DMSO was used solely for ease of ^1^H NMR analysis; the reaction performs just as well in nondeuterated DMSO.

**Table 1:**
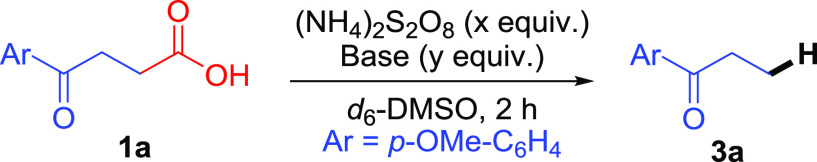
Selected Optimization and Control Studies

entry	base^a^	notes	*T* (°C)	*x*	*y*	**1a**^d^ (%)	**3a**^d^ (%)
1^b^,^c^	–		40	2	–	nd	0
2^b^,^c^	collidine		40	2	3	<5	22
3^b^,^c^	Na_2_CO_3_		40	2	3	5	11
4	lutidine		60	2	3	27	22
5	pyridine		60	2	3	<5	5
6^b^,^c^	collidine		40	2	2	nd	13
7^b^,^c^	collidine		40	2	5	nd	24
8^e^	collidine		60	2	3	25	57
9	collidine	anhydrous	60	2	3	23	56
10	collidine		60	2	3	30	60
11	collidine		60	3	3	16	68
12	collidine	Na_2_S_2_O_8_	60	3	3	23	27
13	collidine	K_2_S_2_O_8_	60	3	3	31	21
14	collidine	in the dark	60	3	3	25	67
15	–	no base	60	3	–	95	<5
16	collidine	no S_2_O_8_^2–^	60	–	3	100	<5
17	collidine	under air	60	3	3	25	46

a Collidine = 2,4,6-collidine; lutidine = 2,6-lutidine.

b DMSO/H_2_O (600:1) as the solvent.

c For 16 h.

d Yields determined by ^1^H NMR analysis using dimethylsulfone or 1,3,5-trimethoxybenzene as an internal standard.

e *d*_6_-DMSO/H_2_O (600:1).

With the optimized reaction conditions in hand, we began to investigate the substrate scope (Scheme 2). Primary carboxylic acids are tolerated, with model substrate **1a** forming **3a** in 68% yield. 1,3-Ketoacids were shown to be excellent substrates (73% **3b**). In contrast, long chain fatty acid **1c** reacted more sluggishly and provided **3c** in a modest 35% yield. These results indicate that for primary carboxylic acids, having a polar withdrawing group (e.g., carbonyl) in the proximity of the carboxylic acid helps to promote radical formation. Substrate **1d** corroborates our theory, as moving the carbonyl one carbon away (vs **1a**) substantially decreases the reactivity and yield (68% **3a** vs 11% **3d**).^15^ Pleasingly, amino acid derivatives were compatible substrates, with protected glutamic acid **1e** providing the desired **3e** in 59% yield.

**Scheme 2:**
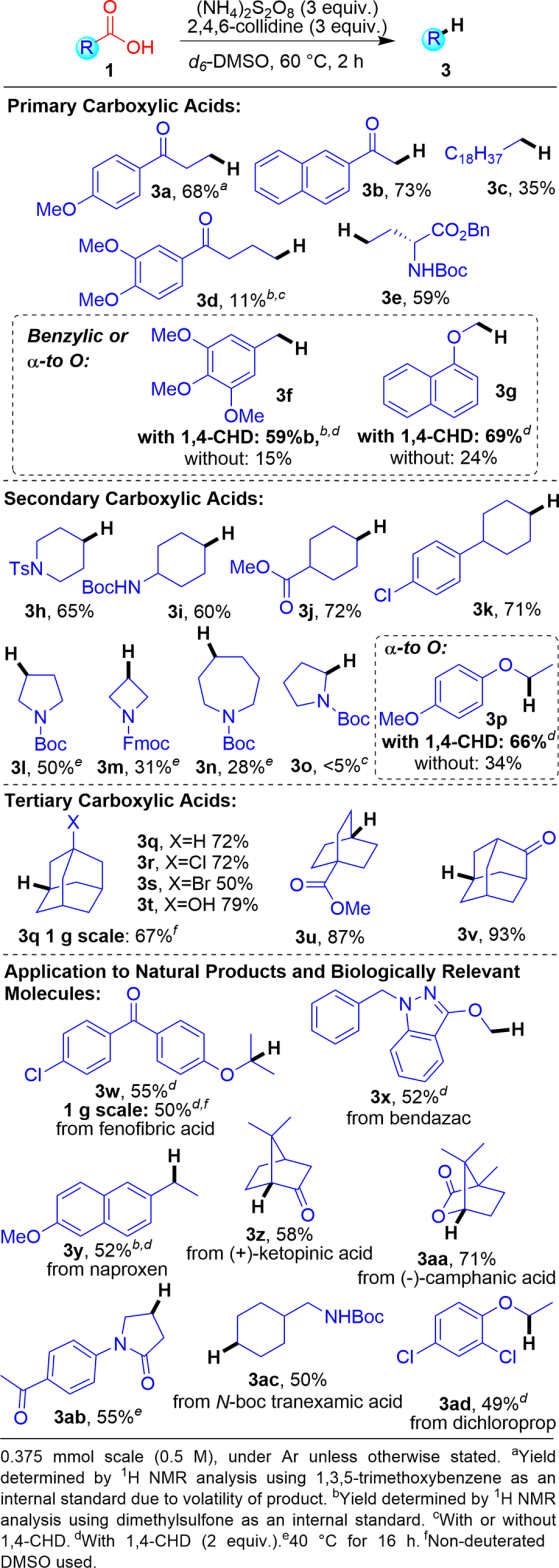
Substrate Scope Studies

Secondary carboxylic acids were good substrates with **3h** obtained in good yield (65%). Protected amines, esters, and Cl groups were all shown to be compatible, furnishing products **3i–3k**, respectively, in good yields (60–72%). Contracting the ring size of the substrate initially proved to be problematic, with substrate **1l** performing poorly under the standard conditions due to high reactivity. Nevertheless, these problems could be mitigated by decreasing the temperature to 40 °C to produce **3l** in 50% yield. Cyclic carboxylic acids with four- and seven-membered rings (**1m** and **1n**, respectively) required similar treatment to access desired products **3m** and **3n**, albeit in reduced yields (31% and 28%, respectively).^16^ While the reaction exhibits a high degree of functional group tolerance, substrates in which the acid functionality is α to nitrogen, such as in l-proline (**1o**), gave a complex mixture of products with no desired **3o** observed.

Tertiary carboxylic acids proved to be excellent substrates. Adamantane (**3q**) could be obtained in a good yield of 72%. Halogenated substrates (**1r** and **1s**) and free hydroxyl-containing **1t** were all tolerated, providing **3r–3t** in moderate to good yields (50–79%). The performance of bromine-containing substrate **1s** was particularly pleasing as this functional group can be susceptible to transformations of a radical nature. Strained ring system **1u** was also compatible, furnishing **3u** in 87% yield. Cyclic ketone **1v** performed well, producing **3v** in an excellent 93% yield.

The reaction is very readily scalable under our operationally simple and inexpensive conditions, as exemplified by the gram scale reaction on **1q** to produce **3q** in 67% yield.

Studies using our initial standard conditions demonstrated a wide substrate scope encompassing primary, secondary, and tertiary carboxylic acids, and excellent functional group compatibility. Nevertheless, we identified certain classes of carboxylic acids **1** that were too reactive for our initial standard conditions. In particular, benzylic or α to O substrates (e.g., **1f**, **1g**, and **1p**) were prone to forming homocoupling products [e.g., **4f** (Scheme 3)], which resulted in low yields of **3**. Gratifyingly, adding 1,4-CHD (1,4-cyclohexadiene) as a more reactive hydrogen atom source significantly improved the yield of **3f** from 15% to 59% and decreased the level of competitive homocoupling (Scheme 2). Addition of **5** similarly improved the yields for α to O substrates [**3g** and **3p** (Scheme 2)]. However, 1,4-CHD does not help substrates with low reactivity (e.g., unchanged yield of 11% for **3d**) or ones that usually form a complex mixture of side products (e.g., no conversion for **3o**).

**Scheme 3:**
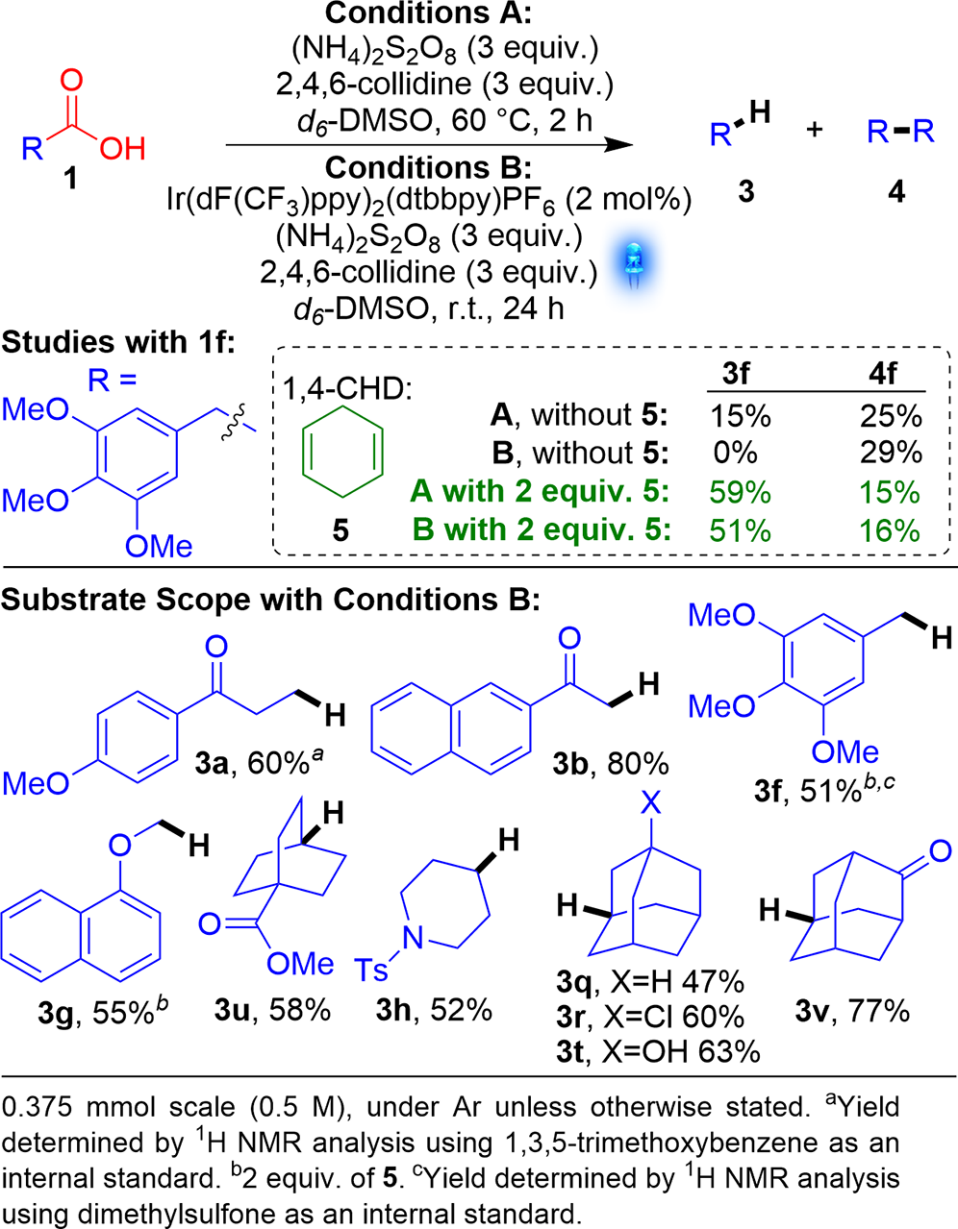
Studies using Photocatalytic Conditions

To further highlight the utility of our reaction, we applied it to a range of natural products, pharmaceuticals, and other biologically relevant molecules (Scheme 2). Fenofibric acid^17^ performs well to give 55% **3w** and was readily scaled to 1 g (50%). NSAIDs bendazac **1x** and naproxen **1y** both formed the desired **3x** and **3y** in 52% yield. Natural products **1z** and **1aa** performed well (58% **3z**, 71% **3aa**). Compound **3ab** formed smoothly from β-lacatmase docking fragment^18^**1ab** in 55% yield, whereas N-protected tranexamic acid^19^**1ac** decarboxylated smoothly to **3ac** in 50% yield. Finally, herbicide dichloroprop^20^**1ad** provided access to **3ad** in a reasonable yield of 49%.

During our attempts to improve the yields for benzylic or α to O substrates, we initially also developed a visible-light photocatalytic reaction (see the Supporting Information), as it was hoped that the milder reaction conditions (rt) would suppress the formation of homocoupling product **4f** (Scheme 3, conditions B). Unfortunately, the use of photocatalytic conditions B gave 0% **3f**. However, as with conditions A, adding 1,4-CHD significantly improved the yield of **3f** to 51%.

At this stage, we thought it prudent to perform a smaller substrate scope study using the photocatalytic conditions (Scheme 3). The yields from photocatalytic conditions, although decent to good (51–80%), were often significantly lower than for the corresponding thermal reactions (Scheme 2). Further investigation determined that this was due to conversions being limited by changes in homogeneity over the course of the reaction. This was confirmed by quantum yield measurements. The average quantum yield (ϕ) was 0.035; however, the ϕ for each reaction varied significantly and decreased with reaction time (see the Supporting Information). Therefore, the original thermal reaction (still mild at 40–60 °C) was deemed to have significant advantages over the photocatalytic reaction: better yields, metal- and light-free, operationally simple, and scalable.

Next, radical trapping experiments were conducted using **1a**. The desired hydrodecarboxylation reaction was totally inhibited in the presence of TEMPO and BHT (see the Supporting Information), indicating a radical-based mechanism.

Because no additional H atom source is required (except for benzylic and α to O substrates), we set out to elucidate the source of the H atoms in **3**. Initially, we investigated the potential of the various exchangeable protons within the reaction mixture to act as H atom sources; however, this possibility was quickly ruled out when hydrodecarboxylation still occurred smoothly with deuterated acid *d*-**1q** (see the Supporting Information). Next, investigations using *d*_9_-2,4,6-collidine **7** were carried out (Scheme 4A). In the presence of 95% deuterated **7**, only trace amounts of desired **3q** were observed with no D incorporation. In the presence of 85% deuterated **7**, the yield of **3q** correspondingly increased to ∼10%, again with no D incorporation. Examination of the bond dissociation energies (BDEs) would suggest that the benzylic C–H bonds of 2,4,6-collidine^21^ are the most likely to be abstracted by alkyl radical **VI**,^22^ although it is close to the limit. The inhibitory effect of **7** can be attributed to the increased strength of a C–D bond versus a C–H bond,^23^ suggesting that 2,4,6-collidine is the source of the H atoms. A similar inhibitory effect was observed with substrate **1a** (Scheme 4B). Finally, the reaction in Scheme 4A gives the same low yield of **3q** in nondeuterated DMSO, thus ruling out DMSO as the H atom source.

**Scheme 4:**
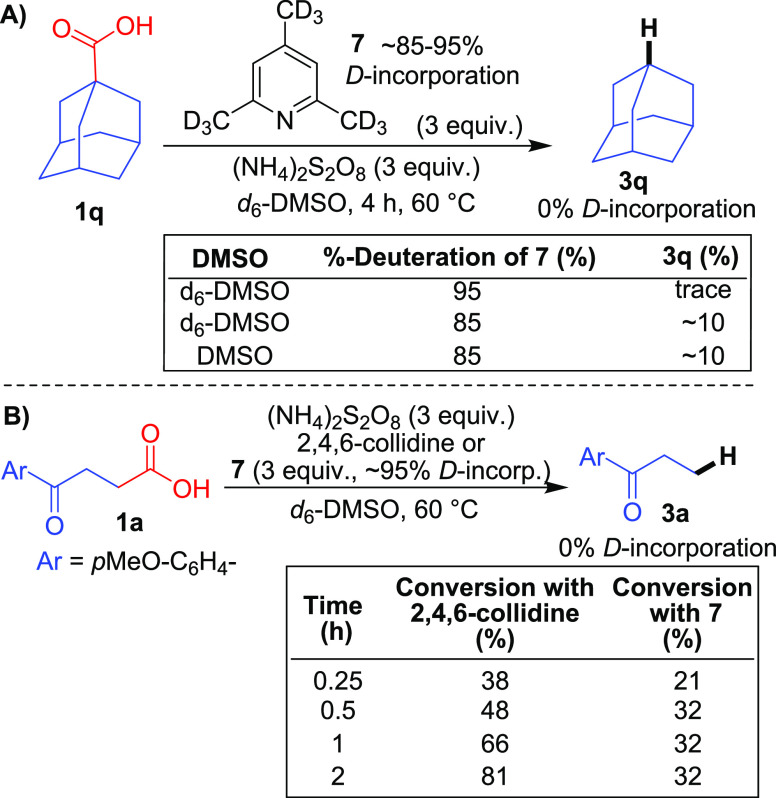
Deuterium Labeling Experiments

On the basis of the results presented above and the literature,^12a,12b,18^ we propose the following mechanism (Scheme 5). The reaction is initiated by formation of **I**. Meanwhile, persulfate anion **II** decomposes, in a process accelerated by the DMSO solvent,^13^ to give persulfate radical anion **III** (*E*_ox_ = +2.5–3.1 V vs SHE).^24^**III** can then carry out a single-electron oxidation of **I** (*E*_ox_ ≈ +1.25–1.31 V vs SCE)^8b^ to generate **V**, which quickly decomposes to release CO_2_ as well as alkyl radical **VI**. **VI** then undergoes HAT from 2,4,6-collidine to form product **3**. In cases in which the addition of 1,4-CHD is required, 1,4-CHD is the H atom source. In these cases, the improvement in yield can be rationalized as the C–H bond strengths in 1,4-cyclohexadiene^25^ are much weaker than those estimated for 2,4,6-collidine, allowing for competitive HAT versus undesired homocoupling.

**Scheme 5:**
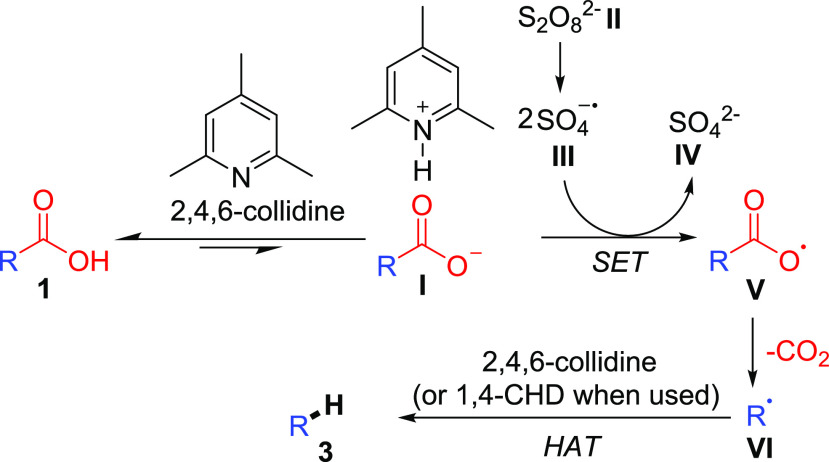
Proposed Mechanism

In conclusion, we have successfully developed a cheap, operationally simple, and scalable method for the hydrodecarboxylation of alkylcarboxylic acids, without the need for any metals or light. The reaction benefits from a broad functional group tolerance, crucially without the addition of an additional noxious or toxic hydrogen atom source. Mechanistic studies indicate that the intermediate radical abstracts H from the base (2,4,6-collidine) for normal substrates. Addition of 1,4-CHD is required only when more reactive radicals are formed, such as benzylic or α to O radicals, to reduce the level of competitive homocoupling.

## Supporting Information Available

Optimization studies, all of the experimental details, characterization, and copies of NMR data (PDF)
