# Supplementary material for: Direct Hydrodecarboxylation of Aliphatic Carboxylic Acids: Metal- and Light-Free
Source: Org Lett. 2022 Jan 7;24(2):686–91. doi: 10.1021/acs.orglett.1c04079 (PMC9007563; doi:10.1021/acs.orglett.1c04079)
Supplement: Supplementary file 1 — ol1c04079_si_001.pdf [file ol1c04079_si_001.pdf]

Direct Hydrodecarboxylation of Aliphatic Carboxylic Acids: Metal- and Light-Free

Euan B. McLean,[a] David T. Mooney,[a] David J. Burns[b] and Ai-Lan Lee [a,*]

[a]Institute of Chemical Sciences, Heriot-Watt University, Edinburgh EH14 4AS, Scotland, United Kingdom.
[b]Syngenta, Jealott's Hill International Research Centre, Bracknell, Berkshire RG42 6EY, United Kingdom.
*A.Lee@hw.ac.uk

SUPPORTING INFORMATION

Contents

1- General Considerations…S-2

2- Reaction Optimisation…S-3

3- Mechanistic Investigations…S-14

4- Substrate Synthesis…S-21

5- General Procedures for the Metal-, Light- and Catalyst-Free Hydrodecarboxylation of Carboxylic Acids…S-25

6- General Procedures for the Photoredox-Catalysed Hydrodecarboxylation of Carboxylic Acids…S-26

7- Product Characterisation…S-27

8- NMR Spectra…S-40

9- References…S-70

Supporting Information

General Considerations

^{1}H NMR spectra were recorded on a Bruker AV300 or AV400 spectrometer at 300 MHz or 400 MHz respectively and referenced to residual solvent. ^{13}C NMR spectra were recorded using the same spectrometers at 75 MHz or 101 MHz respectively. Chemical shifts (δ in ppm) were referenced to tetramethylsilane (TMS) or to residual solvent peaks ($CDCl_3$ at δ_H 7.26 ppm and δ_C at 77.0 ppm, d_6-acetone at δ_H 2.05 ppm and δ_C at 29.84 ppm and d_6-Dimethylsulfoxide at δ_H 2.50 ppm and δ_C at 39.5 ppm). *J* values are given in Hz and s, d, t, q, sept, dd, ddd, dddd, ddt and m are abbreviations corresponding to singlet, doublet, triplet, quartet, septet, doublet of doublets, doublet of doublets of doublets, doublet of doublets of doublets of doublets, doublet of doublets of triplets and multiplet respectively. Mass spectra were obtained at the University of Edinburgh. Infrared spectra were obtained on Perkin-Elmer Spectrum 100 FT-IR Universal ATR Sampling Accessory, deposited neat or as a chloroform solution to a diamond/ZnSe plate.

All reactions requiring irradiation by Blue LEDs unless otherwise stated, were carried out in a SynLED parallel photoreactor from Sigma-Aldrich (Z742680). The SynLED photoreactor has bottom-lit LEDs (465-470 nm) across a 4x4 reaction block array with consistent, static light intensity (130-140 lm, 1 W LED per vial) and angle (45°) with built-in cooling fan. Column chromatography was carried out using Matrix silica gel 60 from Fluorochem and TLC performed using Merck silica gel 60 F_{254} pre-coated sheets and visualised by UV (254 nm) and/or aqueous acidic $KMnO_4$. d_6-Dimethylsulfoxide was obtained from Cambridge Isotope Laboratories and used as received unless otherwise stated. Dimethylsulfoxide was obtained from Acros Organics and used as received unless otherwise stated. In cases where anhydrous solvents were required, both deuterated and otherwise, the solvent was distilled from calcium hydride or obtained from a MBraun SPS-800 solvent purification system (SPS) and stored under an argon atmosphere until use. All carboxylic acids **1** used other than those whose syntheses are detailed here were purchased from Fluorochem, TCI chemicals, Carbosynth, Acros or Sigma-Aldrich and were used as received. 2,4,6-Collidine and ammonium persulfate were purchased from Fluorochem and Sigma-Aldrich respectively and used as received. 1,4-Cyclohexadiene and $Ir(dF(CF_3)ppy)_2(dtbpy)PF_6$ were purchased from Alfa Aesar and Strem Chemicals respectively and were used as received.

Reaction Optimisation

Table S-1: Initial Discovery of 2,4,6-Collidine Promoted Reactivity

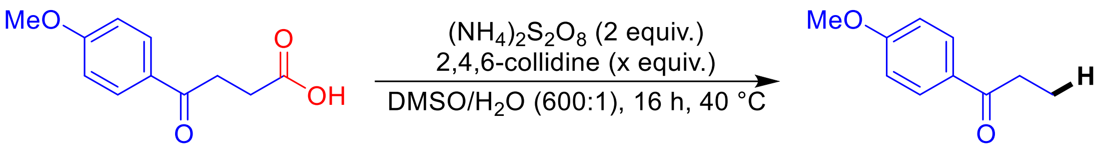

1a **3a**

Entry	x (equiv.)	3a (%)[a]
**1**	0	<5%
**2**	1	6
**3**	2	13
**4**	3	22
**5**	5	24
**6**	10	31

Reactions were carried out at 0.5 M concentration on a 0.375 mmol scale unless otherwise stated. [a]Yields were determined by ^1H NMR analysis of the crude reaction mixture using dimethylsulfone as an internal standard.

Studies were commenced using **1a** as a model carboxylic acid with the initial conditions used in entry 1 derived from conditions which had been used in decarboxylation reactions reported previously by our group.[1, 2] Under these initial conditions (Entry 1), no formation of desired decarboxylation product **3a** was observed. However, when 2,4,6-collidine was added to the reaction mixture as a Minisci substrate mimic with all reactive sites blocked, the formation of product **3a** could then be observed (Entries 2-6).

Table S-2: Additive and Base Screening

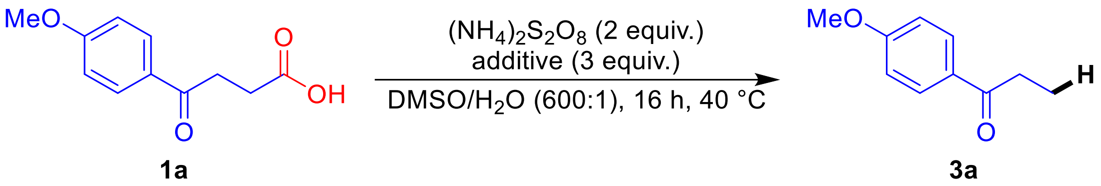

Entry	Additive	1a (%)[a]	3a (%)[a]
**1**	2,4,6-collidine	<5%	22
**2**	Na_2CO_3	5%	11
**3**	2-methylbenzothiazole	28%	>5
**4**	DBU	<5%	15
**5**	K_2HPO_3	<5%	13
**6**	KOAc	<5%	>5
**7**[b,c,d]	pyridine	<5%	5
**8**[b,c,d]	2,6-lutidine	27	22

Reactions were carried out at 0.5 M concentration on a 0.375 mmol scale unless otherwise stated. [a]Yields were determined by ^1H NMR analysis of the crude reaction mixture using dimethylsulfone as an internal standard. [b]Reactions carried out using 3 equivalents of 2,4,6-collidine. [c]Reactions use d_6-DMSO as the solvent. [d]Reactions carried out at 60 °C for 2 h.

Having discovered that the formation of desired hydrodecarboxylation product **3a** can be promoted by the addition of 2,4,6-collidine, we conducted further experiments with different bases and additives in order to determine both the role of 2,4,6-collidine in the reaction and also to improve the yield of desired product **3a**. No improvement could be made on 2,4,6-collidine as a promoter for the reaction, despite testing a wide range of additives. The failure of the reaction with 2-methylbenzothiazole (Entry 3), another Minisci substrate with all of its reactive sites blocked, to produce desired product **3a** in any detectable quantities coupled with the observation that other bases were able to promote the hydrodecarboxylation indicated to us that 2,4,6-collidine was acting as a base in the transformation and that the mechanism was proceeding *via* an intermediate carboxylate salt **I**.

At this point in the optimisation process, it was discovered that product **3a** was volatile under reduced pressure and that the yields determined by ^1H NMR analysis may not be entirely accurate. For these reasons, all experiments from this point onward were carried out in d_6-DMSO to allow for analysis of the crude reaction mixture without the need for an aqueous extraction prior to the analysis. This approach would also have the benefit of giving a more accurate representation of the amount of starting material left at the end of the reactions as it was possible that this was also being lost during the extraction process.

Non-deuterated DMSO was also initially used during our substrate scope studies. However, we encountered a problem that many of the products, upon hydrodecarboxylation, were surprisingly volatile, and we were initially getting low and irreproducible isolated yields, due to the volatility. Therefore, the decision was made to run them all in d_6-DMSO, so that the reaction could be monitored via ^{1}H NMR and to ensure that the isolated yield we obtained matched the suggested yield indicated by ^{1}H NMR analysis of the crude, otherwise it meant that we were losing compound due to volatility (or sometimes also water solubility). We then also repeated several examples using normal DMSO (e.g. **3q**, **3w**) to prove that the reaction reacts just as well under normal DMSO.

Effect of Temperature on the Progress of the Reaction

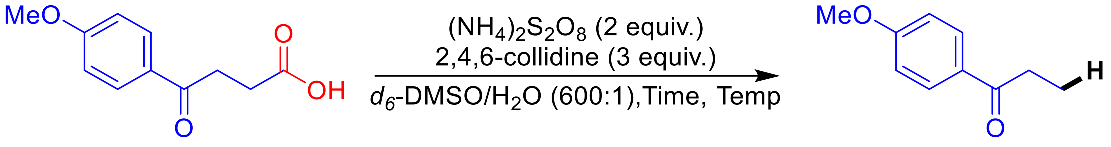

1a **3a**

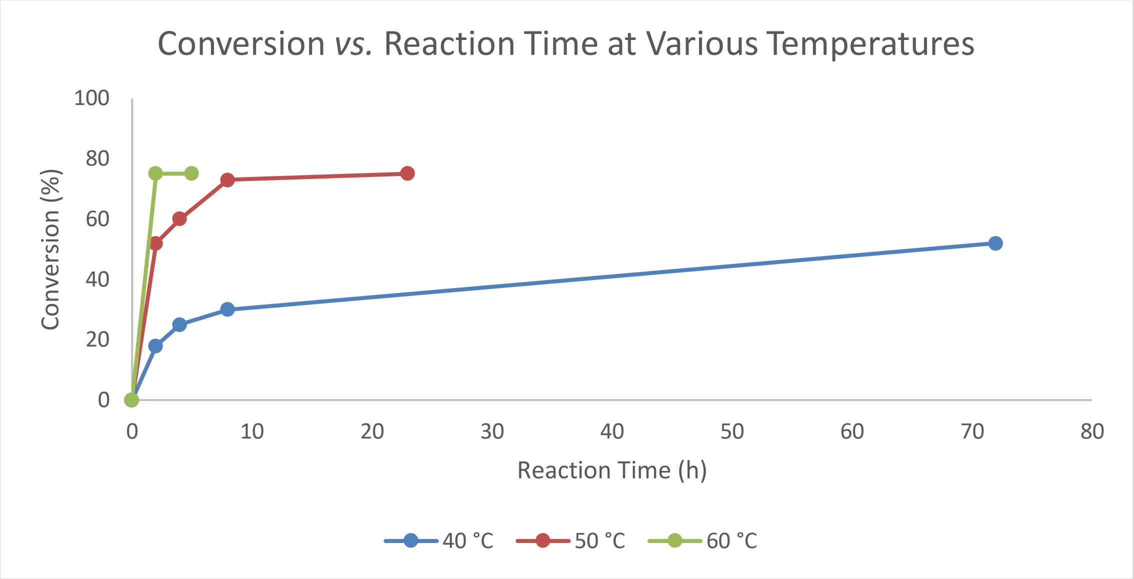

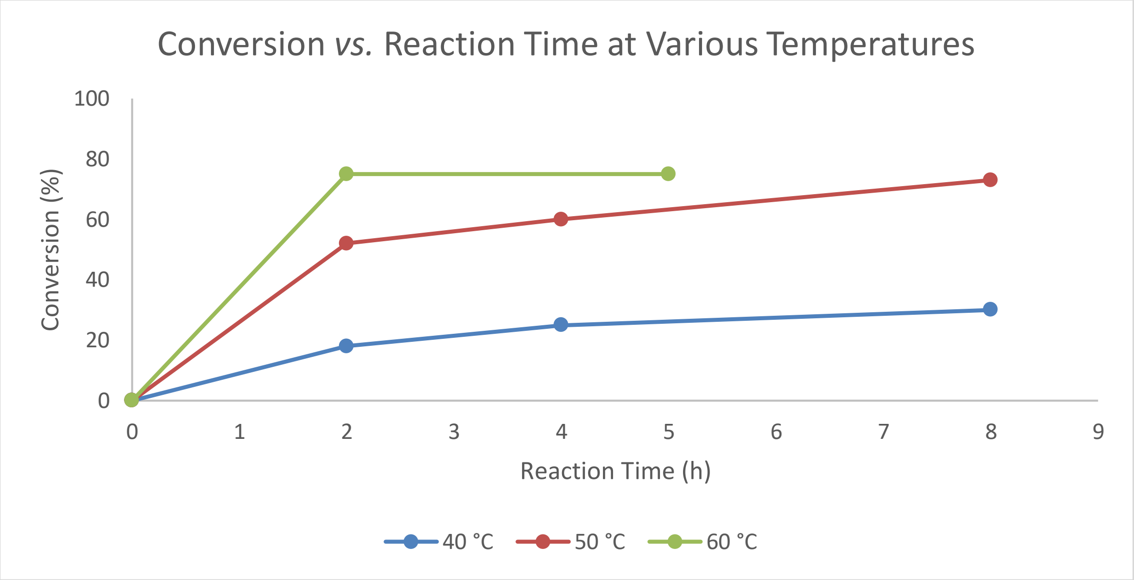

Figure S-2: Effect of temperature on reaction conversion (Top: full view of plot over all data points collected. Bottom: expanded view over initial 8 h period).

Carrying out the reaction in deuterated solvent allowed us to observe that in all the previous reactions with 2,4,6-collidine most of the starting material **1a** was left unreacted at the end of the reaction. With this new information we embarked on a basic kinetic investigation of

the reaction monitoring the ^1H NMR conversion over time. From these experiments we observed that under the initial reaction conditions at 40 °C the initial rate of the reaction was very fast, but this rate slowed dramatically after an initial period after which the reaction proceeds very slowly reaching a disappointing conversion of 52% after 72 h.

We were pleased to observe that the conversion of the reaction could be improved by increasing the reaction temperature to 50 °C; at this temperature the reaction has a similar kinetic profile with an initial period where the reaction conversion increases very quickly before slowing eventually reaching 75% conversion after 24 h.

Increasing the reaction temperature further to 60 °C had a highly beneficial effect on the reaction allowing it to reach 75% conversion within 2 h. Unfortunately, no further increase in conversion could be obtained by either increasing the temperature or extending the reaction time for substrate **1a**. Nevertheless, the reaction temperature of 60 °C was taken forward as the optimum reaction temperature.

Table S-3: Investigating Stoichiometry Variation

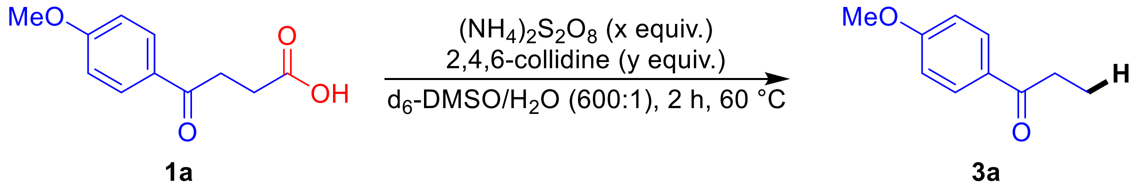

Entry	x	y	Conversion (%)
**1**	1.1	1.1	48
**2**	2	1.1	48
**3**	1.1	2	39
**4**	3	1.1	57
**5**	2	2	63
**6**	3	2	81
**7**	1.1	3	57

Reactions were carried out at 0.5 M concentration on a 0.375 mmol scale unless otherwise stated. Note: data shown were obtained during conversion monitoring experiments of the reaction under various reaction stoichiometries, therefore no yields are available as an internal standard was not added.

Investigation into the stoichiometry of both the $(NH_4)_2S_2O_8$ oxidant and 2,4,6-collidine showed that the equivalents of neither reagent could be lowered while still maintaining the same reaction performance. For this reason, 3 equivalents of 2,4,6-collidine and 2 equivalents of $(NH_4)_2S_2O_8$ were maintained as the optimum reaction stoichiometries.

Table S-4: Further Optimisation

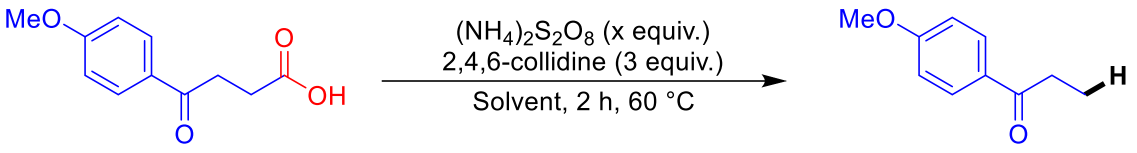

Entry	Solvent	x	1a (%)	3a (%)
**1**	d_6-DMSO/H_2O (600:1)	2	25[a]	57[a]
**2**	Anhydrous d_6-DMSO	2	23[a]	56[a]
**3**	d_6-DMSO	2	30[a]	60[a]
**4**[c]	d_6-DMSO	2	39	53
**5**	d_6-DMSO	3	23[b]	68[b]

Reactions were carried out at 0.5 M concentration on a 0.375 mmol scale unless otherwise stated. [a]Yields were determined by ^{1}H NMR analysis of the crude reaction mixture using dimethylsulfone as an internal standard. [b]Yields were determined by ^{1}H NMR analysis of the crude reaction mixture using 1,3,5-trimethoxybenzene as an internal standard. [c]Ammonium persulfate was added portion wise with 1 equivalent added at the start of the reaction and 1 equivalent added after 1 h.

Additional optimisation reactions showed that the amount of water present in the reaction solvent had very little influence on the outcome of the reaction. This contrasted with our group's previously published work on decarboxylative Minisci-type alkylation, acylations and carbamoylations which were improved when the solvent contained a small amount of water.[1,2] Finally, we observed that the yield of product **3a** could be improved by the addition of a further equivalent of $(NH_4)_2S_2O_8$.

Table S-5: Confirming the optimum reaction time.

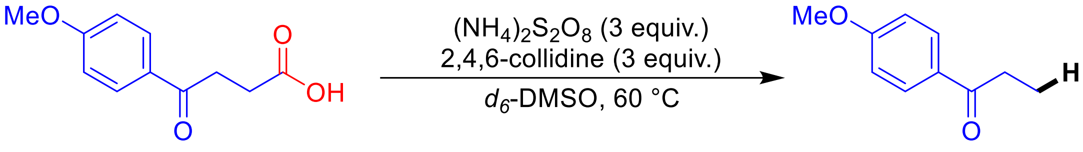

3.1a **3.4a**

Entry	Time (h)	Conversion (%)
**1**	0	0
**2**	0.25	38
**3**	0.5	48
**4**	1	66
**5**	2	81

Reactions were carried out at 0.5 M concentration on a 0.375 mmol scale, under an argon atmosphere unless otherwise stated.

The reaction requires the full two hours to reach the observed conversion at 60 °C.

Table S-6: Solvent Screen

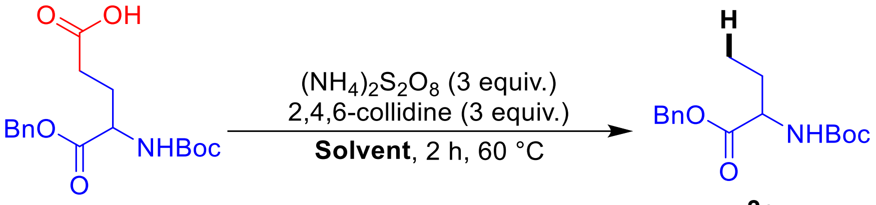

Entry	Solvent	1d (%)[b]	3e (%)
**1**	d_6-DMSO	-	59[a]
**2**	Sulfolane	Quant.	<5[b]
**3**	MeCN	Quant.	<5[b]
**4**	NMP	Quant.	<5[b]
**5**	DMF	Quant.	<5[b]
**6**	MeOH	Quant.	<5[b]

Reactions were carried out at 0.5 M concentration on a 0.375 mmol scale unless otherwise stated. [a]Isolated yield. [b]Yields were determined by ^{1}H NMR analysis of the crude reaction mixture using dimethylsulfone as an internal standard.

A solvent screen was carried out using the optimised conditions to determine if the hydrodecarboxylation reaction to form compound **3e** could occur in other solvents or if it was limited to DMSO. Our previous decarboxylative Minsici-type alkylation, acylations and carbamoylations worked exclusively in DMSO at mild temperatures.[1,2] The reason for this was thought to be that the rate at which $(NH_4)_2S_2O_8$ thermolyses is solvent dependent,[3] with the decomposition occurring much faster in DMSO allowing it to occur at milder temperatures.[4] As can be seen from the results above, a similar solvent dependence is observed in the hydrodecarboxylation of carboxylic acids such as **1e**.

Table S-7: Control Reactions

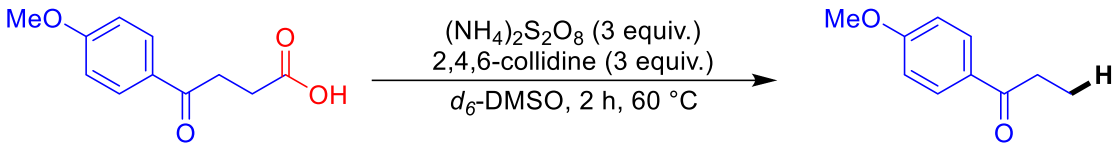

1a **3a**

Entry	Parameter Changed	1a (%)[a]	3a (%)[a]
**1**	No change	23	68
**2**	Under air	25	46
**3**	No $(NH_4)_2S_2O_8$	Quant.	<5
**4**	No 2,4,6-collidine	95	<5
**5**	In the dark	25	67
**6**	$Na_2S_2O_8$ instead of $(NH_4)_2S_2O_8$	23	27
**7**	$K_2S_2O_8$ instead of $(NH_4)_2S_2O_8$	31	21

Reactions were carried out at 0.5 M concentration on a 0.375 mmol scale unless otherwise stated. [a]Yields were determined by ^{1}H NMR analysis of the crude reaction mixture using 1,3,5-trimethoxybenzene as an internal standard.

In order to further understand the hydrodecarboxylation reaction, a series of control reactions were carried out. The results of these control reactions show that the reaction needs to be carried out under an inert atmosphere to occur efficiently (Entries 1-2). Additionally, the inclusion of both $(NH_4)_2S_2O_8$ and 2,4,6-collidine are vital for the desired reaction to take place (Entries 3-4). It was also demonstrated that the reaction performs equally well when carried out in the dark indicating that the reaction is promoted purely through temperature and that there is no photochemical aspect to this transformation (Entry 5).

Since metal-free hydrodecarboxylations of unsaturated carboxylic acids are known (ref. 11 in paper), the aim of our project was to develop the first metal-, catalyst- and light-free direct hydrodecarboxylation procedure for aliphatic carboxylic acids. Nevertheless, for completeness, an aromatic carboxylic acid example was also investigated under our standard conditions, which led to no conversion:

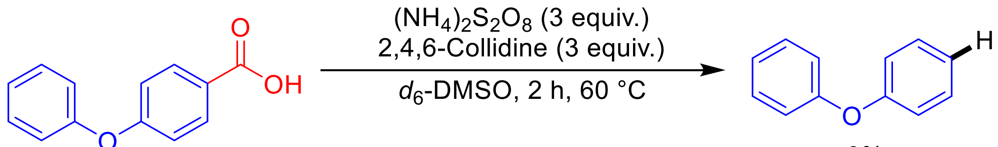

0%
quantitative recovery
of starting material

Table S-8: Photocatalyst Screening

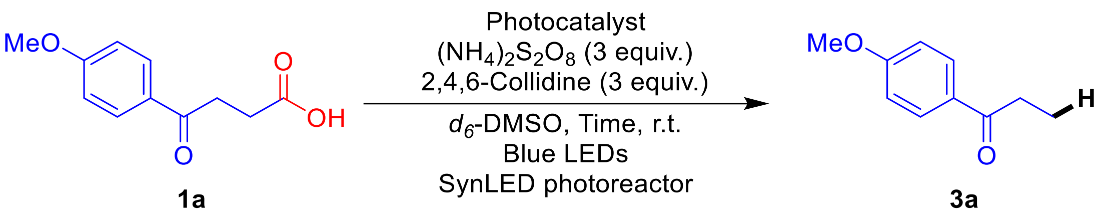

Entry	Photocatalyst	Catalyst Loading (mol%)	Time (h)	1a (%)[a]	3a (%)[a]
**1**	BTZ-Ph_2	5	2	90	10
**2**	BTZ-Ph_2	5	24	79	21
**3**	4CzIPN	5	2	95	5
**4**	4CzIPN	5	24	83	16
**5**	Fukuzumi's Catalyst	5	24	80	14
**6**	$Ir(dF(CF_3)ppy)_2(dtbpy)PF_6$	1	24	50	50
**7**	$Ir(dF(CF_3)ppy)_2(dtbpy)PF_6$	2	24	36	60
**8**	$Ru(bpy)_3PF_6(PF_6)_2$	2	24	77	10
**9**	$Ir(dF(CF_3)ppy)_2(dtbpy)PF_6$	3	24	49	48
**10**[b]	$Ir(dF(CF_3)ppy)_2(dtbpy)PF_6$	2	24	31	61
**11**[c]	$Ir(dF(CF_3)ppy)_2(dtbpy)PF_6$	2	24	38	48

Reactions were carried out at 0.5 M concentration on a 0.375 mmol scale unless otherwise stated. [a]Yields were determined by ^1H NMR analysis of the crude reaction mixture using 1,3,5-trimethoxybenzene as an internal standard. [b]Reaction carried out at 0.25 M. [c]Reaction carried out using 5 equivalents of 2,4,6-collidine and 5 equivalents of $(NH_4)_2S_2O_8$.

Having completed the optimisation of the thermally promoted hydrodecarboxylation reaction, we wanted to assess whether the reaction could also be promoted by photocatalysis in a manner that would be complementary to the thermal hydrodecarboxylation. With this goal in mind, we commenced a study into promoting the hydrodecarboxylation reaction using various photocatalysts under the irradiation of blue LEDs at ambient temperature. From these investigations it was shown that $Ir(dF(CF_3)ppy)_2(dtbpy)PF_6$ was the superior photocatalyst of those screened. Using this photocatalyst at a loading of 2 mol% allowed for comparable yields of product **3a** to those provided by the thermally promoted hydrodecarboxylation.

Table S-9: Photocatalysis Control Reactions

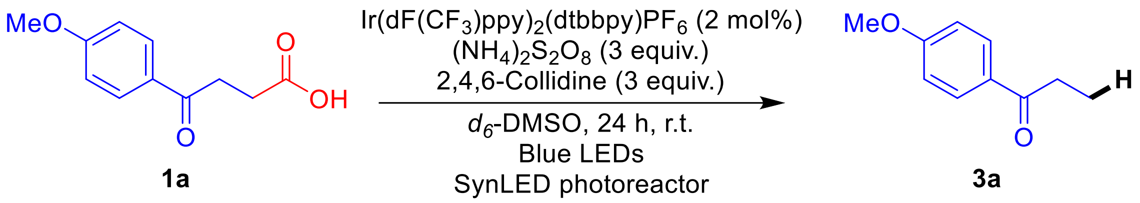

Entry	Parameter Changed	1a (%)[a]	3a (%)[a]
**1**	No change	36	60
**2**	No photocatalyst	58	29
**3**	No $(NH_4)_2S_2O_8$	Quant.	<5
**4**	No 2,4,6-collidine	Quant.	<5
**5**	In the dark	85%	15

Reactions were carried out at 0.5 M concentration on a 0.375 mmol scale unless otherwise stated. [a]Yields were determined by ^{1}H NMR analysis of the crude reaction mixture using 1,3,5-trimethoxybenzene as an internal standard.

Similarly, to the thermally promoted hydrodecarboxylation reaction, the photoredox-catalysed protocol was subjected to a range of control reaction to obtain more information about the transformation. As was observed for the thermal reaction, it was shown that both $(NH_4)_2S_2O_8$ and 2,4,6-collidine are vital for the transformation to take place (Entries 3-4). Somewhat surprisingly, some formation of product **3a** is observed in both the reaction with no photocatalyst (Entry 2) and with no light irradiation (Entry 5). Both reactions were run in parallel with the other controls in the same photoreactor with the dark reaction vessel wrapped in aluminium foil to prevent any light irradiation of the reaction mixture. The observation of some product **3a** in the dark reaction can be rationalised as the residual heat produced by the reaction vessel may be sufficient to heat the reaction enough to thermalyse enough of the $(NH_4)_2S_2O_8$ to promote formation of **3a**. In the case of the reaction in the absence of photocatalyst, in addition to the residual heat generated from the photoreactor, the intense blue LED irradiation from the photoreactor may also be sufficient break down the persulfate and promote the reaction to form product **3a**.

Mechanistic Investigations

Evidence for a Radical Decarboxylation Mechanism

In order to obtain more information about the reaction mechanism, a number of reactions were carried out with TEMPO and BHT as additives, which are known to be radical traps.

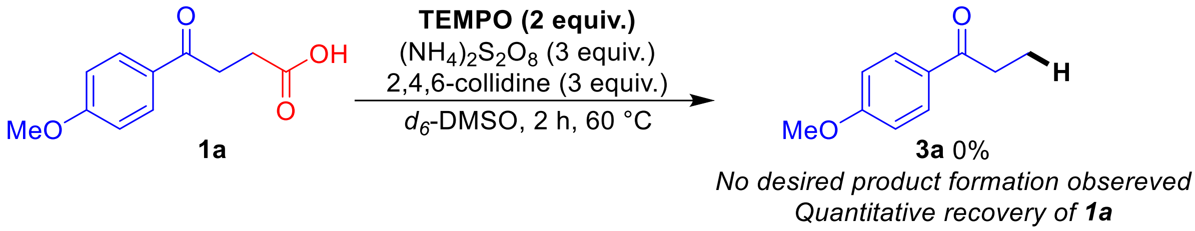

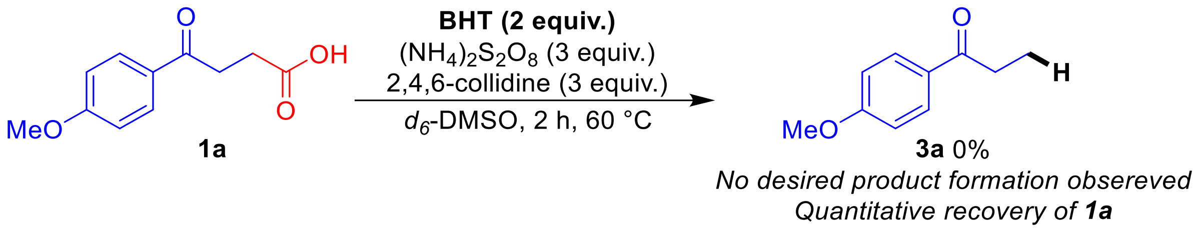

In the case of both reactions, the desired hydrodecarboxylation reaction was totally inhibited and no formation of product **3a** was observed (by ^{1}H NMR analysis). Quantitative recovery of unreacted starting material **1a** was observed in both cases.

Over the course of our investigations, it was observed that for highly reactive carboxylic acids such as benzylic carboxylic acid **1y** (naproxen), dimerised decarboxylation products **4y** and **4y'** could be observed in a 1:1 mixture of diastereomers.

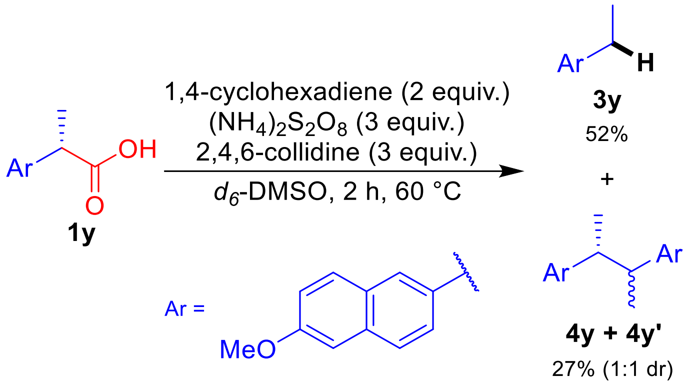

The results of both the radical trapping experiments and the observation of the formation of diasteromers **4y** and **4y'** strongly suggest that the hydrodecarboxylation reaction is proceeding *via* a radical mechanism.

Investigating the Mechanistic Origin of the Hydrogen Atom

A number of deuterium labelling experiments were carried out in order to determine what species is acting as the hydrogen atom source in the reaction. A possible source of hydrogen atoms would be from the exchangeable protons present in the reaction mixture. In order to investigate this, the following reaction was carried out.

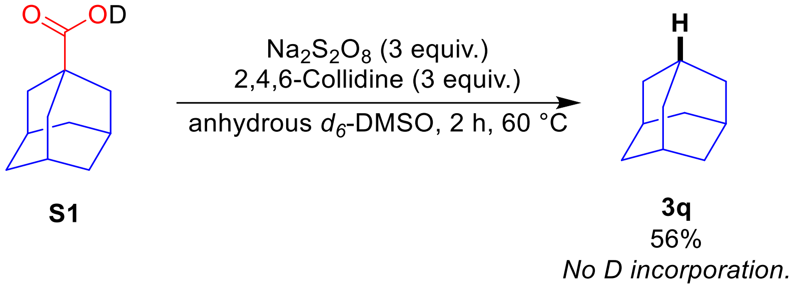

Carrying the reaction out using deuterated substrate **S1** and replacing $(NH_4)_2S_2O_8$ with $Na_2S_2O_8$ resulted in the formation of **3q** in a yield of 56% with no deuterium incorporation into the product. This result rules out the possibility of the hydrogen atom originating from either the $(NH_4)_2S_2O_8$ or the acidic proton on the carboxylic acid. It should be pointed out that rigorous drying of the solvent was necessary to prevent scrambling of labelled substrate **S1**.

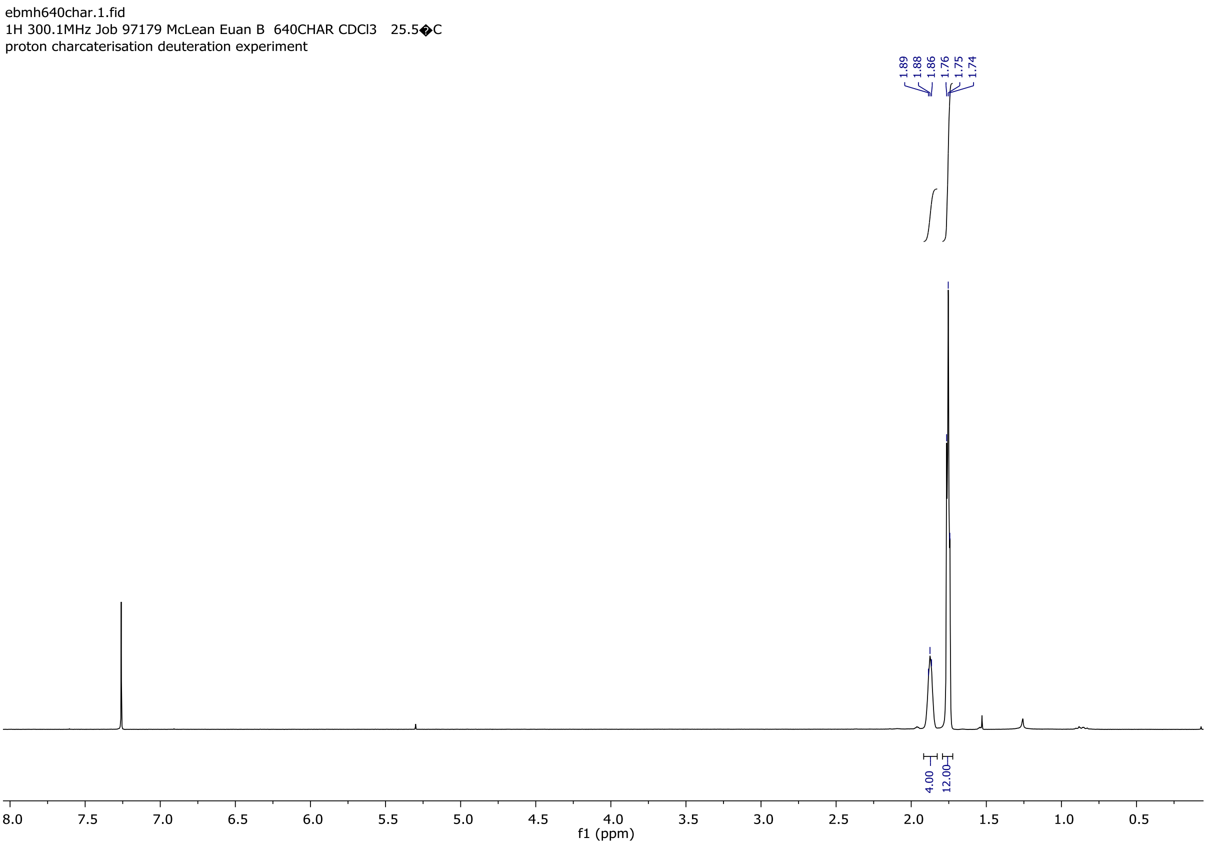

Quantum yield Determination for the Photoredox-Catalysed Oxidative Decarboxylation of Carboxylic Acids

The following model reaction was used in order to determine the quantum yield of the reaction (following the method used by Nicewicz and co-workers).[5]

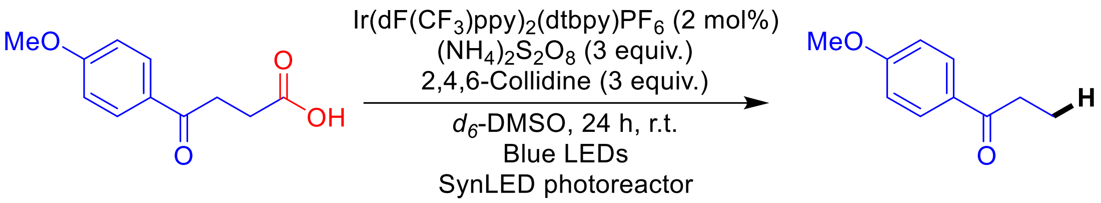

In the dark, potassium ferrioxalate trihydrate ($K_2Fe(C_2O_4)_3$) was prepared by adapting a previously reported literature procedure and was purified by recrystallization from water prior to use.[6] A 0.15 M aqueous solution of $K_2Fe(C_2O_4)_3$ was made up before 1.1 mL of the solution was irradiated in the SynLED photoreactor for 30 seconds (a second measurement was also carried out irradiating for 15 seconds and each measurement was duplicated and an average of the two absorbances taken). After irradiation, the samples were kept in the dark as much as possible. After being irradiated for the appropriate amount of time, 0.5 mL of each sample was transferred to a 25 mL volumetric flask. To this 5 mL of a buffered 1,10-phenanthroline solution, previously prepared in accordance with the literature,[7] was added and the flask made up to the mark with H_2O. The resulting solution was then stirred at room temperature for 30 minutes. 0.25 mL of the solution was then transferred to a cuvette and diluted to 2.75 mL with H_2O.

Carrying the procedure out in this way gave an average absorbance after 30 seconds of 0.1835 and after 15 seconds the average was 0.1560. With these absorbances, the Beer-Lambert law (equation 1) can then be applied to back calculate the concentration of Fe(II) present in the stock solutions since the path length of the cuvettes used for the UV/Visible spectroscopy was 1 cm.

Calculation for 30 seconds:

$$A = \varepsilon l c$$

$$c = \frac{A}{\varepsilon l}$$

$$c = \frac{0.1835}{11{,}110\ M^{-1}cm^{-1} \times 1\ \mathrm{cm}}$$

$$c = 1.652 \times 10^{-5}\ \mathrm{molL^{-1}}$$

This is the concentration present in the diluted solution used for the spectroscopic measurement. Back calculating for the original concentration gives:

$$c_1 v_1 = c_2 v_2$$

$$c_2 = \frac{1.652 \times 10^{-5}\ \text{mol L}^{-1} \times 2.75 \times 10^{-3}\ \text{L}}{250 \times 10^{-6}\ \text{L}}$$

$$c_2 = 1.817 \times 10^{-4}\ \text{molL}^{-1}$$

$$c_2 v_2 = c_3 v_3$$

$$c_3 = \frac{1.817 \times 10^{-5}\ \text{mol L}^{-1} \times 25 \times 10^{-3}\ \text{L}}{0.5 \times 10^{-3}\ \text{L}}$$

$$c_3 = 9.085 \times 10^{-3}\ \text{molL}^{-1}$$

Calculation for 15 seconds:

$$A = \varepsilon l c$$

$$c = \frac{A}{\varepsilon l}$$

$$c = \frac{0.1560}{11{,}110\ \text{M}^{-1}\text{cm}^{-1} \times 1\ \text{cm}}$$

$$c = 1.404 \times 10^{-5}\ \text{molL}^{-1}$$

This is the concentration present in the diluted solution used for the spectroscopic measurement. Back calculating for the original concentration gives:

$$c_1 v_1 = c_2 v_2 (7)$$

$$c_2 = \frac{1.404 \times 10^{-5}\ \text{mol L}^{-1} \times 2.75 \times 10^{-3}\ \text{L}}{250 \times 10^{-6}\ \text{L}}$$

$$c_2 = 1.544 \times 10^{-4}\ \text{molL}^{-1}$$

$$c_2 v_2 = c_3 v_3$$

$$c_3 = \frac{1.544 \times 10^{-4}\ \text{mol L}^{-1} \times 25 \times 10^{-3}\ \text{L}}{0.5 \times 10^{-3}\ \text{L}}$$

$$c_2 = 7.720 \times 10^{-3}\ \text{molL}^{-1}$$

The moles of tris-phenanthroline-Fe^{2+} complex ($\varepsilon_{510\ nm}$= 11110 $M^{-1}cm^{-1}$)[8] was then determined using UV/vis spectroscopy. The photon flux on the system was determined using the absolute quantum yield of 0.85 at 457.9 nm for the photodecomposition of $K_2Fe(C_2O_4)_3$.

From these concentrations the amount of incident photons for each irradiation time can be calculated as follows:

30 seconds:

$$\text{moles of Fe}^{2+}\text{in irradiated sample} = 9.085 \times 10^{-3}\ \text{molL}^{-1} \times 1.1 \times 10^{-3}\text{L}$$

$$\text{moles of Fe}^{2+}\text{in irradiated sample} = 9.994 \times 10^{-6}\ \text{mol}$$

$$\text{moles of incident photons} = 9.994 \times 10^{-6}\ \text{mol} \times \frac{1}{0.85}$$

$$\text{moles of incident photons} = 1.176 \times 10^{-5}\ \text{mol}$$

$$\text{photon flux} = \frac{1.176 \times 10^{-5}\ \text{mol}}{30\ \text{s}}$$

$$\text{photon flux} = 3.920 \times 10^{-7}\ \text{mols}^{-1}$$

15 seconds:

$$\text{moles of Fe}^{2+}\text{in irradiated sample} = 7.720 \times 10^{-3}\ \text{molL}^{-1} \times 1.1 \times 10^{-3}\text{L}$$

$$\text{moles of Fe}^{2+}\text{in irradiated sample} = 8.492 \times 10^{-6}\ \text{mol}$$

$$\text{moles of incident photons} = 8.492 \times 10^{-6}\ \text{mol} \times \frac{1}{0.85}$$

$$\text{moles of incident photons} = 9.991 \times 10^{-6}\ \text{mol}$$

$$\text{photon flux} = \frac{9.991 \times 10^{-6}\ \text{mol}}{15\ \text{s}}$$

$$\text{photon flux} = 6.661 \times 10^{-7}\ \text{mols}^{-1}$$

The photon flux averaged over the two experiments was determined to be 5.291×10^{-7} mol photons s^{-1} (std. dev. 1.938×10^{-7} mol photons s^{-1}). The quantum yield of the reaction was then obtained by stopping the reaction at varying degrees of conversion (reactions on a 0.375 mmol scale), using the following relationship:

$$\Phi_R = \frac{\text{moles of product}}{\text{moles of incident photons}}$$

NMR yield = 51%, reaction time 5 h

$$\Phi_R = \frac{\text{moles of product}}{\text{moles of incident photons}}$$

$$\Phi_R = \frac{\text{moles of product}}{\text{Photon flux } \times \text{reaction time}}$$

$$\Phi_R = \frac{0.191 \times 10^{-3}\ \text{mol}}{5.291 \times 10^{-7}\ \text{mols}^{-1} \times 18000\ \text{s}}$$

$$\Phi_R = 0.020$$

NMR yield = 46%, reaction time 3 h

$$\Phi_R = \frac{\text{moles of product}}{\text{moles of incident photons}}$$

$$\Phi_R = \frac{\text{moles of product}}{\text{Photon flux } \times \text{reaction time}}$$

$$\Phi_R = \frac{0.173 \times 10^{-3}\ \text{mol}}{5.291 \times 10^{-7}\ \text{mols}^{-1}\ \times 10800\ \text{s}}$$

$$\Phi_R = 0.030$$

NMR yield = 28%, reaction time 1 h

$$\Phi_R = \frac{\text{moles of product}}{\text{moles of incident photons}}$$

$$\Phi_R = \frac{\text{moles of product}}{\text{Photon flux } \times \text{reaction time}}$$

$$\Phi_R = \frac{0.105 \times 10^{-3}\ \text{mol}}{5.291 \times 10^{-7}\ \text{mols}^{-1}\ \times 3600\ \text{s}}$$

$$\Phi_R = 0.055$$

Taking the average of the three experiments gives the average quantum yield of the reaction as $\varphi = 0.035$ (std. dev. 0.018).

Proposed Mechanism for the Photoredox-Catalysed Hydrodecarboxylation Protocol

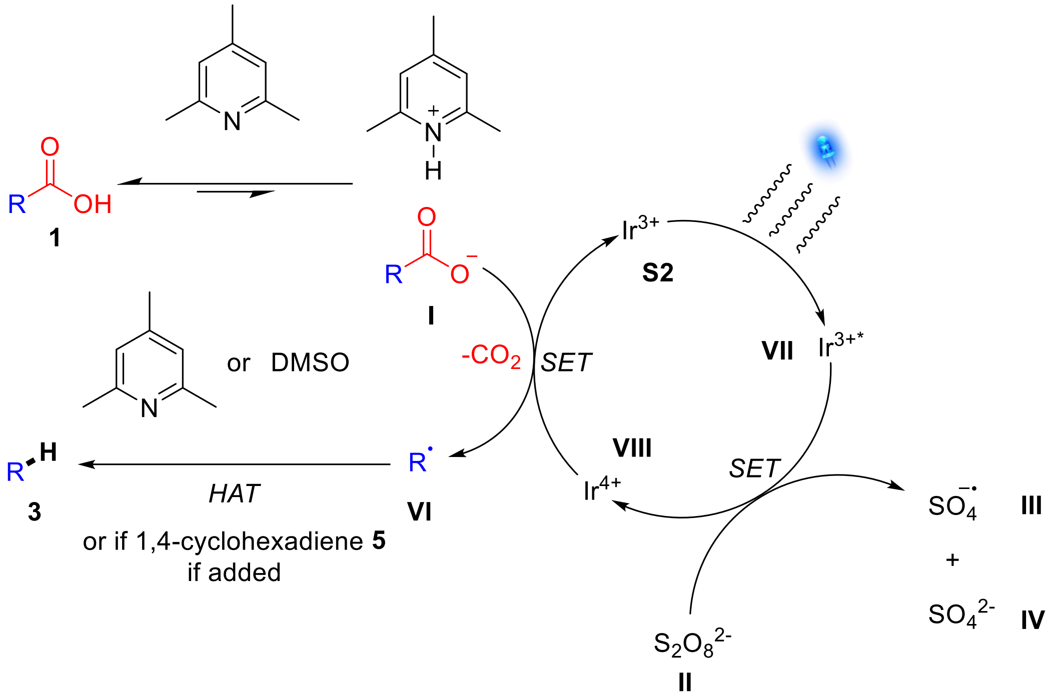

The photoredox-catalysed hydrodecarboxylation reaction commences with the establishment of the same carboxylic acid **1**/carboxylate **I** equilibrium seen in the metal- and light free-mechanism (Scheme 4). Meanwhile, excited state Ir(dF(CF_3)ppy)$_2$(dtbbpy)$^{+*}$ **VII** ($E_{1/2}^{Ir(III)*/Ir(IV)}$ = − 0.89 V *vs.* SCE)[9] is generated by the photoexcitation of the ground state complex **S2** by blue LED irradiation. Species **VIII** then undergoes a SET event with the persulfate anion **II** (E_{ox} = + 1.75 V *vs.* SCE)[10] to produce the powerful oxidising species Ir(dF(CF_3)ppy)$_2$(dtbbpy)$^{2+}$ **VIII** ($E_{1/2}^{Ir(IV)/Ir(III)}$ = + 1.69 V *vs.* SCE).[9] Intermediate **VIII** is then sufficiently oxidising to oxidise the carboxylate anion species **I** (E_{ox} = + 1.25-1.31 V *vs.* SCE)[11] to generate alkyl radical **VI**, after CO_2 extrusion, and regenerate ground state photocatalyst **S2**. The alkyl radical **VII** can then undergo the same hydrogen atom abstraction process as the metal- and light-free mechanism to furnish hydrodecarboxylated product **3**. It should also be noted that a parallel mechanism could be in operation, where the persulfate radical anion **III**, regenerated when excited sate iridium photocatalyst **VII** is oxidised to give species **VIII**, can also oxidise carboxylate intermediate **I** to produce the desired product **3** *via* carboxyl radical **VI**. This can happen by a similar mechanism to that which yields **3** in the metal- and light-free protocol (Scheme 4). Additionally, it has been shown previously in the literature by Stern-Volmer quenching studies that the Ir(dF(CF_3)ppy)$_2$(dtbbpy)PF_6 photocatalyst **S2** is quenched by the persulfate anion **II**[12] but not carboxylate anions like those in **I**.[13]

Starting Material Synthesis

Methyl 3-(naphthalen-2-yl)-3-oxopropanoate[14]

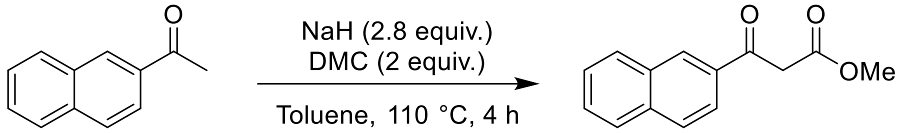

Following a procedure by Zhou,[14] to an oven-dried flask was added NaH (60% in mineral oil, 658 mg, 16.5 mmol, 2.8 equiv.) which was then subsequently dissolved in anhydrous toluene (40 mL). To this suspension was added dimethyl carbonate (1.06 g, 991 µL, 11.8 mmol, 2 equiv.) and the resulting solution was heated to reflux. Once at reflux, a solution of 2-acetonaphthone (1.00 g, 5.88 mmol, 1 equiv.) in anhydrous toluene (7 mL) was added slowly over a period of half an hour. Once the addition was complete, the mixture was heated at reflux for a further 4 h. Upon completion of the reaction, the reaction mixture was allowed to cool to room temperature before being quenched by the sequential addition of H_2O (10 mL) and glacial acetic acid (1.5 mL). Ice cold H_2O was then added until the resulting precipitate had dissolved, at this point the aqueous and organic layers were separated before the aqueous layer was extracted with EtOAc (2 × 20 mL). The combined organic layers were then washed with brine, dried over Na_2SO_4, filtered and concentrated at reduced pressure to give the title compound which was taken forward to the next step without further purification.

3-(Naphthalen-2-yl)-3-oxopropanoic acid (1b)[15]

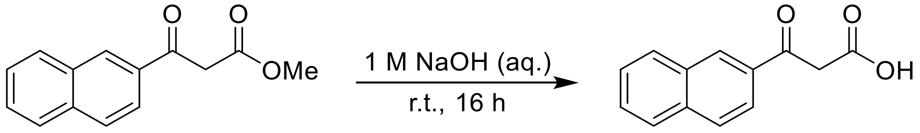

1b

Following a procedure by Wang,[16] to the crude methyl 3-(naphthalen-2-yl)-3-oxopropanoate from the previous step was added 1 M aqueous sodium hydroxide solution (10 mL). The resulting solution was then stirred vigorously for 16 h at room temperature. Upon completion of the reaction, the mixture was acidified by the addition of 6 M aqueous HCl solution. Dichloromethane (20 mL) was then added to the resulting suspension and the organic and aqueous layers were separated. The aqueous layer was then extracted with dichloromethane (3 × 20 mL), the combined organic layers were then dried over Na_2SO_4, filtered and the solvent removed at reduced pressure to give a crude solid which was then purified by recrystallisation from chloroform to give **1b** (486 mg, 2.29 mmol, 39%) as a white solid which forms a mixture of tautomers in solution with **1b** as the major tautomer.

ν_{max}/cm^{-1} 3052 (CO_2-H br.), 1725 (C=O), 1678 (C=O), 1591 (C=C Ar), 1568 (C=C Ar), 1505 (C=C Ar), 1467 (C=C Ar), 1463 (C=C Ar); **^{1}H NMR** (d_6-Acetone, 300 MHz) selected signals from major and minor tautomers: δ_H 13.39 (s, 1H, Minor), 11.14 (s, 1H, Major), 8.77 – 8.64 (m, 1H, Major), 8.57 – 8.44 (m, 1H, Minor), 6.00 (s, 1H, Minor), 4.24 (s, 2H, Major); **^{13}C NMR** (d_6-Acetone, 75 MHz) δ_C 193.8 (C), 175.7 (C), 173.1 (C), 169.2 (C), 136.7 (C), 135.7 (C), 134.7 (C), 133.8 (C),

133.5 (C), 131.6 (CH), 131.1 (CH), 130.6 (CH), 129.9 (CH), 129.7 (CH), 129.3 (CH), 129.3 (CH), 128.6 (CH), 128.6 (CH), 127.9 (CH), 127.7 (CH), 127.5 (CH), 124.6 (CH), 123.4 (CH), 87.8 (CH), 46.0 (CH_2) + one overlapping C; **m.p.** = 105-108 °C.

***N*-Boc tranexamic acid (1ac)**[17]

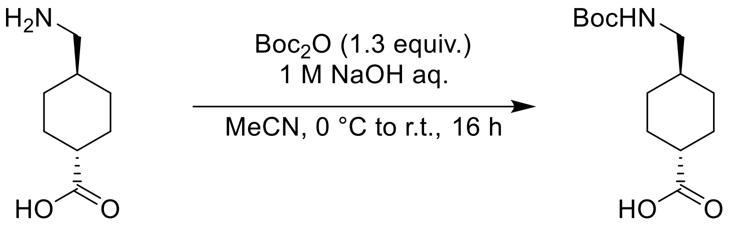

1ac

Following a patent,[17] a 1 M solution of NaOH in H_2O (11.5 mL) was cooled to 0 °C. To this solution, tranexamic acid (1.00 g, 6.36 mmol, 1 equiv.) was added and the resulting mixture diluted with MeCN (10 mL). To this solution, Boc_2O (1.80 g, 8.27 mmol, 1.3 equiv.) was added slowly portionwise and the resulting solution allowed to warm slowly to room temperature where it was stirred for 16 h. Upon completion of the reaction, any volatiles were removed at reduced pressure before the remaining aqueous solution was extracted with EtOAc (20 mL) which was set aside. The aqueous layer was then acidified with 1 M HCl aq. solution and extracted with EtOAc (3 × 30 mL). The combined organic layers were then washed with brine, dried over Na_2SO_4 and filtered before removal of the solvent at reduced pressure gave compound **1ac** (1.23 g, 4.77 mmol, 75%) as a white solid.

ν_{max}/cm^{-1} 3367 (N-H), 2981 (C-H), 2924 (C-H), 2860 (C-H), 1685 (C=O); ^1H NMR ($CDCl_3$, 300 MHz) δ_H 11.03 (br s, 1H), 4.58 (br s, 1H), 2.98 (t, *J* = 6.5 Hz, 2H), 2.26 (ddt, *J* = 12.3, 12.3, 3.5 Hz, 1H), 2.04 (m, 2H), 1.83 (m, 2H), 1.44 (m, 12H), 0.96 (m, 2H); **^{13}C NMR** ($CDCl_3$, 75 MHz) δ_C 181.1 (C), 156.1 (C), 79.2 (C), 46.5 (CH_2), 42.9 (CH), 37.7 (CH), 29.6 (CH_2), 28.4 (CH_3), 28.3 (CH_2); m.p. = 135-137 °C (lit.[18] 135 °C).

Adamantane-1-carboxylic acid-*d* (S1)

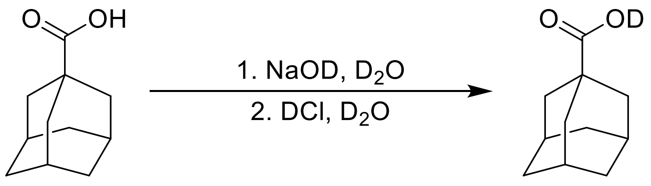

1q **S1**

Following a modified literature procedure by Nicewicz,[11] adamantane-1-carboxylic acid **1q** (252.4, 1.40 mmol) was suspended in D_2O (10 mL) under an argon atmosphere. To this mixture was added a 30% wt. solution of sodium deuteroxide in D_2O (1.35 g) and the resulting solution stirred for 0.5 h. After this time, the solution was acidified to pH 1 by the addition of

a 35% wt. solution of DCl in D_2O, the resulting precipitate was then filtered and dried under vacuum to give compound **S1** (242.9 mg, 1.34 mmol, 96%) as a white solid. Deuteration was confirmed by the disappearance of the O-H proton signal from the ^{1}H NMR, disappearance of the O-H stretching frequency and appearance of O-D stretching bands in the IR spectra.

ν_{max}/cm^{-1} 2904 (C-H), 2912 (C-H), 2851 (C-H), 2219-2073 (O-D), 1688 (C=O); **^{1}H NMR** ($CDCl_3$, 300 MHz) δ_H 2.07 – 1.97 (m, 3H), 1.94 – 1.86 (m, 6H), 1.80 – 1.63 (m, 6H); **^{13}C NMR** ($CDCl_3$, 75 MHz) δ_C 184.0 (C), 40.4 (C), 38.5 (CH_2), 36.4 (CH_2), 27.8 (CH); **m.p.** = 174-176 °C; **HRMS** HRMS data for these compounds cannot be obtained due to the exchangeability of the carboxylic acid O-D bond.[11]

***d*₉-2,4,6-Collidine (7)**[19]

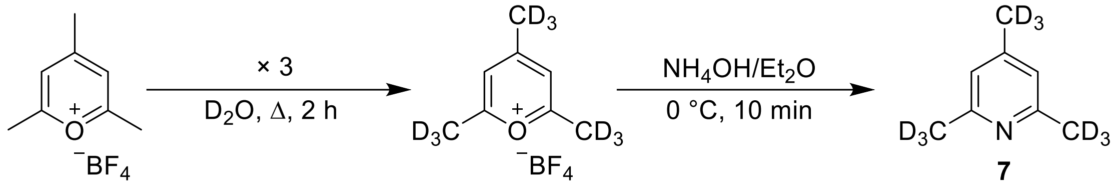

Following a literature procedure by Balaban,[19] 2,4,6-trimethylpyrilium tetrafluoroborate (2.36 g, 11.25 mmol) was dissolved in D_2O (2.25 g) under an argon atmosphere. The resulting solution was heated at reflux for 2 h after which the solution was cooled to 0 °C filtered and the solid dried under vacuum. This sequence was repeated a further two times.

The resulting solid was dissolved in a mixture of aqueous ammonia (10 mL) and Et_2O (10 mL) which had previously been cooled to 0 °C. The resulting solution was stirred at 0 °C for 10 minutes. After this time, the aqueous and organic layers were separated, and the organic layer extracted with 1 M aqueous HCl solution (10 mL). The aqueous layer was then washed with Et_2O (10 mL) before being made alkaline by the addition of 1 M aqueous NaOH solution. The alkaline solution was then extracted with Et_2O (2 × 20 mL), the organic layer was then dried over Na_2SO_4, filtered and concentrated at reduced pressure to give compound **7** (274.5 mg, 2.14 mmol, 19%) as a yellow oil. The deuterium incorporation into the product was measured to be approximately 95% by ^{1}H NMR analysis.

ν_{max}/cm^{-1} 2210 (C-D), 2158 (C-D), 2064 (C-D), 1603 (C=C Ar), 1558 (C=C Ar); **^{1}H NMR** ($CDCl_3$, 300 MHz) δ_H 6.77 (s, 2H); **^{13}C NMR** ($CDCl_3$, 75 MHz) δ_C 157.3 (C), 147.2 (C), 121.2 (CH), 23.5 (sept, J = 19.9 Hz, CH_3), 19.8 (sept, J = 19.3 Hz, CH_3).

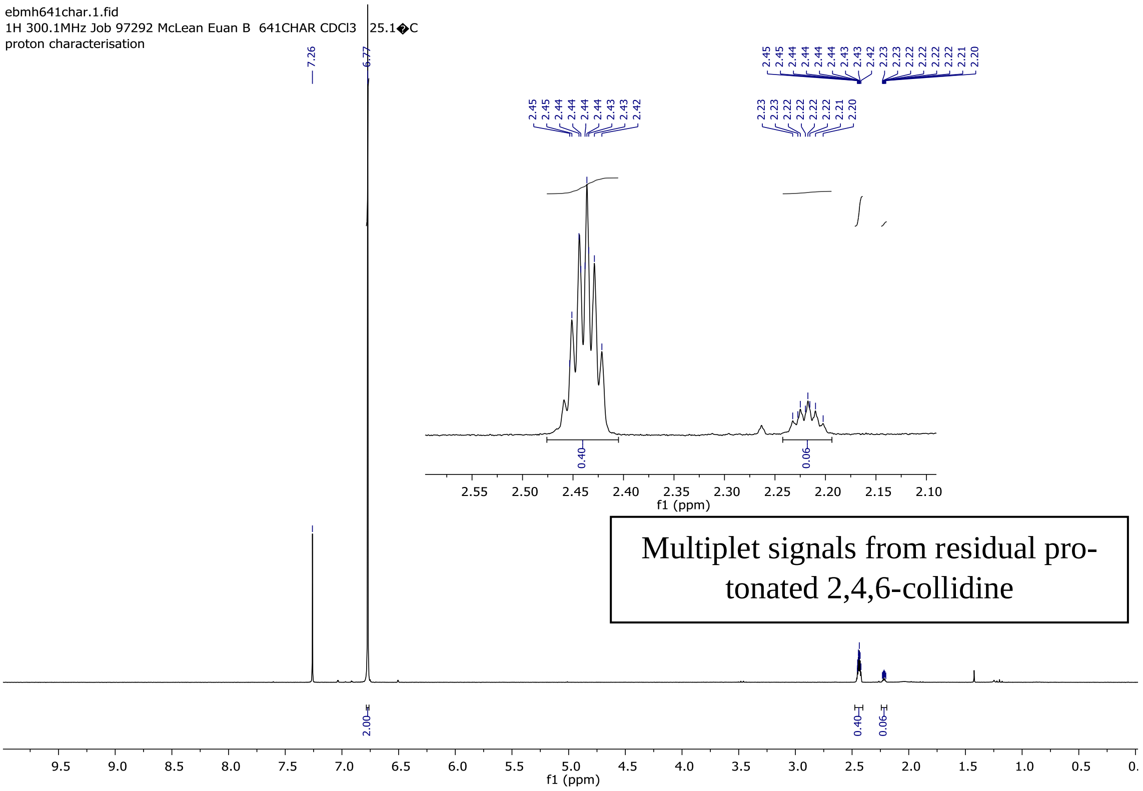

Residual signals integrate to ~5% of values expected for non-deuterated signals.

General Procedures for the Metal-, Light- and Catalyst-Free Hydrodecarboxylation of Carboxylic Acids

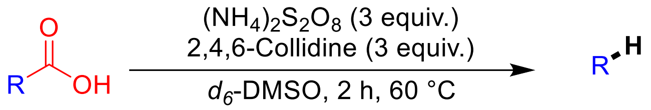

1 **3**

General Procedure A:

An oven-dried vial was charged with the desired carboxylic acid **1** (0.375 mmol, 1 equiv.), $(NH_4)_2S_2O_8$ (256.7 mg, 1.125 mmol, 3 equiv.) and 2,4,6-collidine (136 mg, 148 µL 1.125 mmol, 3 equiv.). The reagents were then dissolved in d_6-dimethylsulfoxide (0.75 mL). The resulting solution was then deoxygenated by bubbling with argon for 5 minutes before the vial was sealed and placed in a heating block heated at 60 °C where it was stirred for 2 h. Upon completion of the reaction, the reaction mixture was diluted with dichloromethane (20 mL) and washed with saturated sodium chloride solution (70 mL). The aqueous phase was then re-extracted with dichloromethane (3 × 20 mL), the combined organic layers were then washed with brine, dried over Na_2SO_4 and filtered. The solvent was then removed at reduced pressure before the resulting crude residue was purified using silica gel flash column chromatography to yield the products **3**.

Note that d_6-dimethylsulfoxide was used for ease of ^{1}H NMR monitoring. Dimethylsulfoxide works just as well as a solvent if ^{1}H NMR monitoring is not required.

General Procedure B:

Same as for general procedure A but after bubbling through argon, 1,4-cyclohexadiene (60.1 mg, 71 µL, 2 equiv.) was added prior to heating the reaction.

General Procedure C:

Same as for general procedure A but reacted at 40 °C for 16 h.

General Procedures for the Photoredox-Catalysed Base-Promoted Oxidative Decarboxylation of Carboxylic Acids

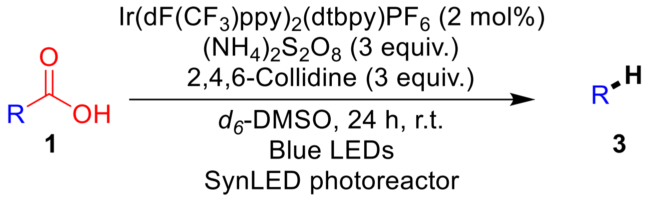

General Procedure D:

An oven-dried 4 mL glass vial was charged with the desired carboxylic acid **1** (0.375 mmol, 1 equiv.), $(NH_4)_2S_2O_8$ (256.7 mg, 1.125 mmol, 3 equiv.) and 2,4,6-collidine (136 mg, 148 µL 1.125 mmol, 3 equiv.) and $Ir(dF(CF_3)ppy)_2(dtbpy)PF_6$ (8.4 mg, 0.0075 mmol, 0.02 equiv). The reagents were then dissolved in d_6-dimethylsulfoxide (0.75 mL). The resulting solution was then deoxygenated by bubbling through argon for 5 minutes before the vial was sealed and placed in a SynLED parallel photoreactor (from Sigma-Aldrich, bottom-lit LEDs (465-470 nm) with consistent static light intensity (130-140 lm, 1 W LED per vial) and angle (45°) and built-in cooling fan) in which it was irradiated under Blue LEDs and stirred for 24 h. Upon completion of the reaction, the reaction mixture was diluted with dichloromethane (20 mL) and washed with saturated sodium chloride solution (70 mL). The aqueous phase was then re-extracted with dichloromethane (3 × 20 mL), the combined organic layers were then washed with brine, dried over Na_2SO_4 and filtered. The solvent was then removed at reduced pressure before the resulting crude residue was purified using silica gel flash column chromatography to yield the products **3**.

General Procedure E:

Same as for general procedure D but after bubbling through argon, 1,4-cyclohexadiene (60.1 mg, 71 µL, 2 equiv.) was added prior to irradiation of the reaction mixture.

Product Characterisation

It should be noted that examination of the literature has shown that the melting points of some of the adamantane derived products synthesised in this work had a strong dependence on the solvent that the solid crystallised from. This is most likely due to the inclusion of some solvent in the crystal structure of the solid and explains why some of the melting points reported in this section differ from the reported literature values. In these cases, all other spectroscopic data were in agreement with the reported literature spectra.

1-(4-Methoxyphenyl)propan-1-one (3a)[20]

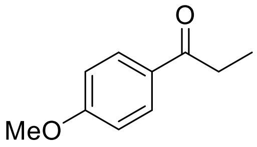

Following general procedure A, 4-(4-methoxyphenyl)-4-oxobutanoic acid **1a** (78.1 mg, 0.375 mmol) was used. ^{1}H NMR analysis of the crude reaction mixture using 1,3,5-trimethoxybenzene as an internal standard showed formation of the product **3a** in a yield of 68%. Due to the volatility of the product, an accurate isolated yield could not be determined. A pure sample of **3a** was obtained for characterisation purposes by purification of the crude by silica gel flash column chromatography (10:1 hexane/EtOAc) which gave product **3a** as a colourless oil.

Compound **3a** was also synthesised following general procedure D, using 4-(4-methoxyphenyl)-4-oxobutanoic acid **1a**. ^{1}H NMR analysis of the crude reaction mixture using 1,3,5-trimethoxybenzene as an internal standard showed formation of the product **3a** in a yield of 60%.

R_f 0.24 (10:1 hexane/EtOAc); **ν_{max}**/cm^{-1} 2975 (C-H Ar), 2937 (C-H), 2840 (C-H), 1678 (C=O), 1600 (C=C Ar), 1576 (C=C Ar), 1509 (C=C Ar) 1460 (C=C Ar); **^{1}H NMR** ($CDCl_3$, 300 MHz) δ_H 7.95 (d, J = 9.0 Hz, 2H), 6.93 (d, J = 9.0 Hz, 2H), 3.87 (s, 3H), 2.95 (q, J = 7.3 Hz, 2H), 1.21 (t, J = 7.3 Hz, 3H); **^{13}C NMR** ($CDCl_3$, 75 MHz) δ_C 199.5 (C), 163.3 (C), 130.2 (CH), 130.0 (C), 113.6 (CH), 55.4 (CH_3), 31.4 (CH_2), 8.4 (CH_3).

2-Acetonaphthone (3b)[21]

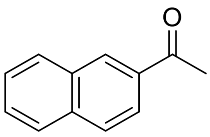

Following general procedure A, 3-(naphthalen-2-yl)-3-oxopropanoic acid **1b** (80.3 mg, 0.375 mmol) was used. The crude product was purified by silica gel flash column chromatography (20:1 pentane/Et_2O) to yield product **3b** (46.8 mg, 0.274 mmol, 73%) as a white solid.

Compound **3b** was also synthesised following general procedure D, using 3-(naphthalen-2-yl)-3-oxopropanoic acid **1b**. The crude product was purified by silica gel flash column chromatography (20:1 pentane/Et_2O to yield product **3b** (51.1 mg, 0.300 mmol, 80%) as a white solid.

R_f 0.25 (20:1 pentane/Et_2O) ν_{max}/cm^{-1} 3058 (C-H Ar), 3003 (C-H Ar), 2965 (C-H), 1675 (C=O), 1596 (C=C Ar), 1578 (C=C Ar), 1468 (C=C Ar); ^1H NMR ($CDCl_3$, 300 MHz) δ_H 8.49 – 8.45 (m, 1H), 8.04 (dd, *J* = 8.6, 1.8 Hz, 1H), 8.00 – 7.94 (m, 1H), 7.94 – 7.84 (m, 2H), 7.64 – 7.52 (m, 2H), 2.73 (s, 3H); ^{13}C NMR ($CDCl_3$, 75 MHz) δ_C 198.1 (C), 135.6 (C), 134.5 (C), 132.5 (C), 130.2 (CH), 129.5 (CH), 128.4 (CH), 128.4 (CH), 127.8 (CH), 126.8 (CH), 123.9 (CH), 26.7 (CH_3); m.p. = 54-56 °C (lit.[21] 54-56 °C).

Nonadecane (3c)[22]

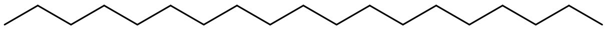

Following general procedure A, icosanoic acid **1c** (117.2 mg, 0.375 mmol) was used. The crude product was purified by filtration through a column of silica gel, eluting with pentane to yield product **3c** (35.5 mg, 0.131 mmol, 35%) as a white solid.

ν_{max}/cm^{-1} 2956 (C-H), 2920 (C-H), 2852 (C-H), 1466 (C-H), 1378 (C-H); ^1H NMR ($CDCl_3$, 400 MHz) δ_H 1.32 – 1.24 (m, 34H), 0.88 (d, *J* = 6.7 Hz, 6H); **^{13}C NMR** ($CDCl_3$, 101 MHz) δ_C 32.0 (CH_2), 29.7 (CH_2), 29.7 (CH_2), 29.4 (CH_2), 22.7 (CH_2), 14.1 (CH_3) + 4 overlapping CH_2s; **m.p.** = 28-30 °C (sub.) (lit.[23] 32 °C).

Benzyl 2-((*tert*-butoxycarbonyl)amino)butanoate (3e)[24]

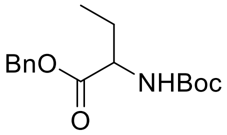

Following general procedure A, 5-(benzyloxy)-4-((*tert*-butoxycarbonyl)amino)-5-oxopentanoic acid **1e** (126.5 mg, 0.375 mmol) was used. The crude product was purified by silica gel flash column chromatography (12:1 hexane/EtOAc) to yield product **3e** (65.4 mg, 0.221 mmol, 59%) as a colourless oil.

R_f 0.21 (10:1 hexane/EtOAc) **ν_{max}**/cm^{-1} 3368 (N-H), 3034 (C-H Ar), 2974 (C-H), 2935 (C-H), 2879 (C-H), 1738 (C=O), 1711 (C=O), 1498 (C=C Ar), 1455 (C=C Ar), 1156 (=C-O-C); **^1H NMR** ($CDCl_3$, 300 MHz) δ_H 7.39 – 7.31 (m, 5H), 5.24 – 5.09 (m, 2H), 5.03 (d, *J* = 8.2 Hz, 1H), 4.37 – 4.25 (m, 1H), 1.94 – 1.77 (m, 1H), 1.77 – 1.59 (m, 1H), 1.44 (s, 9H), 0.90 (t, *J* = 7.5 Hz, 3H); **^{13}C NMR** ($CDCl_3$, 75 MHz) δ_C 172.6 (C), 155.3 (C), 135.5 (C), 128.6 (CH), 128.4 (CH), 128.2 (CH), 79.8 (C), 66.9 (CH_2), 54.6 (CH), 28.3 (CH_3), 25.9 (CH_2), 9.5 (CH_3).

1,2,3-Trimethoxy-5-methylbenzene (3f)[25]

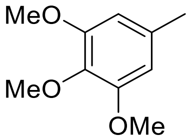

Following general procedure B, 2-(3,4,5-trimethoxyphenyl)acetic acid **3f** (84.8 mg, 0.375 mmol) was used. ^{1}H NMR analysis using dimethylsulfone as an internal standard and comparison with literature data[25] showed formation of **3f** in a yield of 59%. Product could not be separated from unidentifiable impurities despite the use of various purification techniques.

1-Methoxynaphthalene (3g)[26]

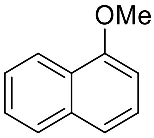

Following general procedure B, 2-(naphthalen-1-yloxy)acetic acid **3g** (75.8 mg, 0.375 mmol) was used. The crude product was purified by silica gel flash column chromatography (pentane→95:5 pentane/Et_2O) to yield product **3g** (40.8 mg, 0.259 mmol, 69%) as a colourless oil.

Compound **3g** was also synthesised following general procedure E, using 2-(naphthalen-1-yloxy)acetic acid **3g**. The crude product was purified by silica gel flash column chromatography (pentane→95:5 pentane/Et_2O) to yield product **3g** (32.7 mg, 0.206 mmol, 55%) as a colourless oil.

R_f 0.49 (pentane) **ν_{max}**/cm^{-1} 3052 (C-H), 3004 (C-H), 2954 (C-H), 2935 (C-H), 2847 (C-H), 2828 (C-H), 1580 (C=C Ar), 1509 (C=C Ar), 1463 (C=C Ar), 1451 (C=C Ar), 1266 (C-O-C), 1101 (C-O-C); **^{1}H NMR** ($CDCl_3$, 300 MHz) δ_H 8.37 – 8.23 (m, 1H), 7.88 – 7.77 (m, 1H), 7.56 – 7.37 (m, 4H), 6.84 (dd, *J* = 7.1, 1.4 Hz, 1H), 4.02 (s, 3H); **^{13}C NMR** ($CDCl_3$, 75 MHz) δ_C 155.4 (C), 134.4 (C), 127.4 (CH), 126.4 (CH), 125.8 (CH), 125.6 (C), 125.1 (CH), 121.9 (CH), 120.2 (CH), 103.7 (CH), 55.5 (CH_3).

1-Tosylpiperidine (3h)[27]

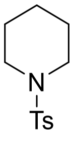

Following general procedure A, 1-tosylpiperidine-4-carboxylic acid **1i** (106.3 mg, 0.375 mmol) was used. The crude product was purified by silica gel flash column chromatography (4:1 hexane/EtOAc) to yield product **3h** (58.1 mg, 0.244 mmol, 65%) as an orange solid.

Compound **3h** was also synthesised following general procedure D, using 1-tosylpiperidine-4-carboxylic acid **1h**. The crude product was purified by silica gel flash column chromatography (4:1 hexane/EtOAc) to yield product **3h** (46.9 mg, 0.195 mmol, 52%) as an orange solid.

R_f 0.36 (4:1 hexane/EtOAc); **ν_{max}**/cm^{-1} 3026 (C-H Ar), 2945 (C-H), 2925 (C-H), 2828 (C-H), 1596 (C=C Ar), 1452 (C=C Ar); **^{1}H NMR** ($CDCl_3$, 300 MHz) δ_H 7.64 (d, *J* = 8.3 Hz, 2H), 7.31 (d, *J* = 8.3

Hz, 2H), 3.00 – 2.93 (m, 4H), 2.43 (s, 3H), 1.68 – 1.59 (m, 4H), 1.46 – 1.37 (m, 2H); **^{13}C NMR** ($CDCl_3$, 75 MHz) δ_C 143.2 (C), 133.3 (C), 129.5 (CH), 127.7 (CH), 46.9 (CH_2), 25.2 (CH_2), 23.5 (CH_2), 21.5 (CH_3); **m.p.** = 97-99 °C (lit.[28] 97-98 °C).

***tert*-Butyl cyclohexylcarbamate (3i)**[29]

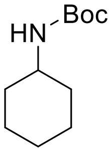

Following general procedure A, *trans-tert*-butoxycarbonyl)amino)cyclohexane-1-carboxylic acid **1i** (91.2 mg, 0.375 mmol) was used. The crude product was purified by silica gel flash column chromatography (4:1 pentane/Et_2O) to yield product **3i** (44.5 mg, 0.225 mmol, 60%) as a white solid.

R_f 0.43 (4:1 pentane/Et_2O); **v_{max}**/cm^{-1} 3362 (N-H), 2988 (C-H) 2973 (C-H), 2934 (C-H), 2853 (C-H); **^{1}H NMR** ($CDCl_3$, 300 MHz) δ_H 4.40 (br s, 1H), 3.52 – 3.32 (m, 1H), 1.97 – 1.85 (m, 2H), 1.75 – 1.63 (m, 2H), 1.63 – 1.53 (m, 1H), 1.44 (s, 9H), 1.41 – 1.24 (m, 2H), 1.22 – 1.00 (m, 3H); **^{13}C NMR** ($CDCl_3$, 75 MHz) δ_C 155.2 (C), 79.0 (C), 49.4 (CH), 33.6 (CH_2), 28.4 (CH_3), 25.6 (CH_2), 24.9 (CH_2); **m.p.** = 77-79 °C (lit.[29] 76-78 °C).

Methyl cyclohexanecarboxylate (3j)[30]

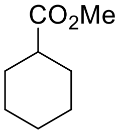

Following general procedure A, *trans*-4-(methoxycarbonyl)cyclohexane-1-carboxylic acid **1j** (69.8 mg, 0.375 mmol) was used. The crude product was purified by silica gel flash column chromatography (10:1 pentane/Et_2O) to yield product **3j** (38.6 mg, 0.270 mmol, 72%) as a yellow oil.

R_f 0.51 (10:1 pentane/Et_2O); **v_{max}**/cm^{-1} 2924 (C-H), 2855 (C-H), 1734 (C=O), 1169 (=C-O-C); **^{1}H NMR** ($CDCl_3$, 300 MHz) δ_H 3.66 (s, 3H), 2.30 (dddd, *J* = 11.2, 11.2, 3.7, 3.7 Hz, 1H), 1.95 – 1.84 (m, 2H), 1.80 – 1.70 (m, 2H), 1.68 – 1.58 (m, 1H), 1.45 (m, 2H), 1.35 – 1.18 (m, 3H); **^{13}C NMR** ($CDCl_3$, 75 MHz) δ_C 176.6 (C), 51.4 (CH_3), 43.1 (CH), 29.0 (CH_2), 25.8 (CH_2), 25.5 (CH_2).

1-Chloro-4-cyclohexylbenzene (3k)[31]

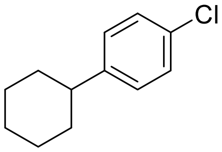

Following general procedure A, 4-(4-chlorophenyl)cyclohexane-1-carboxylic acid **1k** (89.5 mg, 0.375 mmol) was used. The crude product was purified by filtration through a column of silica gel, eluting with pentane to yield product **3k** (51.8 mg, 0.266 mmol, 71%) as a colourless oil.

ν_{max}/cm^{-1} 3054 (C-H Ar), 2922 (C-H), 2850 (C-H), 1492 (C=C Ar), 1448 (C=C Ar); **^{1}H NMR** ($CDCl_3$, 300 MHz) δ_H δ 7.25 (d, *J* = 8.5 Hz, 2H), 7.14 (d, *J* = 8.5 Hz, 2H), 2.54 – 2.40 (m, 1H), 1.92 – 1.71 (m, 5H), 1.48 – 1.34 (m, 4H), 1.33 – 1.19 (m, 1H); **^{13}C NMR** ($CDCl_3$, 75 MHz) δ_C 146.5 (C), 131.3 (C), 128.3 (CH), 128.2 (CH), 44.0 (CH), 34.4 (CH_2), 26.8 (CH_2), 26.1 (CH_2).

***tert*-Butyl pyrrolidine-1-carboxylate (3l)**[32]

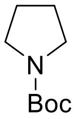

Following general procedure C, 1-(*tert*-butoxycarbonyl)pyrrolidine-3-carboxylic acid **1l** (80.7 mg, 0.375 mmol) was used. The crude product was purified by silica gel flash column chromatography (4:1 pentane/Et_2O) to yield product **3l** (32.1 mg, 0.188 mmol, 50%) as a colourless oil.

R_f 0.40 (4:1 pentane/Et_2O); **ν_{max}**/cm^{-1} 2973 (C-H), 2933 (C-H), 2874 (C-H), 1694 (C=O); **^{1}H NMR** ($CDCl_3$, 300 MHz) δ_H 3.45 – 3.14 (m, 4H), 1.96 – 1.71 (m, 4H), 1.46 (s, 9H); **^{13}C NMR** ($CDCl_3$, 75 MHz) δ_C 154.7 (C), 78.9 (C), 45.9 (CH_2), 45.6 (CH_2), 28.6 (CH_3), 25.8 (CH_2), 25.0 (CH_2).

(9*H*-Fluoren-9-yl)methyl azetidine-1-carboxylate (**3m**)

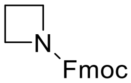

Following general procedure C, 1-(((9H-fluoren-9-yl)methoxy)carbonyl)azetidine-3-carboxylic acid **1m** (121.3 mg, 0.375 mmol) was used. The crude product was purified by silica gel flash column chromatography (9:1→17:3→4:1 hexane/EtOAc) to yield product **3m** (32.5 mg, 0.116 mmol, 31%) as a colourless oil.

R_f 0.18 (4:1 hexane/EtOAc); **ν_{max}**/cm^{-1} 3065 (C-H Ar), 3040 (C-H Ar), 3007 (C-H Ar), 2953 (C-H), 2886 (C-H), 1703 (C=O), 1477 (C=C Ar), 1468 (C=C Ar), 1450 (C=C Ar), 1414 (C=C Ar); ^{1}H NMR ($CDCl_3$, 300 MHz) δ_H 7.77 (m, 2H), 7.60 (m, 2H), 7.40 (m, 2H), 7.31 (ddd, *J* = 7.4, 1.3 Hz, 1.3 Hz, 2H), 4.36 – 4.30 (m, 2H), 4.26 – 4.19 (m, 1H), 4.08 (m, 4H), 2.34 – 2.22 (m, 2H); ^{13}C NMR ($CDCl_3$, 75 MHz) δ_C 156.3 (C), 144.0 (C), 141.3 (C), 127.6 (CH), 127.0 (CH), 125.2 (CH), 119.9 (CH), 66.9 (CH_2), 47.3 (CH), 15.8 (CH_2); **HRMS** Found (ESI-TOF) $[M+Na]^+$ 302.1154 $C_{18}H_{17}NO_2Na$ requires 302.1152.

***tert*-Butyl azepane-1-carboxylate (3n)**[32]

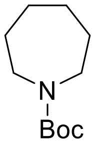

Following general procedure C, 1-(*tert*-butoxycarbonyl)azepane-4-carboxylic acid **1n** (91.2 mg, 0.375 mmol) was used. The crude product was purified by silica gel flash column chromatography (9:1 pentane/Et_2O) to yield product **3n** (21.1 mg, 0.105 mmol, 28%) as a colourless oil.

R_f 0.41 (9:1 hexane/Et_2O); **ν_{max}**/cm^{-1} 2974 (C-H Ar), 2929 (C-H), 2857 (C-H), 1692 (C=O); **^{1}H NMR** ($CDCl_3$, 300 MHz) δ_H 3.38 (t, *J* = 6.1 Hz, 2H), 3.32 (t, *J* = 5.9 Hz, 2H), 1.74 – 1.59 (m, 4H), 1.56 – 1.50 (m, 4H), 1.46 (s, 9H); **^{13}C NMR** ($CDCl_3$, 75 MHz) δ_C 155.7 (C), 78.9 (C), 46.9 (CH_2), 46.5 (CH_2), 28.5 (CH_3), 27.4 (CH_2), 26.8 (CH_2).

1-Ethoxy-4-methoxybenzene (3p)[33]

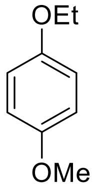

Following general procedure B, 2-(4-methoxyphenoxy)propanoic acid **3p** (73.6 mg, 0.375 mmol) was used. The crude product was purified by silica gel flash column chromatography (pentane→80:1 pentane/Et_2O) to yield product **3p** (37.7 mg, 0.248 mmol, 66%) as a white solid.

R_f 0.30 (80:1 pentane/Et_2O) **ν_{max}**/cm^{-1} 3045 (C-H), 2979 (C-H), 2937 (C-H), 2902 (C-H), 2833 (C-H), 1505 (C=C Ar), 1478 (C=C Ar), 1465 (C=C Ar), 1442 (C=C Ar), 1225 (C-O-C); **^{1}H NMR** ($CDCl_3$, 300 MHz) δ_H 6.83 (m, 4H), 3.99 (q, *J* = 7.0 Hz, 2H), 3.77 (s, 3H), 1.39 (t, *J* = 7.0 Hz, 3H); **^{13}C NMR** ($CDCl_3$, 75 MHz) δ_C 153.7 (C), 153.1 (C), 115.4 (CH), 114.6 (CH), 64.0 (CH_2), 55.7 (CH_3), 14.9 (CH_3); **m.p.** = 33-34 °C (lit.[33] 36-38 °C).

Adamantane (3q)[34]

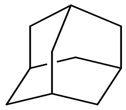

Following general procedure A, adamantane-1-carboxylic acid **1q** (67.6 mg, 0.375 mmol) was used. The crude was purified by filtration through a column of silica gel, eluting with pentane to yield product **3q** (36.7 mg, 0.270 mmol, 72%) as a white solid.

Compound **3q** was also synthesised following general procedure D using adamantane-1-carboxylic acid **1q**, the crude product was purified by filtration through a short column of silica gel, eluting with pentane to yield product **3q** (23.9 mg, 0.176 mmol, 47%) as a white solid.

Gram-scale Thermally Promoted Reaction

An oven-dried flask equipped with a stirrer bar was charged with adamantane-1-carboxylic acid **1q** (1.00 g, 5.55 mmol, 1 equiv.), ammonium persulfate (3.81 g, 16.7 mmol, 3 equiv.) and 2,4,6-collidine (2.02 g, 2.2 mL, 16.7 mmol), which were then dissolved in DMSO (11.1 mL) that

had previously been deoxygenated by bubbling through with argon for 20 minutes. The resulting solution was then deoxygenated by bubbling through argon for a further 10 minutes. The flask was then sealed with a stopper, placed in a heating block and heated at 60 °C for 2 h. Upon completion of the reaction, the reaction mixture was diluted with dichloromethane (30 mL). It was then washed with saturated sodium bicarbonate solution (100 mL), the aqueous layer was then extracted with dichloromethane (2 × 50 mL). The combined organic layers were then washed with 1 M aqueous HCl solution (100 mL). After the aqueous and organic layers had been separated the aqueous layer was again extracted with dichloromethane (2 x 50 mL). The combined organic layers were then washed with brine, dried over $MgSO_4$, filtered through a pad of silica gel and the solvent removed at reduced pressure to give compound **3q** as an off-white solid. Although pure by ^{1}H NMR, this compound was discoloured by some unknown residue, trituration of the compound with pentane followed by evaporation of the resulting solution gave product **3q** (501 mg, 3.72 mmol, 67%) as a white solid.

ν_{max}/cm^{-1} 2923 (C-H), 2899 (C-H), 2847 (C-H); **^{1}H NMR** ($CDCl_3$, 300 MHz) δ_H 1.90 – 1.84 (m, 4H), 1.78 – 1.72 (m, 12H); **^{13}C NMR** ($CDCl_3$, 75 MHz) δ_C 37.8 (CH_2), 28.3 (CH); **m.p.** = 164-166 °C (Sub.) (lit.[34] 204-207 °C).

1-Chloroadamantane (3r)[35]

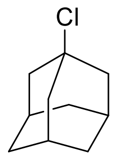

Following general procedure A, 3-chloroadamantane-1-carboxylic acid **1r** (80.5 mg, 0.375 mmol) was used. The crude product was purified by silica gel flash column chromatography (pentane) to yield product **3r** (45.9 mg, 0.270 mmol, 72%) as a white solid.

Compound **3r** was also synthesised following general procedure D, using 3-chloroadamantane-1-carboxylic acid **1r**. The crude product was purified by silica gel flash column chromatography (pentane) to yield product **3r** (38.1 mg, 0.225 mmol, 60%) as a white solid.

R_f 0.65 (pentane) **ν_{max}**/cm^{-1} 2907 (C-H), 2853 (C-H); **^{1}H NMR** ($CDCl_3$, 300 MHz) δ_H 2.16 – 2.12 (m, 9H), 1.70 – 1.65 (m, 6H); **^{13}C NMR** ($CDCl_3$, 75 MHz) δ_C 68.9 (C), 47.7 (CH_2), 35.6 (CH_2), 31.7 (CH); **m.p.** = 150-153 °C (lit.[36] 154-156 °C).

1-Bromoadamantane (3s)[37]

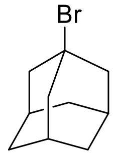

Following general procedure A, 3-bromoadamantane-1-carboxylic acid **1s** (97.2 mg, 0.375 mmol) was used. The crude product was purified by silica gel flash column chromatography (pentane) to yield product **3s** (40.0 mg, 0.188 mmol, 50%) as a white solid.

R$_f$ 0.64 (pentane) ν_{max}/cm^{-1} 2908 (C-H), 1455 (C-H), 1364 (C-H); **^{1}H NMR** ($CDCl_3$, 400 MHz) δ_H 2.38 – 2.35 (m, 6H), 2.13 – 2.08 (m, 3H), 1.75 – 1.72 (m, 6H); **^{13}C NMR** ($CDCl_3$, 101 MHz) δ_C 49.4 (CH_2), 37.8 (C), 35.6 (CH_2), 32.7 (CH); **m.p.** = 115-117 °C (lit.[37] 117-118 °C).

Adamantan-1-ol (3t)[38]

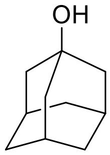

Following general procedure A, 3-hydroxyadamantane-1-carboxylic acid **1t** (73.6 mg, 0.375 mmol) was used. The crude product was purified by silica gel flash column chromatography (3:1 hexane/EtOAc) to yield product **3t** (45.1 mg, 0.296 mmol, 79%) as a white solid.

Compound **3t** was also synthesised following general procedure D, using 3-hydroxyadamantane-1-carboxylic acid **1t**. The crude product was purified by silica gel flash column chromatography (3:1 hexane/EtOAc) to yield product **3t** (35.8 mg, 0.236 mmol, 63%) as a white solid.

R$_f$ 0.28 (3:1 hexane/EtOAc) ν_{max}/cm^{-1} 3282 (O-H), 2914 (C-H), 2899 (C-H), 2848 (C-H); **^{1}H NMR** ($CDCl_3$, 300 MHz) δ_H 2.18 – 2.10 (m, 3H), 1.73 – 1.69 (m, 5H), 1.69 – 1.54 (m, 7H), 1.32 (s, 1H, OH); **^{13}C NMR** ($CDCl_3$, 75 MHz) δ_C 68.2 (C), 45.4 (CH_2), 36.1 (CH_2), 30.7 (CH); **m.p.** = 210 °C (sub.) (lit.[38] 288 °C).

Methyl bicyclo[2.2.2]octane-1-carboxylate (3u)[24]

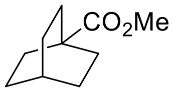

Following general procedure A, 4-(methoxycarbonyl)bicyclo[2.2.2]octane-1-carboxylic acid **1u** (79.6mg, 0.375 mmol) was used as the carboxylic acid. The crude product was purified by silica gel flash column chromatography (10:1 pentane/Et_2O) to yield product **3u** (55.1 mg, 0.326 mmol, 87%) as a colourless oil.

Compound **3u** was also synthesised following general procedure D, using 4-(methoxycarbonyl)bicyclo[2.2.2]octane-1-carboxylic acid **1u**. The crude product was purified by silica gel flash column chromatography (10:1 pentane/Et_2O) to yield product **3u** (36.8 mg, 0.218 mmol, 58%) as a colourless oil

R$_f$ 0.60 (10:1 pentane/Et_2O); ν_{max}/cm^{-1} 2946 (C-H), 2919 (C-H), 2865 (C-H), 1726 (C=O), 1242 (=C-O-C); **^{1}H NMR** ($CDCl_3$, 300 MHz) δ_H 3.62 (s, 3H), 1.77 – 1.68 (m, 6H), 1.64 – 1.52 (m, 7H); **^{13}C NMR** ($CDCl_3$, 75 MHz) δ_C 178.7 (C), 51.5 (CH_3), 38.3 (C), 28.0 (CH_2), 25.4 (CH_2), 23.7 (CH).

Adamantan-2-one (3v)[39]

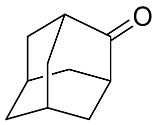

Following general procedure A, 4-oxoadamantane-1-carboxylic acid **1v** (72.8 mg, 0.375 mmol) was used as the carboxylic acid. The crude product was purified by silica gel flash column chromatography (2:1 pentane/Et_2O) to yield product **3v** (52.4 mg, 0.349 mmol, 93%) as a white solid.

Compound **3v** was also synthesised following general procedure D, using 0.375 mmol of 4-oxoadamantane-1-carboxylic acid **1v**. The crude product was purified by silica gel flash column chromatography (2:1 pentane/Et_2O) to yield product **3v** (43.1 mg, 0.289 mmol, 77%) as a white solid.

R_f 0.52 (2:1 pentane/Et_2O) **v_{max}**/cm^{-1} 2916 (C-H), 2855 (C-H), 1700 (C=O); **^{1}H NMR** ($CDCl_3$, 300 MHz) δ_H 2.57 – 2.50 (m, 2H), 2.13 – 1.89 (m, 12H); **^{13}C NMR** ($CDCl_3$, 75 MHz) δ_C 218.4 (C), 47.0 (CH), 39.2 (CH_2), 36.3 (CH_2), 27.4 (CH); **m.p.** = 211-213 °C (lit.[39] 224-225 °C).

(4-Chlorophenyl)(4-isopropoxyphenyl)methanone (3w)[40]

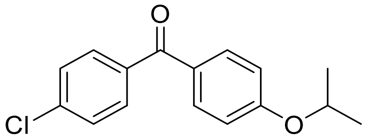

Following general procedure B, Fenofibric acid **1w** (119.5 mg, 0.375 mmol) was used. The crude product was purified by silica gel flash column chromatography (25:1 Hexane/EtOAc) to yield product **3w** (56.9 mg, 0.206 mmol, 55%) as a white solid.

Gram-scale Thermally Promoted Reaction

An oven-dried flask equipped with a stirrer bar was charged with fenofibric acid **1w** (1.00 g, 3.14 mmol, 1 equiv.), ammonium persulfate (2.15 g, 9.42 mmol, 3 equiv.) and 2,4,6-collidine (1.14 g, 1.24 mL, 9.42 mmol), which were then dissolved in dimethylsulfoxide (6.3 mL which had previously been deoxygenated by sparging with argon for 20 minutes). The resulting solution was then sparged with argon for a further 10 minutes before 1,4-cyclohexadiene (503 mg, 594 µL, 6.28 mmol, 2 equiv.) was added. The flask was then sealed with a stopper, placed in a heating block and heated at 60 °C for 2 h. Upon completion of the reaction, the reaction mixture was diluted with dichloromethane (30 mL). It was then washed with saturated sodium bicarbonate solution (100 mL) and the aqueous layer was then extracted with dichloromethane (2 × 50 mL). The combined organic layers were then washed with 1 M aqueous HCl solution (100 mL). After the aqueous and organic layers had been separated, the aqueous layer was again extracted with dichloromethane (2 x 50 mL). The combined organic layers were then washed with brine, dried over $MgSO_4$, filtered and the solvent removed at reduced pressure to give the crude product. Purification of the crude product by silica gel flash column chromatography (25:1 Hexane/EtOAc) gave compound **3w** (434 mg, 1.57 mmol, 50%) as a white solid.

R_f 0.23 (25:1 hexane/EtOAc) ν_{max}/cm^{-1} 3066 (C-H Ar), 2981 (C-H), 2876 (C-H), 1641 (C=O), 1599 (C=C Ar), 1569 (C=C Ar), 1483 (C=C Ar), 1417 (C=C Ar), 1252 (C-O-C), 1121 (C-O-C); **^{1}H NMR** ($CDCl_3$, 300 MHz) δ_H 7.78 (d, *J* = 8.9 Hz, 2H), 7.71 (d, *J* = 8.6 Hz, 2H), 7.44 (d, *J* = 8.6 Hz, 2H), 6.93 (d, *J* = 8.9 Hz, 2H), 4.67 (sept, *J* = 6.1 Hz, 1H), 1.38 (d, *J* = 6.1 Hz, 6H); **^{13}C NMR** ($CDCl_3$, 75 MHz) δ_C 194.2 (C), 161.9 (C), 138.2 (C), 136.6 (C), 132.5 (CH), 131.1 (CH), 129.3 (C), 128.5 (CH), 115.0 (CH), 70.2 (CH), 21.9 (CH_3); **m.p.** = 103-105 °C.

1-Benzyl-3-methoxy-1*H*-indazole (3x)[41]

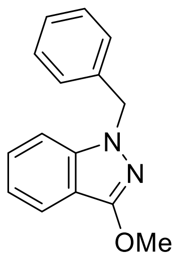

Following general procedure B, bendazac **1x** (105.9 mg, 0.375 mmol) was used. The crude product was purified by silica gel flash column chromatography (30:1 hexane/EtOAc) to yield product **3x** (46.8 mg, 0.195 mmol, 52%) as a yellow oil.

R_f 0.23 (30:1 hexane/EtOAc) ν_{max}/cm^{-1} 3086 (C-H Ar), 3062 (C-H Ar), 3029 (C-H Ar), 2932 (C-H), 1581 (C=C Ar), 1535 (C=C Ar), 1495 (C=C Ar), 1452 (C=C Ar), 1255 (C-O-C), 1101 (C-O-C); **^{1}H NMR** ($CDCl_3$, 300 MHz) δ_H 7.67 (ddd, *J* = 8.1, 1.1, 1.1 Hz, 1H), 7.35 – 7.23 (m, 4H), 7.21 – 7.14 (m, 3H), 7.04 (ddd, *J* = 9.0, 6.9, 0.9 Hz, 1H), 5.41 (s, 2H), 4.11 (s, 3H); **^{13}C NMR** ($CDCl_3$, 75 MHz) δ_C 156.5 (C), 141.7 (C), 137.4 (C), 128.6 (CH), 127.4 (CH), 127.4 (CH), 127.0 (CH), 120.0 (CH), 119.1 (CH), 112.9 (C), 108.8 (CH), 56.2 (CH_3), 52.3 (CH_2).

2-Ethyl-6-methoxynaphthalene (3y)[42]

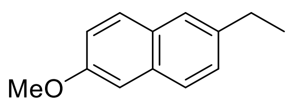

Following general procedure B, (S)-naproxen **1y** (86.3 mg, 0.375 mmol) was used. ^{1}H NMR analysis using dimethylsulfone as an internal standard, and comparison with literature data,[42] showed formation of **3y** in a yield of 52%. Product could not be separated from unidentifiable impurities despite the use of various purification techniques.

From the above reaction to synthesise compound **3y** the following compounds were also isolated:

6,6'-((2*R*,3*S*)-Butane-2,3-diyl)bis(2-methoxynaphthalene) (4y) and 6,6'-((2*S*,3*S*)-Butane-2,3-diyl)bis(2-methoxynaphthalene) (4y')[43]

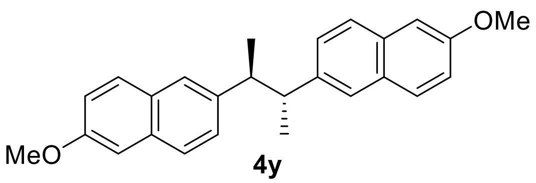

4y

+

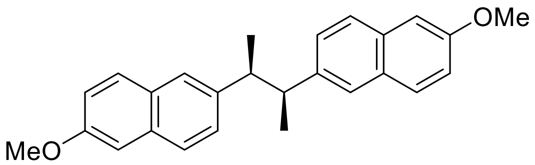

4y′

After the above procedure, purification of the crude reaction mixture by silica gel flash column chromatography (pentane→19:1 pentane/Et_2O) gave products **4y** and **4y′** in a 1:1 inseparable mixture of diastereomers (19.1 mg, 0.101 mmol, 27%) as a yellow solid.

R_f 0.30 (19:1 pentane/Et_2O); **ν_{max}**/cm^{-1} 3055 (C-H Ar), 2963 (C-H), 2935 (C-H), 2902 (C-H), 2838 (C-H), 1604 (C=C Ar), 1504 (C=C Ar), 1483 (C=C Ar), 1452 (C=C Ar), 1264 (C-O-C); **^{1}H NMR** ($CDCl_3$, 300 MHz) Selected signals of both diastereomers δ_H 3.93 (s, 6H 2 × CH_3), 3.88 (s, 6H), 3.21 (m, 2H), 3.11 – 2.95 (m, 2H), 1.43 – 1.30 (d, *J* = 6.7 Hz, 6H), 1.22 – 1.05 (d, *J* = 6.4 Hz, 6H,); **^{13}C NMR** ($CDCl_3$, 75 MHz) δ_C 157.2 (C), 157.1 (C), 141.7 (C), 141.0 (C), 133.3 (C), 133.0 (C), 129.1 (CH), 128.8 (C), 127.3 (CH), 126.8 (CH), 126.6 (CH), 126.1 (CH), 126.0 (CH), 125.9 (CH), 118.6 (CH), 118.4 (CH), 105.6 (CH), 105.5 (CH), 55.3 (CH_3), 55.2 (CH_3), 47.2 (CH), 46.1 (CH), 21.2 (CH_3), 17.9 (CH_3) + 1 overlapping CH and 1 overlapping C.

(1*R*,4*R*)-7,7-Dimethylbicyclo[2.2.1]heptan-2-one (3z)[44]

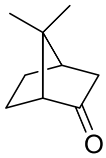

Following general procedure A, (+)-ketopinic acid **1z** (68.3 mg, 0.375 mmol) was used. The crude product was purified by silica gel flash column chromatography (2:1 pentane/Et_2O) to yield product **3z** (30.0 mg, 0.218 mmol, 58%) as a white solid.

R_f 0.70 (2:1 pentane/Et_2O) **ν_{max}**/cm^{-1} 2963 (C-H), 2886 (C-H), 1743 (C=O); **^{1}H NMR** ($CDCl_3$, 300 MHz) δ_H 2.47 – 2.34 (m, 1H), 2.14 – 1.90 (m, 4H), 1.78 (d, *J* = 18.2 Hz, 1H), 1.56 – 1.33 (m, 2H), 1.04 (s, 6H); **^{13}C NMR** ($CDCl_3$, 75 MHz) δ_C 218.6 (C), 58.4 (CH), 45.4 (C), 44.2 (CH_2), 43.5 (CH), 27.7 (CH_2), 22.8 (CH_2), 21.7 (CH_3), 20.7 (CH_3); **m.p.** = 103-105 °C.

(1*S*,4*R*)-4,7,7-Trimethyl-2-oxabicyclo[2.2.1]heptan-3-one (3aa)[45]

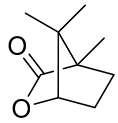

Following general procedure A, (-)-camphanic acid **1aa** (74.3 mg, 0.375 mmol) was used. The crude product was purified by silica gel flash column chromatography (2:1 pentane/Et_2O) to yield product **3aa** (40.8 mg, 0.266 mmol, 71%) as a white solid.

R_f 0.43 (2:1 pentane/Et_2O) **ν_{max}**/cm^{-1} 2968 (C-H), 2930 (C-H), 2888 (C-H), 1760 (C=O); **^{1}H NMR** ($CDCl_3$, 300 MHz) δ_H 4.32 (m, 1H), 2.05 – 1.88 (m, 1H), 1.87 – 1.73 (m, 2H), 1.69 – 1.54 (m, 1H), 1.09 (s, 3H), 1.02 (s, 3H), 0.92 (s, 3H); **^{13}C NMR** ($CDCl_3$, 75 MHz) δ_C 180.7 (C), 86.6 (CH), 52.5 (C), 50.0 (C), 28.9 (CH_2), 26.8 (CH_2), 18.2 (CH_3), 17.2 (CH_3), 9.5 (CH_3); **m.p.** = 155-157 °C.

1-(4-Acetylphenyl)pyrrolidin-2-one (3ab)[46]

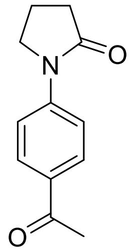

Following general procedure C, 1-(4-acetylphenyl)-5-oxopyrrolidine-3-carboxylic acid **1ab** (92.7 mg, 0.375 mmol) was used. The crude product was purified by silica gel flash column chromatography (3:7 hexane/EtOAc) to yield product **3ab** (41.7 mg, 0.206 mmol, 55%) as a white solid.

R_f 0.31 (3:7 hexane/EtOAc) **ν_{max}**/cm^{-1} 2986 (C-H), 2922 (C-H), 2897 (C-H), 1689 (C=O), 1668 (C=O); **^{1}H NMR** ($CDCl_3$, 300 MHz) δ_H 7.97 (d, *J* = 8.9 Hz, 2H), 7.75 (d, *J* = 8.9 Hz, 2H), 4.01 – 3.85 (m, 2H), 2.69 – 2.61 (m, 2H), 2.58 (s, 3H), 2.20 (m, 2H); **^{13}C NMR** ($CDCl_3$, 75 MHz) δ_C 197.0 (C), 174.7 (C), 143.5 (C), 132.7 (C), 129.4 (CH), 118.7 (CH), 48.4 (CH_2), 32.9 (CH_2), 26.5 (CH_3), 17.9 (CH_2); **m.p.** = 93-96 °C (lit.[47] 91-93 °C).

***tert*-Butyl (cyclohexylmethyl)carbamate (3ac)**[48]

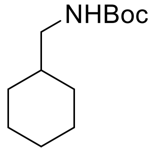

Following general procedure A, *N*-Boc tranexamic acid **1ac** (96.5 mg, 0.375 mmol) was used. The crude product was purified by silica gel flash column chromatography (9:1 hexane/EtOAc) to yield product **3aj** (40.1 mg, 0.188 mmol, 50%) as a white solid.

R_f 0.37 (9:1 hexane/EtOAc) **ν_{max}**/cm^{-1} 3346 (N-H), 2976 (C-H), 2922 (C-H), 2852 (C-H), 1692 (C=O); **^{1}H NMR** ($CDCl_3$, 300 MHz) δ_H 4.55 (br s, 1H), 2.95 (t, *J* = 6.5 Hz, 2H), 1.78 – 1.60 (m, 5H),

1.44 (s, 9H), 1.32 – 1.06 (m, 4H), 0.99 – 0.81 (m, 2H); **^{13}C NMR** ($CDCl_3$, 75 MHz) δ_C 156.1 (C), 78.9 (C), 46.9 (CH_2), 38.3 (CH), 30.7 (CH_2), 28.4 (CH_3), 26.4 (CH_2), 25.8 (CH_2); **m.p.** = 58-60 °C.

2,4-Dichloro-1-ethoxybenzene (3ad)[49]

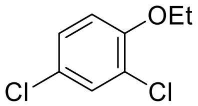

Following general procedure B, dichloroprop **1ad** (82.1 mg, 0.375 mmol) was used. The crude product was purified by silica gel flash column chromatography (pentane→99:1 pentane/DCM) to yield product **3ad** (32.2 mg, 0.184 mmol, 49%) as a colourless oil.

R_f 0.39 (99:1 pentane/DCM) **ν_{max}**/cm^{-1} 3073 (C-H Ar), 3026 (C-H Ar), 2983 (C-H), 2926 (C-H), 1487 (C=C Ar), 1472 (C=C Ar), 1442 (C=C Ar), 1255 (C-O-C), 1104 (C-O-C); **^{1}H NMR** ($CDCl_3$, 300 MHz) δ_H 7.36 (d, *J* = 2.6 Hz, 1H), 7.17 (dd, *J* = 8.8, 2.6 Hz, 1H), 6.83 (d, *J* = 8.8 Hz, 1H), 4.08 (q, *J* = 7.0 Hz, 2H), 1.46 (t, *J* = 7.0 Hz, 3H); **^{13}C NMR** ($CDCl_3$, 75 MHz) δ_C 153.3 (C), 129.9 (CH), 127.5 (CH), 125.5 (C), 123.7 (C), 114.0 (CH), 65.1 (CH_2), 14.6 (CH_3).

NMR Spectra

ebmh552hccha.1.fid
1H 300.1MHz Job 96515 McLean Euan B 552HCCHA CDCl3 25.2�C
proton characterisation

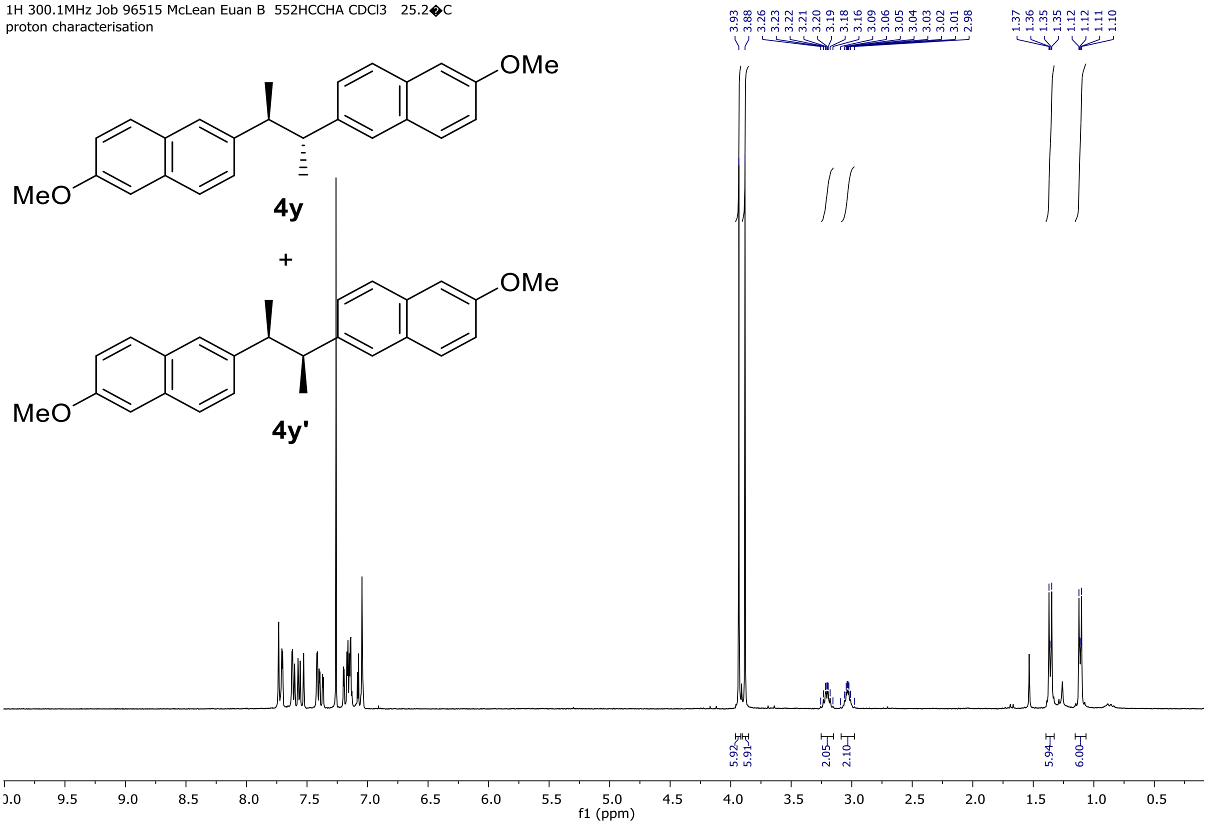

ebmc552hccha.1.fid
13C 75.5MHz Job 96526 McLean Euan B 552HCCHA CDCl3 25.0�C 3 hours 1 min
carbon characterisation

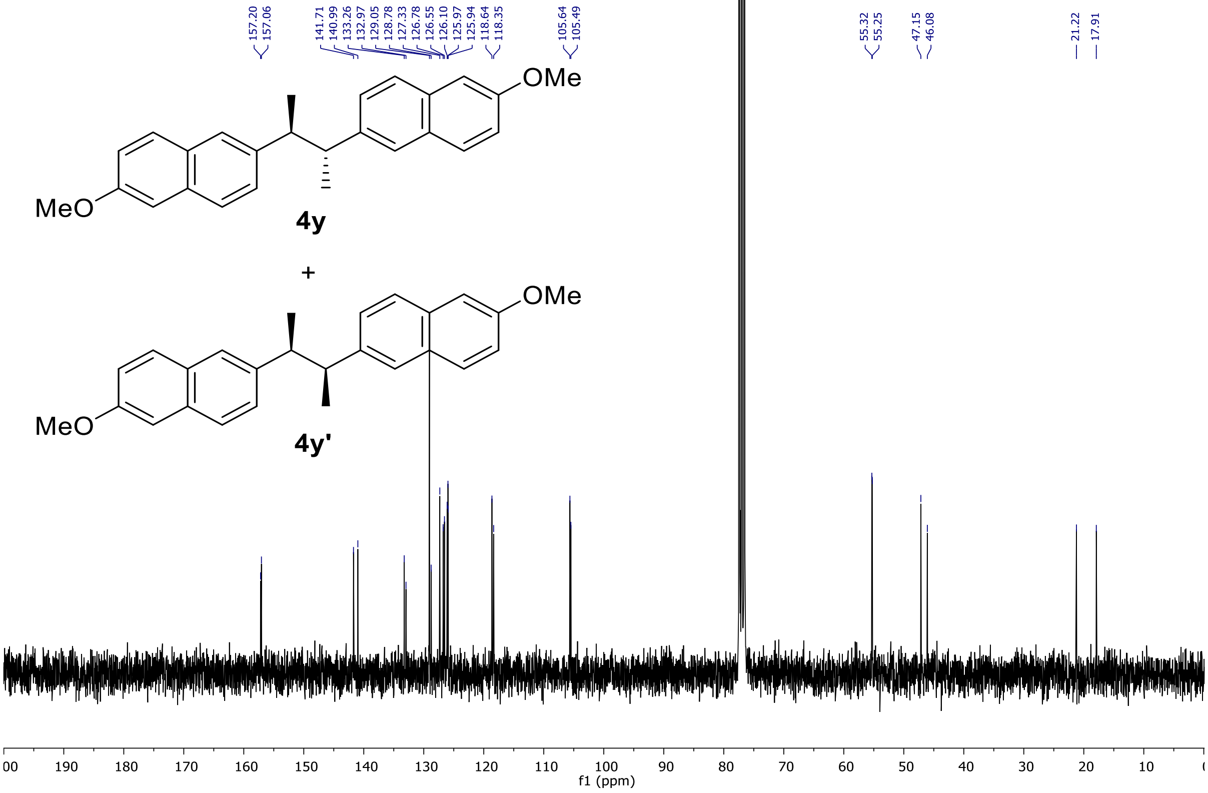

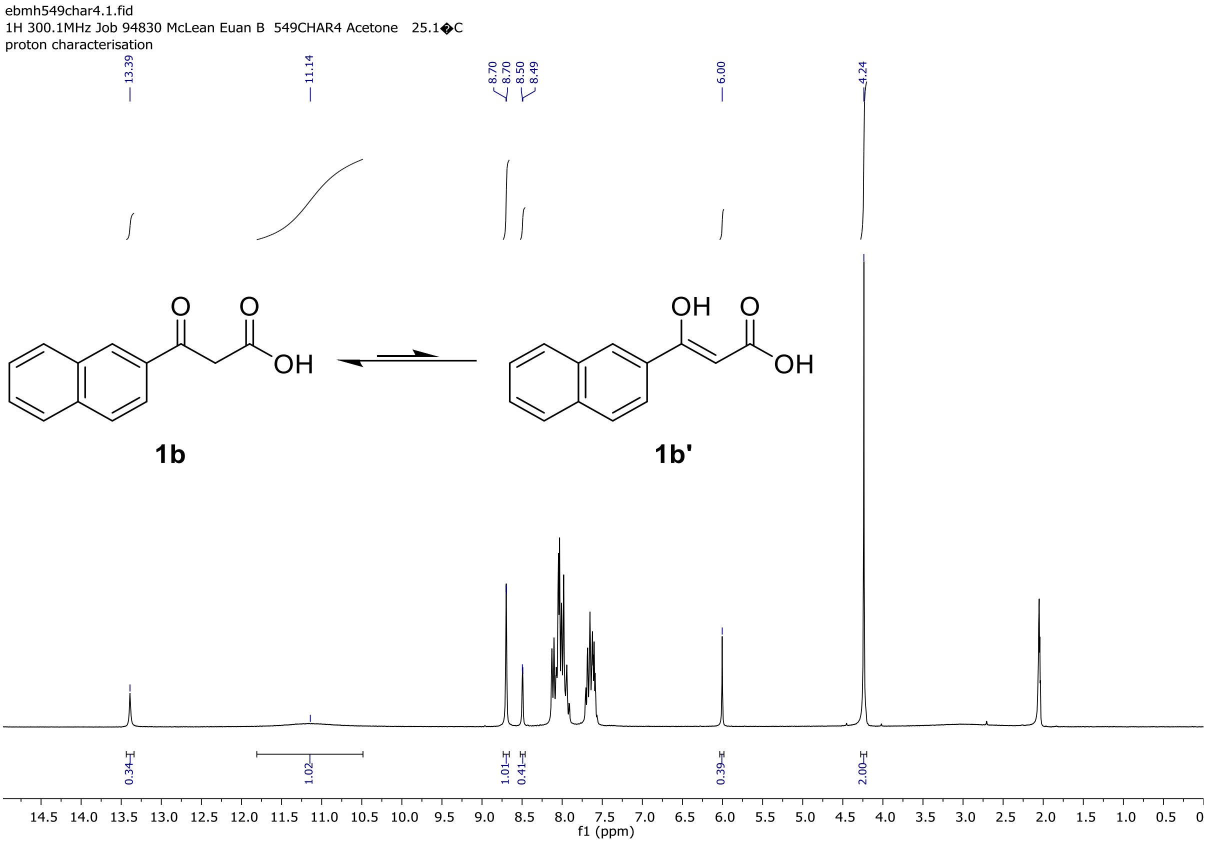
ebmh549char4.1.fid
1H 300.1MHz Job 94830 McLean Euan B 549CHAR4 Acetone 25.1�C
proton characterisation
13.39
11.14
8.70
8.70
8.50
8.49
6.00
4.24
1b
1b'
0.34
1.02
1.01
0.41
0.39
2.00
f1 (ppm)

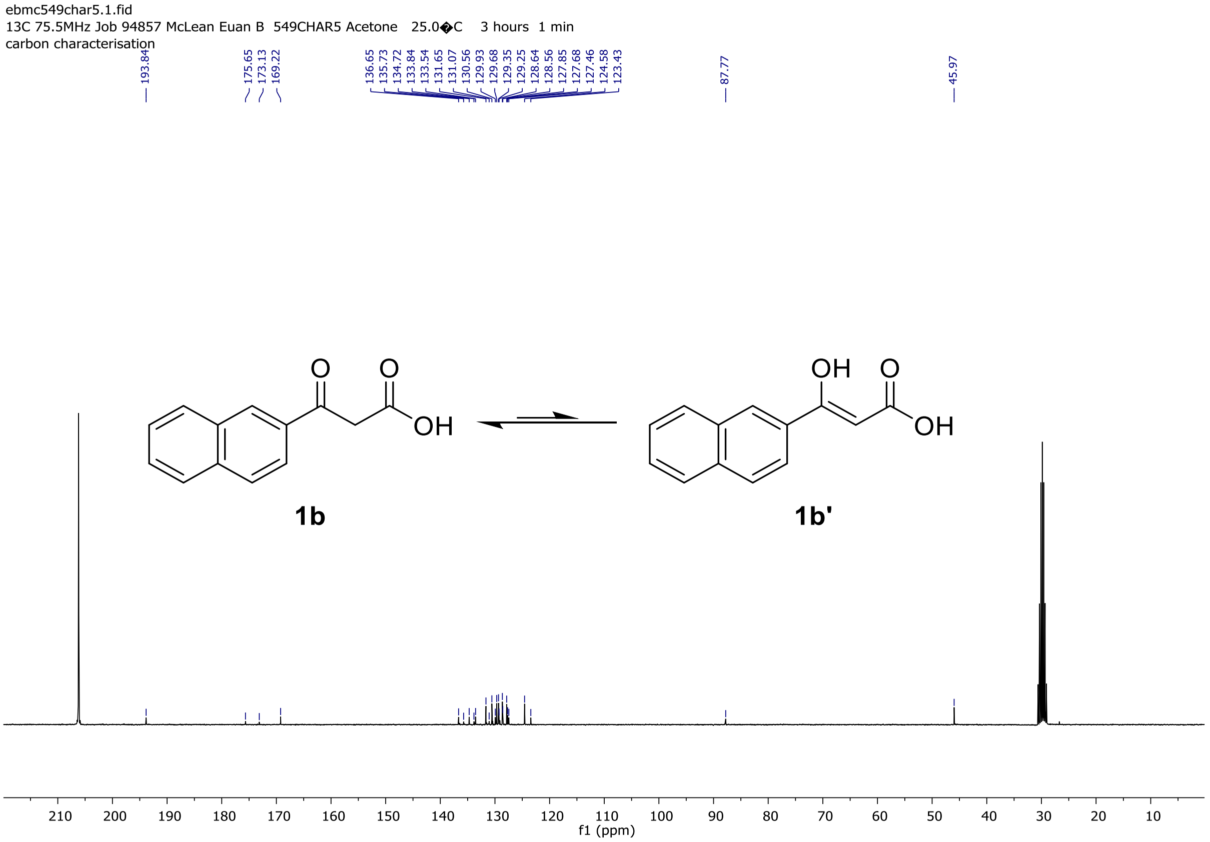
ebmc549char5.1.fid
13C 75.5MHz Job 94857 McLean Euan B 549CHAR5 Acetone 25.0�C 3 hours 1 min
carbon characterisation
193.84
175.65
173.13
169.22
136.65
135.73
134.72
133.84
133.54
131.65
131.07
130.56
129.93
129.68
129.35
129.25
128.64
128.56
127.85
127.68
127.46
124.58
123.43
87.77
45.97
1b
1b'
f1 (ppm)

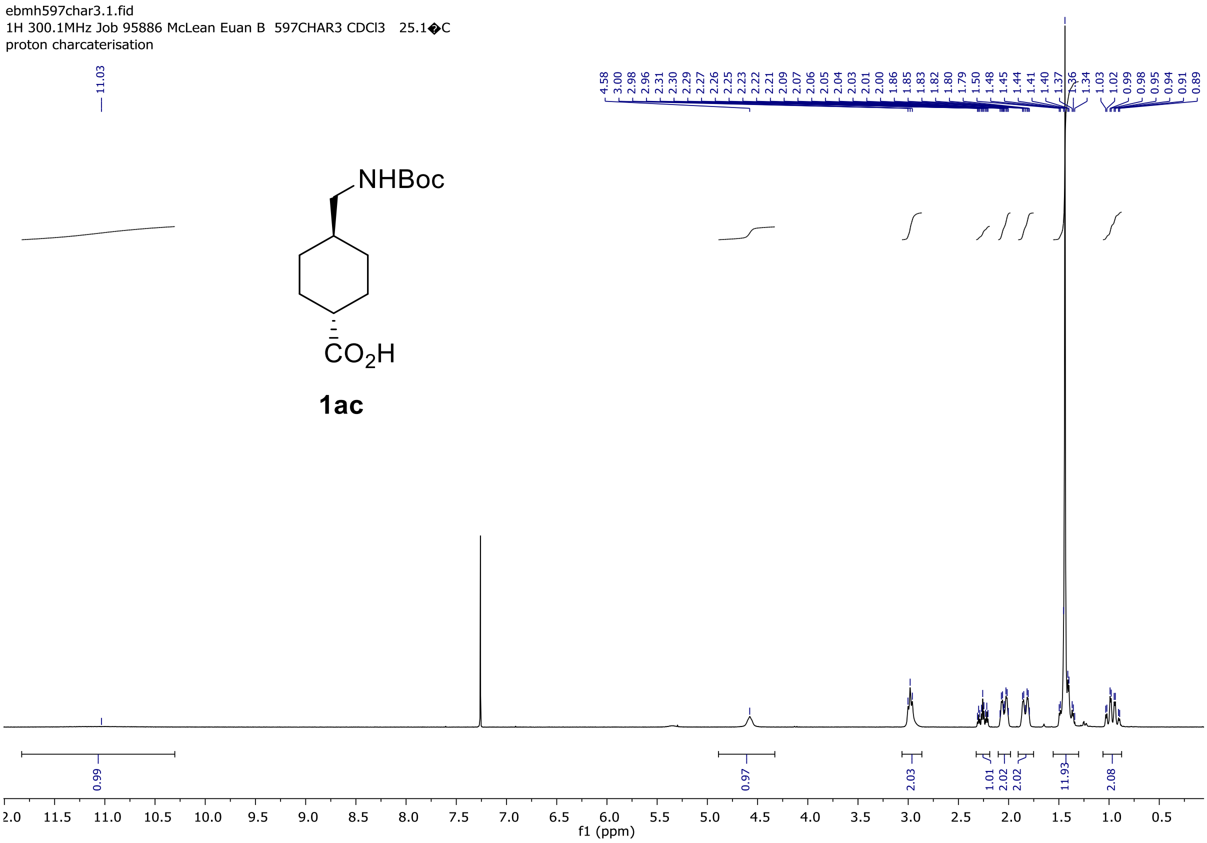
ebmh597char3.1.fid
1H 300.1MHz Job 95886 McLean Euan B 597CHAR3 CDCl3 25.1�C
proton charcaterisation
NHBoc
CO_2H
1ac
f1 (ppm)

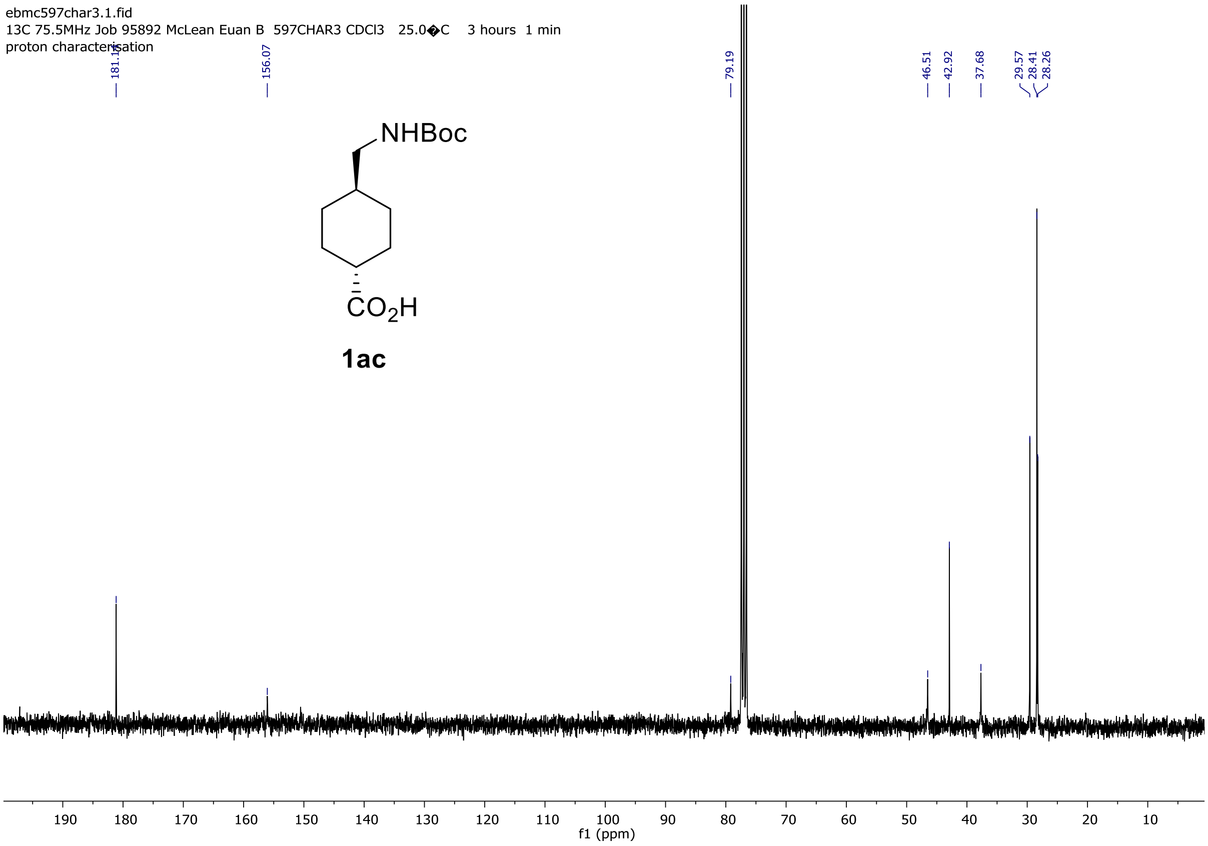
ebmc597char3.1.fid
13C 75.5MHz Job 95892 McLean Euan B 597CHAR3 CDCl3 25.0�C 3 hours 1 min
proton characterisation
NHBoc
CO_2H
1ac
f1 (ppm)

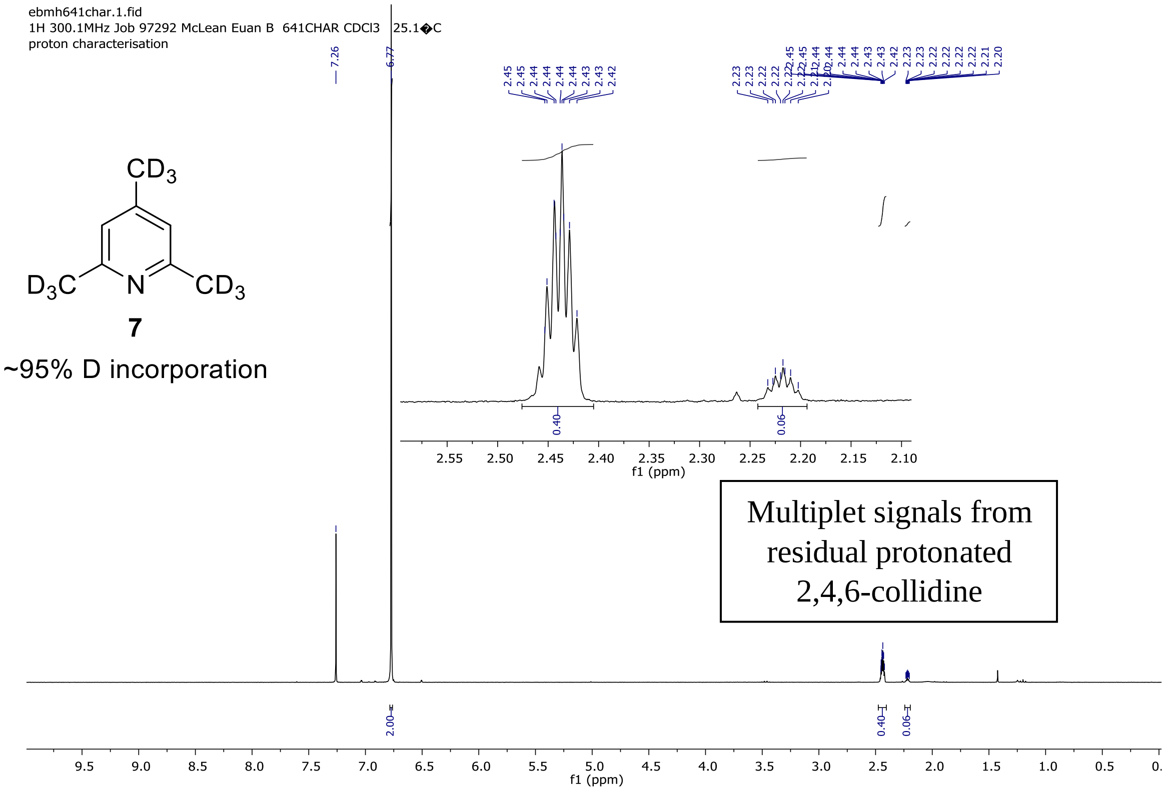
ebmh641char.1.fid
1H 300.1MHz Job 97292 McLean Euan B 641CHAR CDCl3 25.1�C
proton characterisation
7.26
6.77
CD_3
D_3C
N
CD_3
7
~95% D incorporation
0.40
0.06
f1 (ppm)
Multiplet signals from residual protonated 2,4,6-collidine
2.00
0.40
0.06
f1 (ppm)

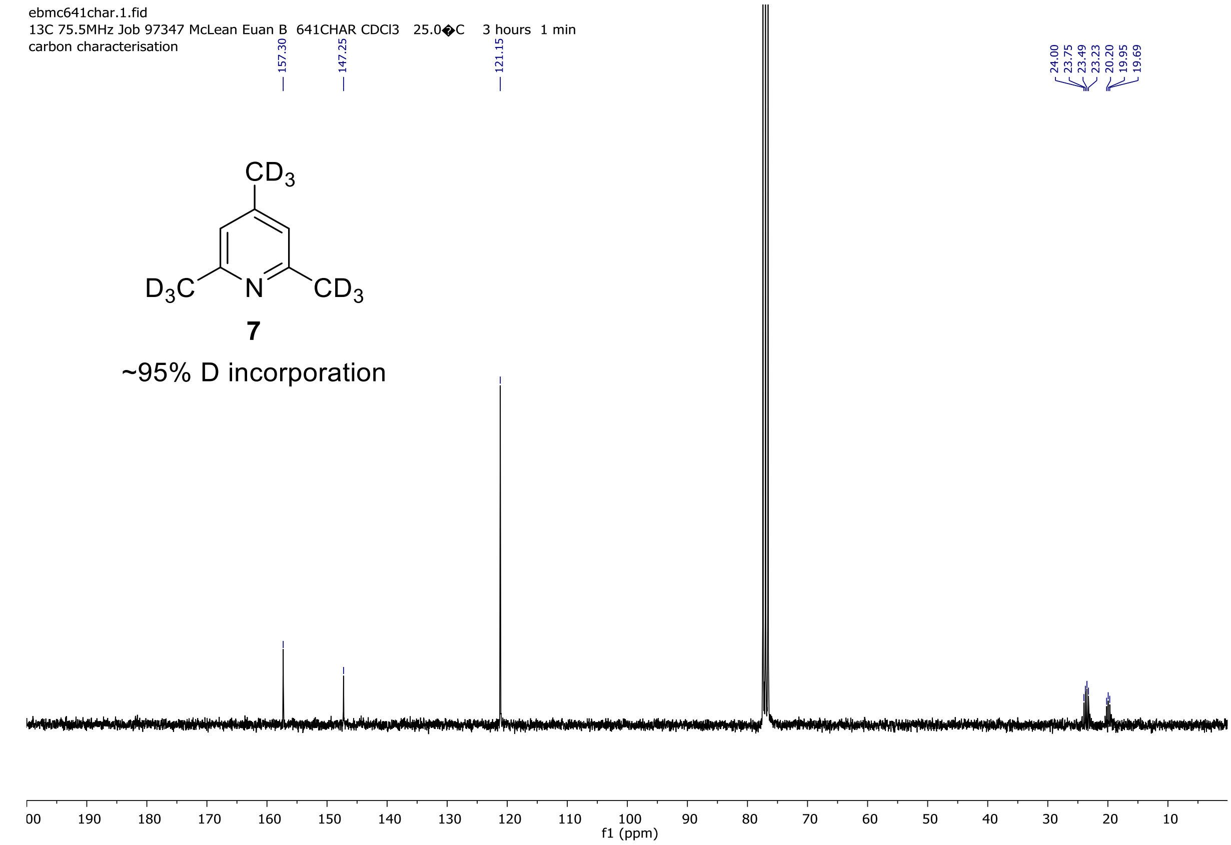
ebmc641char.1.fid
13C 75.5MHz Job 97347 McLean Euan B 641CHAR CDCl3 25.0�C 3 hours 1 min
carbon characterisation
157.30
147.25
121.15
24.00
23.75
23.49
23.23
20.20
19.95
19.69
CD_3
D_3C
N
CD_3
7
~95% D incorporation
f1 (ppm)

ebmh406bchar.1.fid
1H 300.1MHz Job 90907 McLean Euan B 406BCHAR CDCl3 25.1�C
proton characterisation

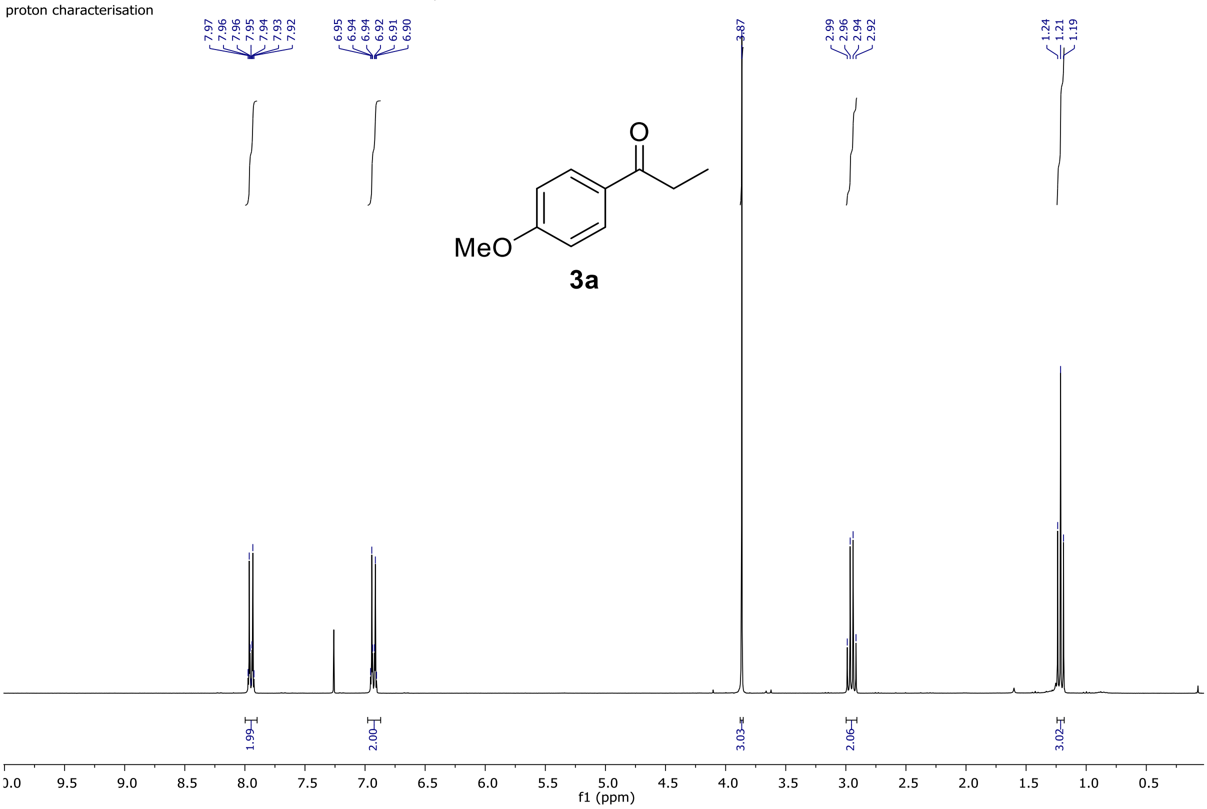

ebmc406bchar.1.fid
13C 75.5MHz Job 90958 McLean Euan B 406BCHAR CDCl3 25.0�C 2 hours 43 min
carbon characterisation

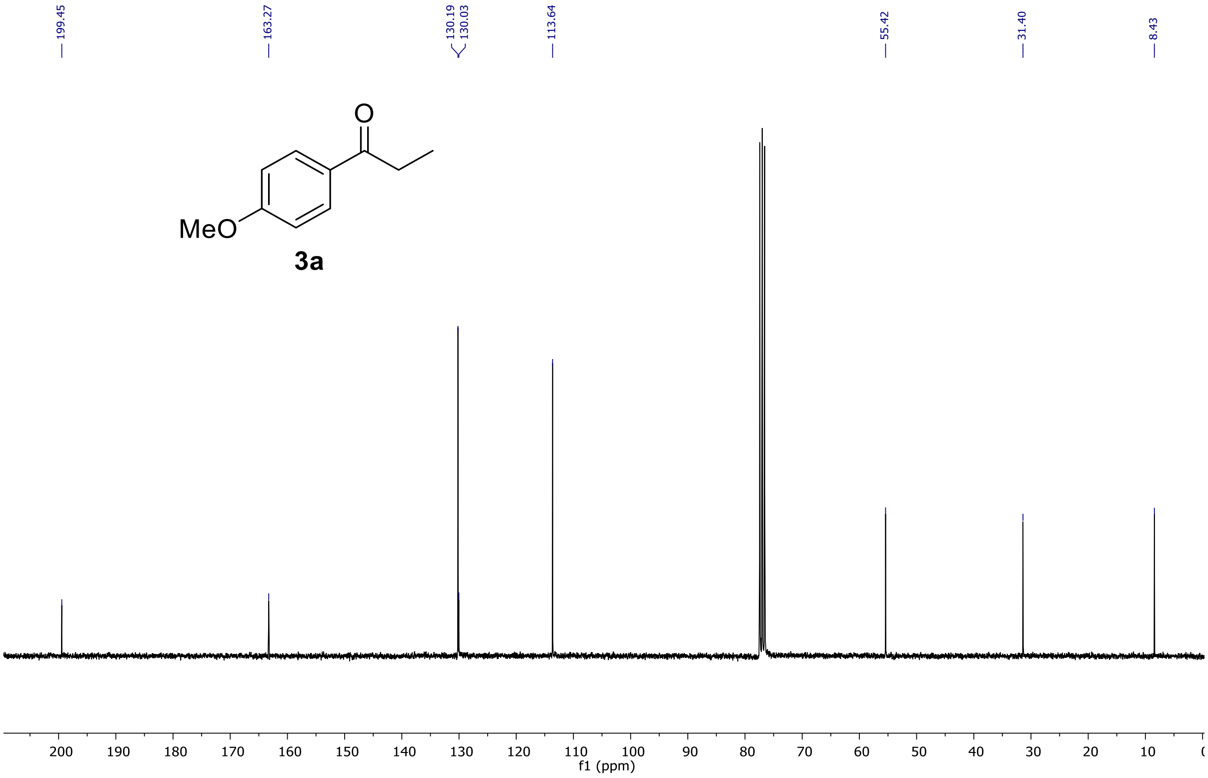

ebmh553char.1.fid
1H 300.1MHz Job 94797 McLean Euan B 553CHAR CDCl3 25.5�C
proton characterisation

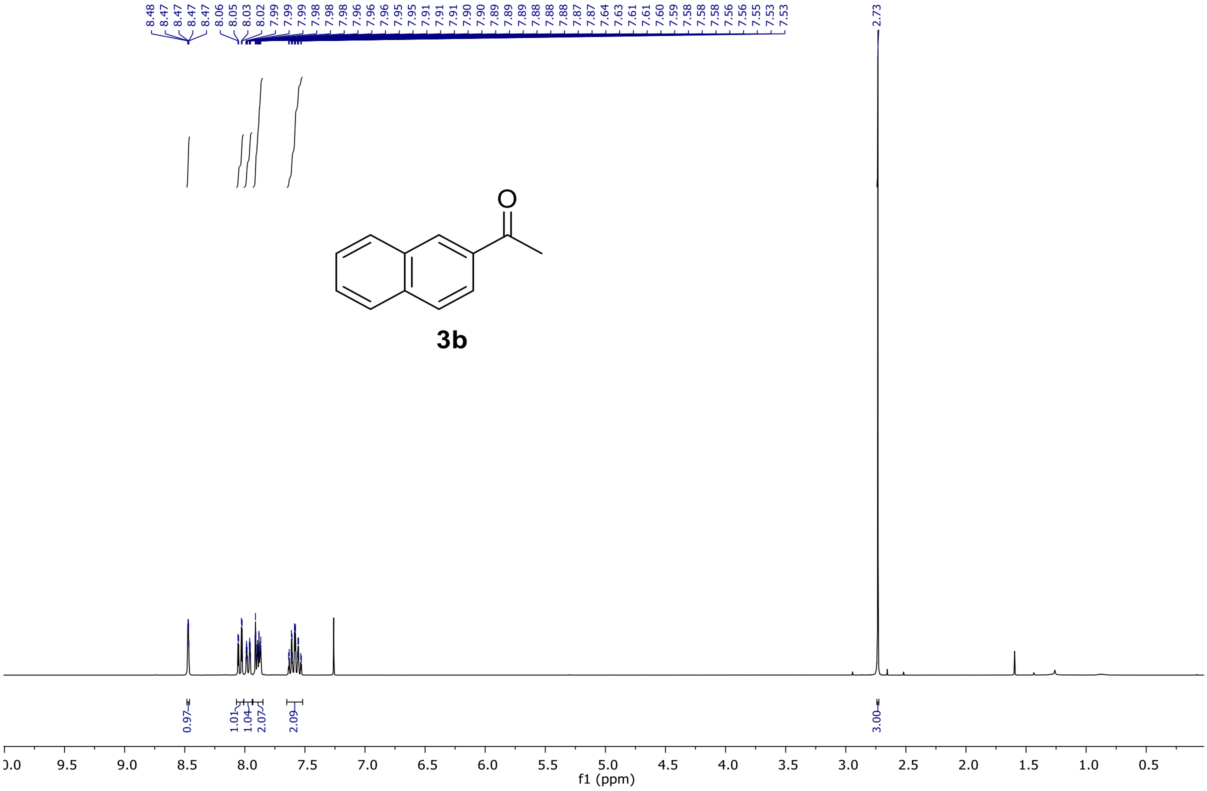

ebmc553char.1.fid
13C 75.5MHz Job 94809 McLean Euan B 553CHAR CDCl3 25.1�C 3 hours 1 min
carbon characterisation

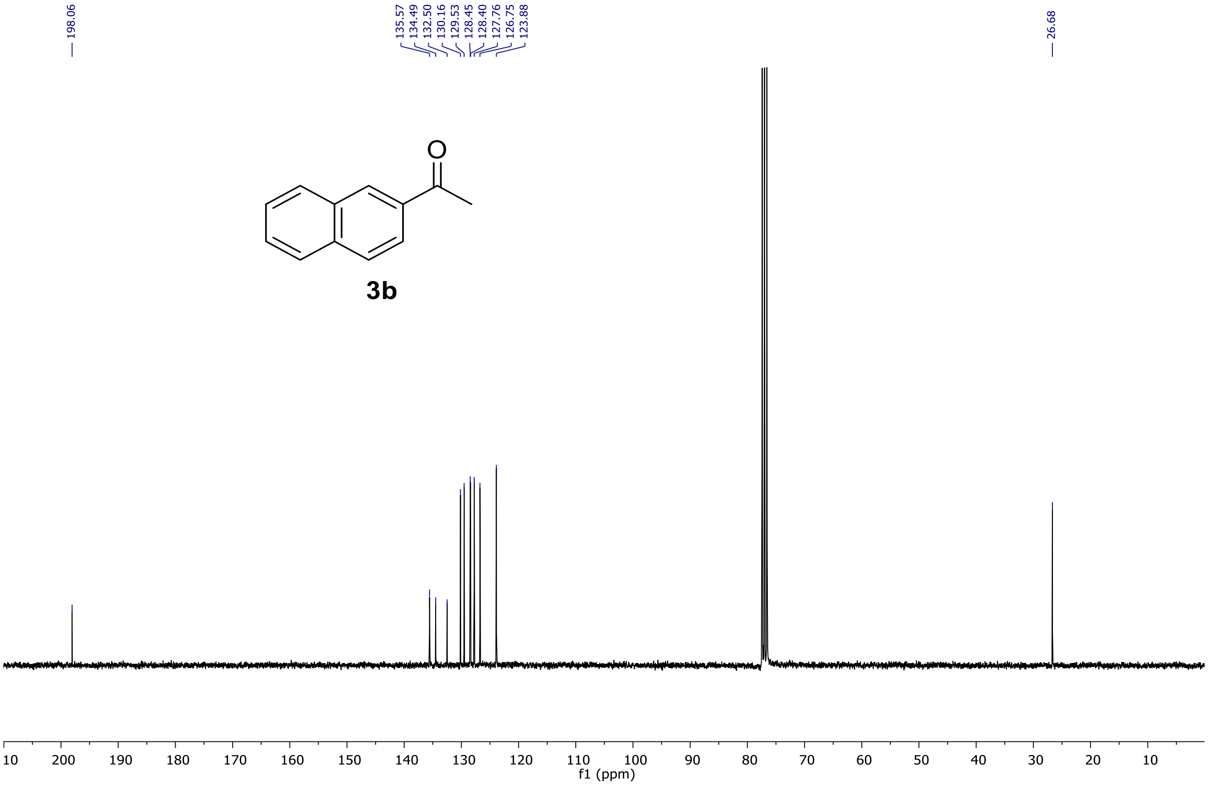

ebmh476char.1.fid
1H 400.1MHz Job 41563 McLean Euan B 476CHAR CDCl3 24.8�C
proton characterisation

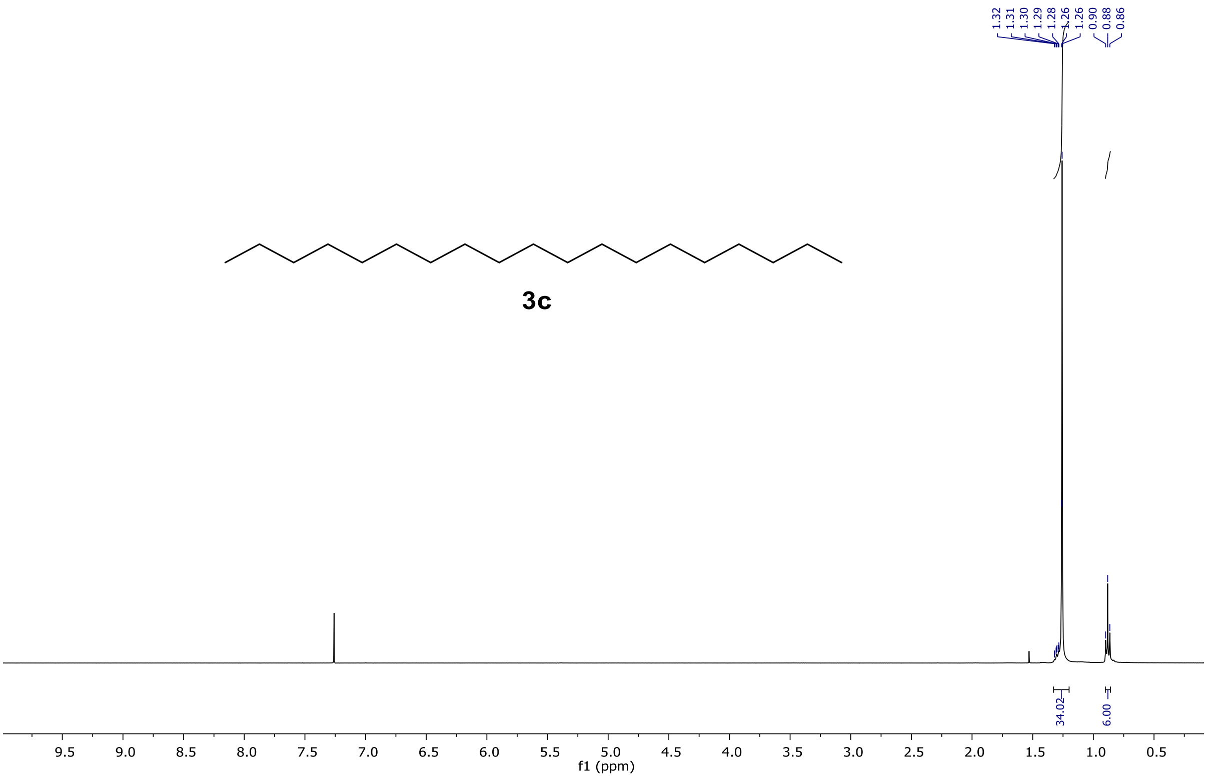

ebmc476char.1.fid
13C 100.6MHz Job 41576 McLean Euan B 476CHAR CDCl3 26.5�C 0 hour 29 min
carbon characterisation

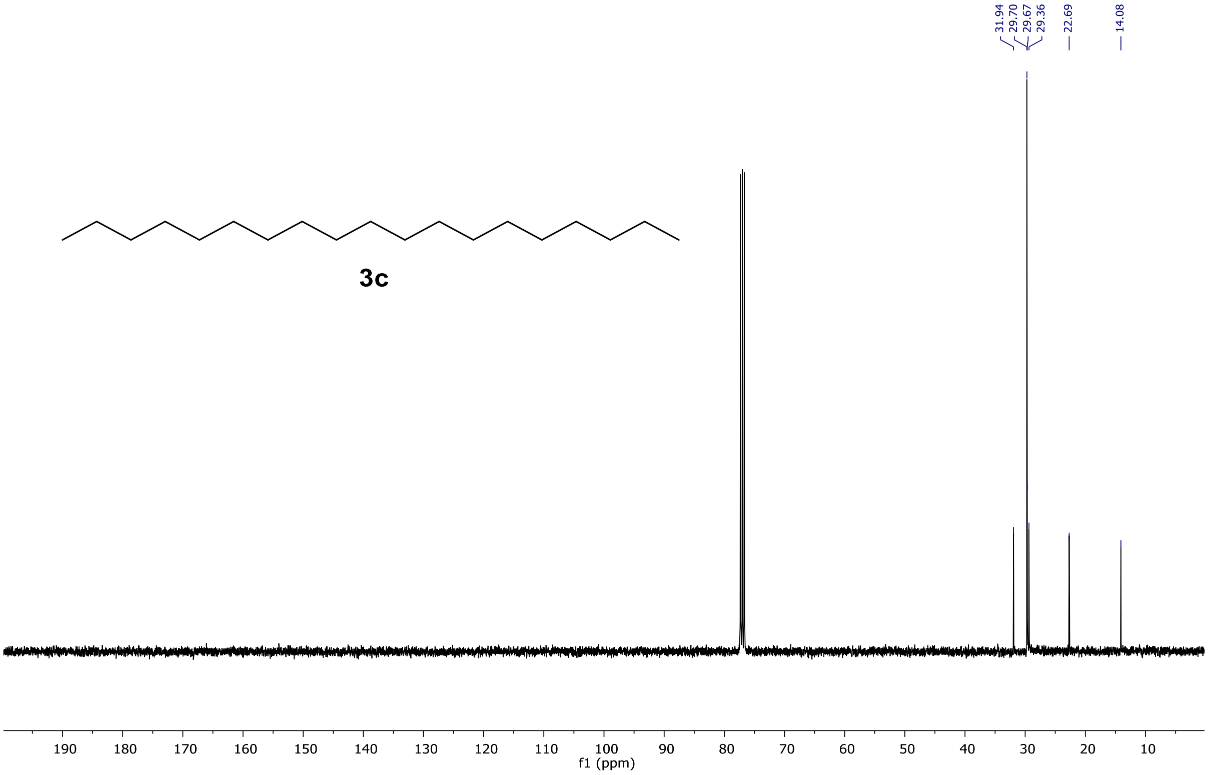

ebmh477char2.1.fid
1H 300.1MHz Job 93205 McLean Euan B 477CHAR2 CDCl3 25.0�C
proton charcaterisation

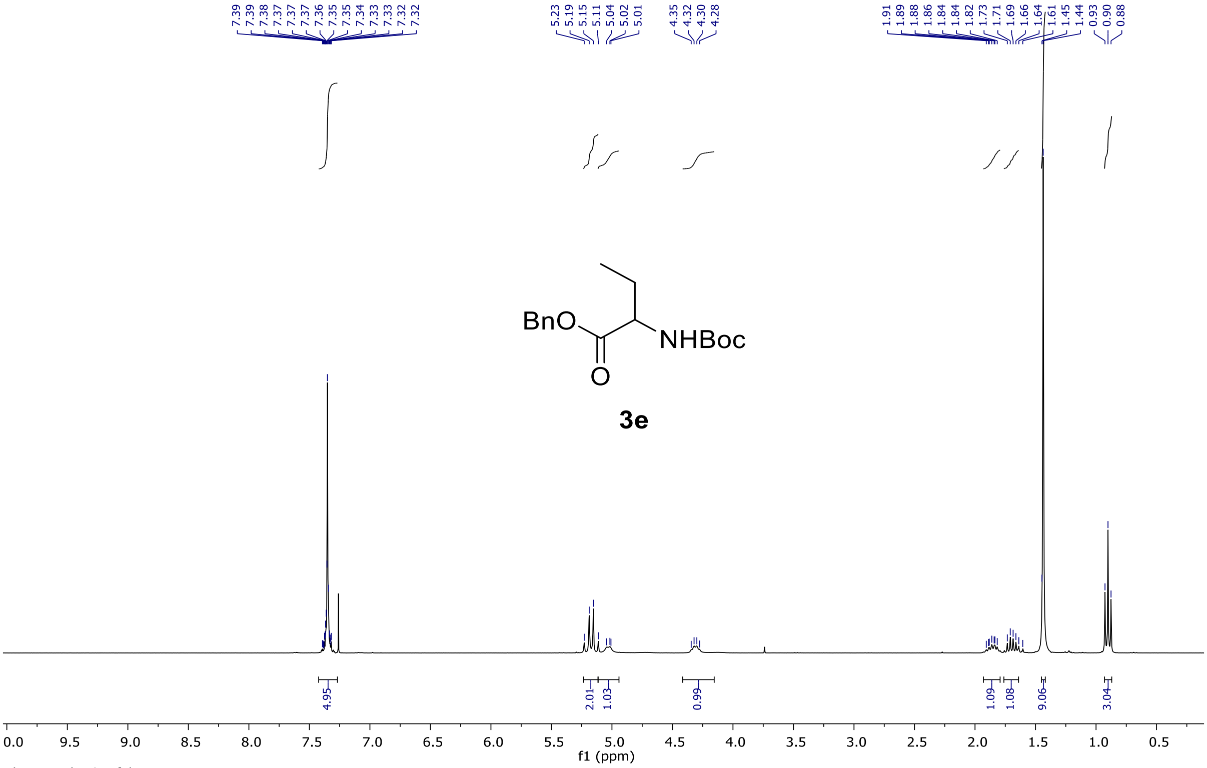

ebmc477char2.1.fid
13C 75.5MHz Job 93226 McLean Euan B 477CHAR2 CDCl3 25.1�C 3 hours 1 min
carbon characterisation

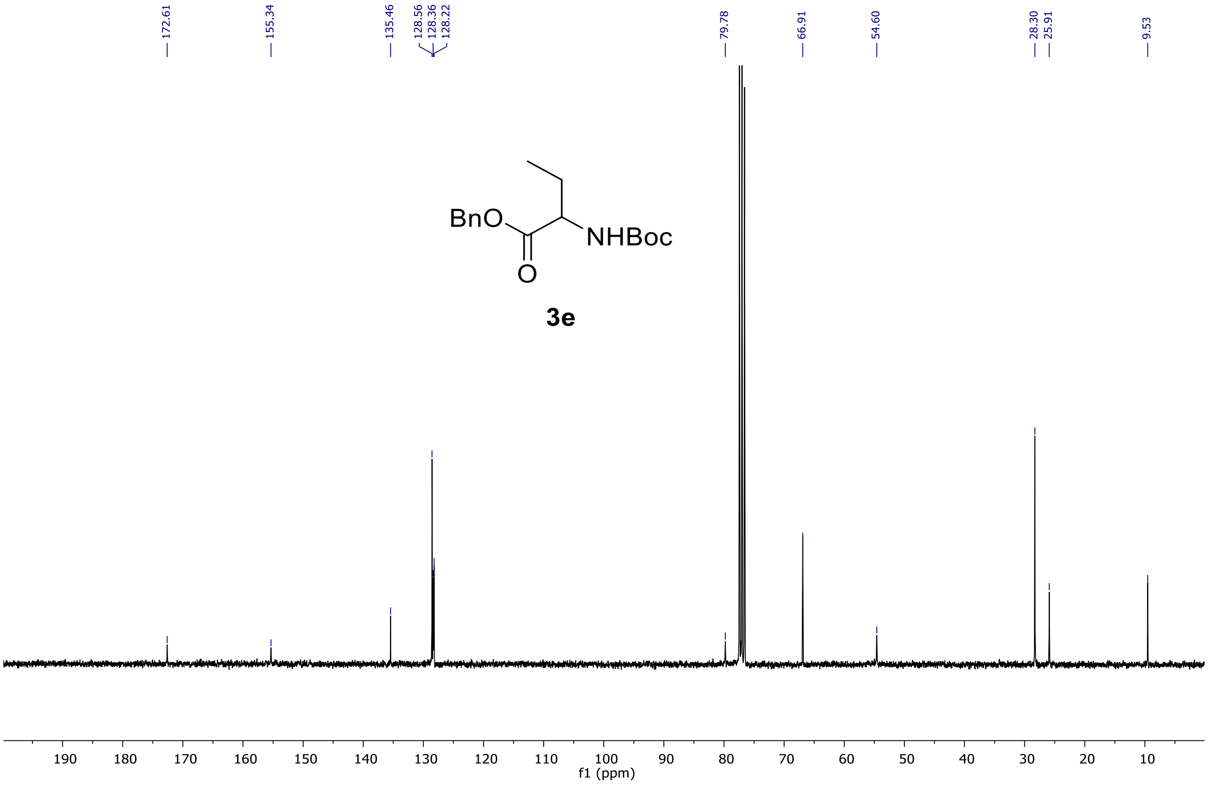

ebmh532char.1.fid
1H 300.1MHz Job 94401 McLean Euan B 532CHAR CDCl3 25.2�C
proton characterisation

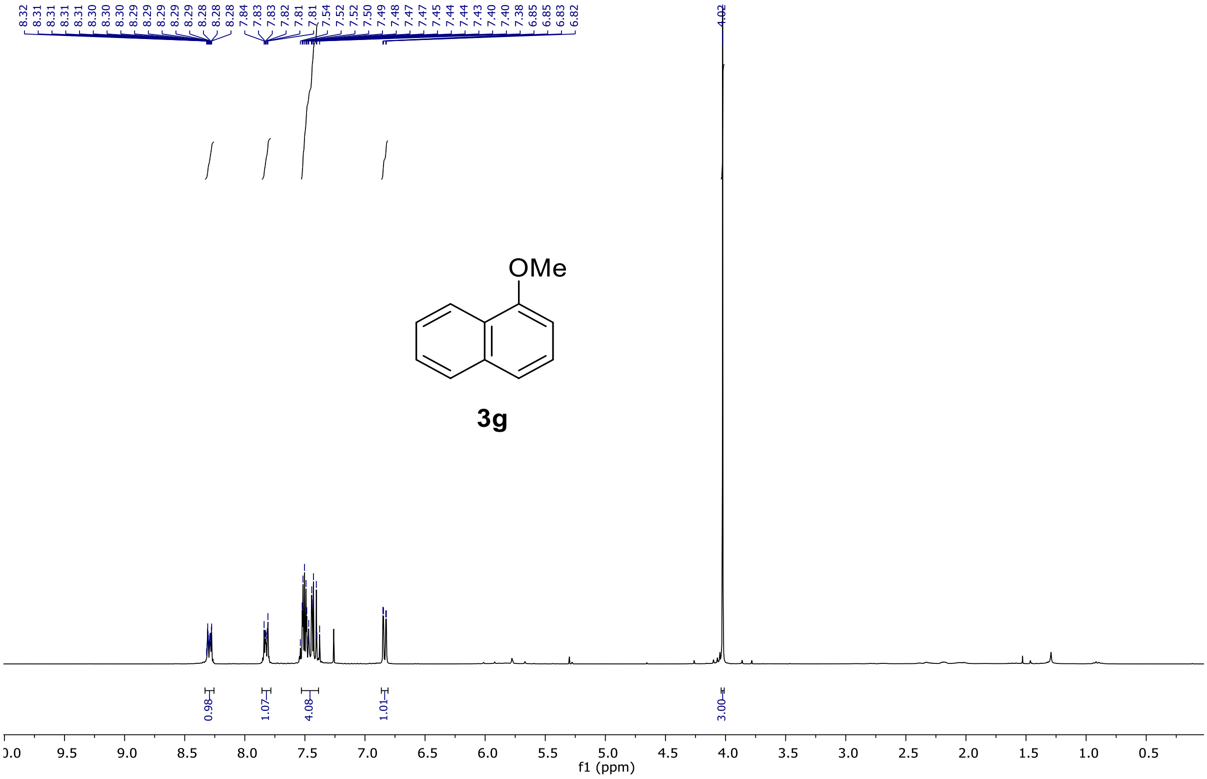

ebmc532char.1.fid
13C 75.5MHz Job 94416 McLean Euan B 532CHAR CDCl3 25.0�C 3 hours 1 min
carbon characterisation

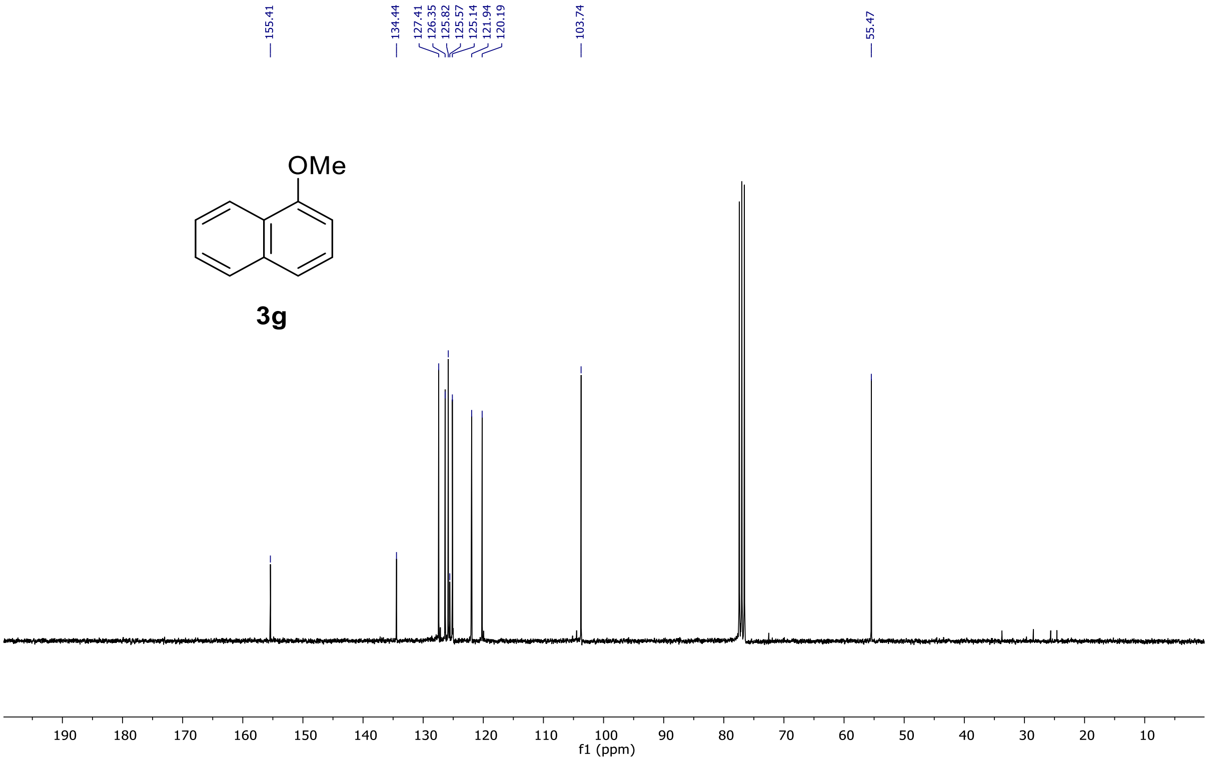

ebmh426char.1.fid
1H 300.1MHz Job 92202 McLean Euan B 426CHAR CDCl3 24.9�C
proton characterisation

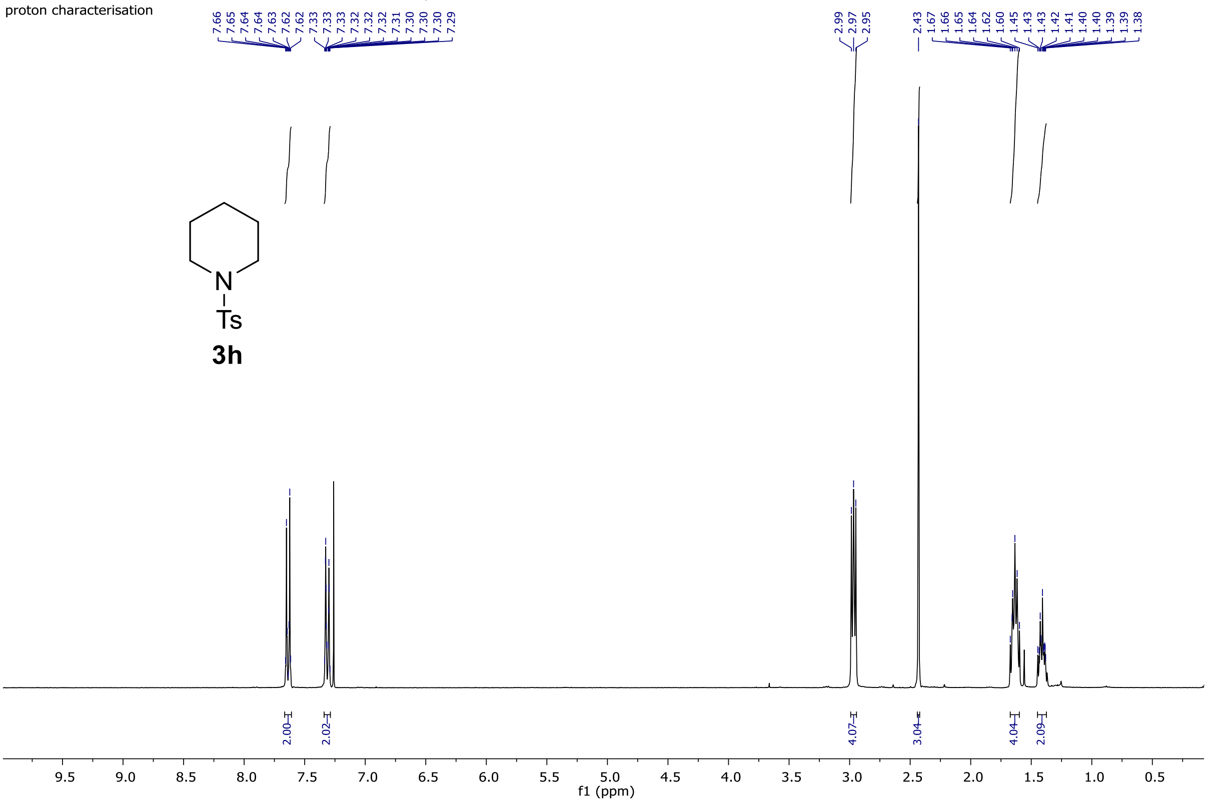

ebmc426char.1.fid
13C 75.5MHz Job 92247 McLean Euan B 426CHAR CDCl3 25.0�C 3 hours 1 min
carbon characterisation

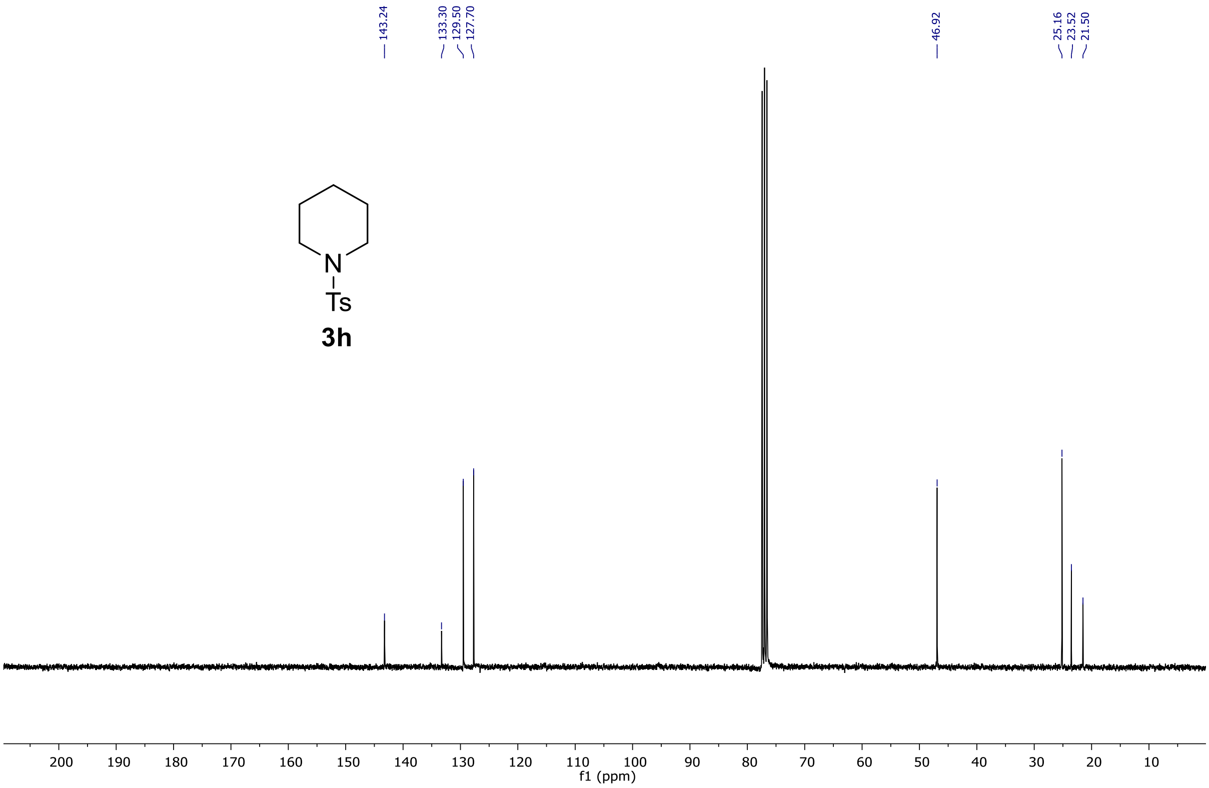

ebmh425char.1.fid
1H 300.1MHz Job 92480 McLean Euan B 425CHAR CDCl3 25.0�C
proton characterisation

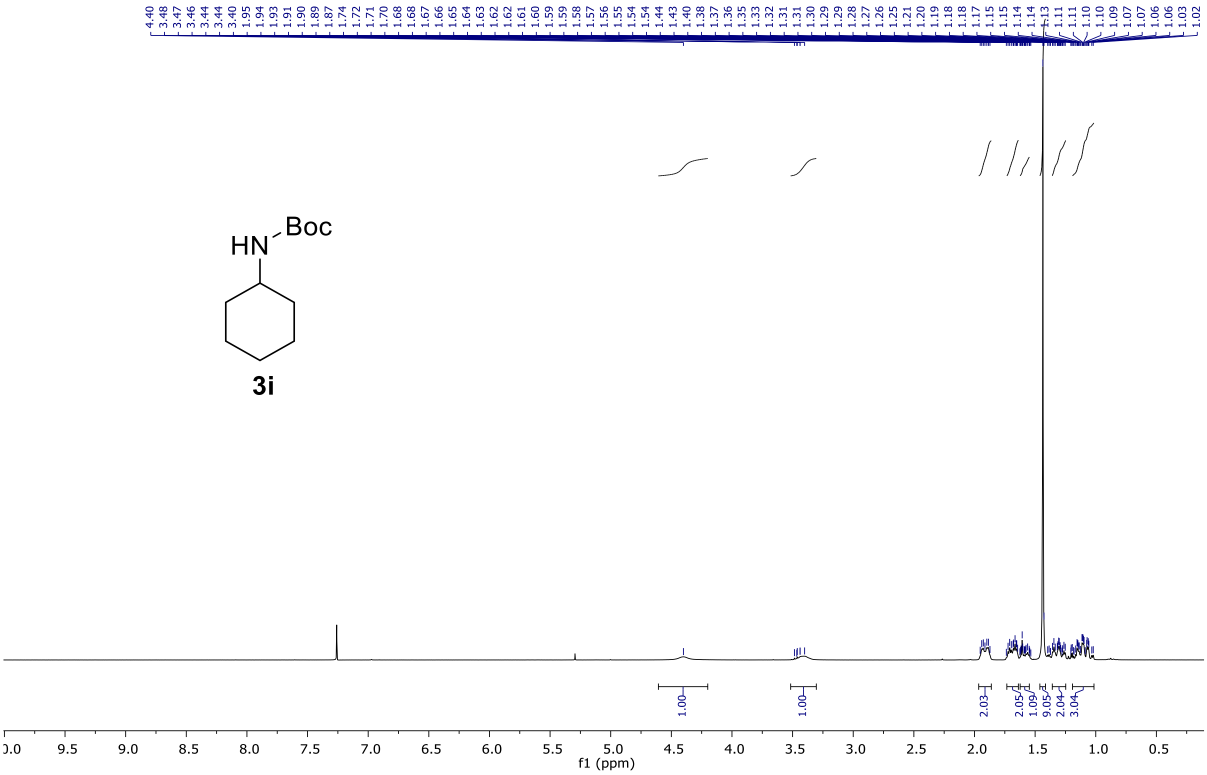

ebmc425char.1.fid
13C 75.5MHz Job 92486 McLean Euan B 425CHAR CDCl3 25.5�C 1 hour 12 min
carbon characterisation

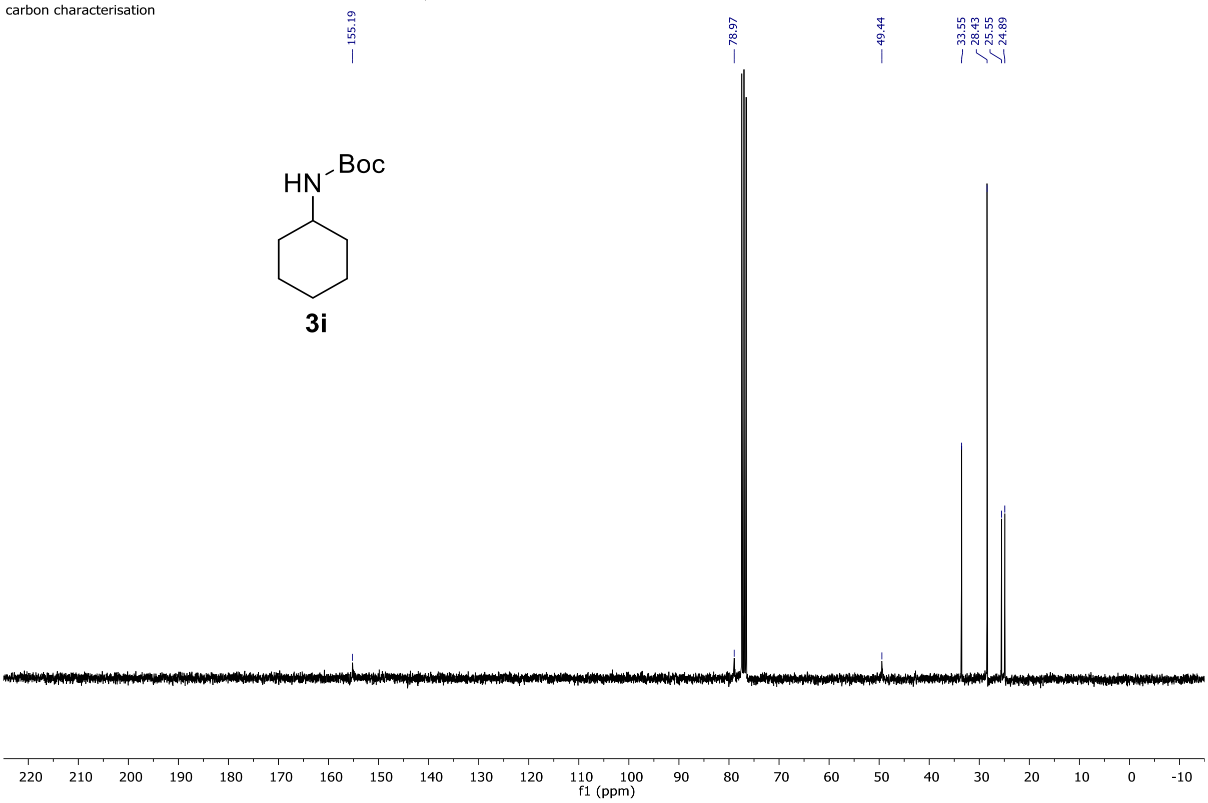

ebmh424char5.1.fid
1H 300.1MHz Job 92257 McLean Euan B 424CHAR5 CDCl3 25.0�C
proton characterisation

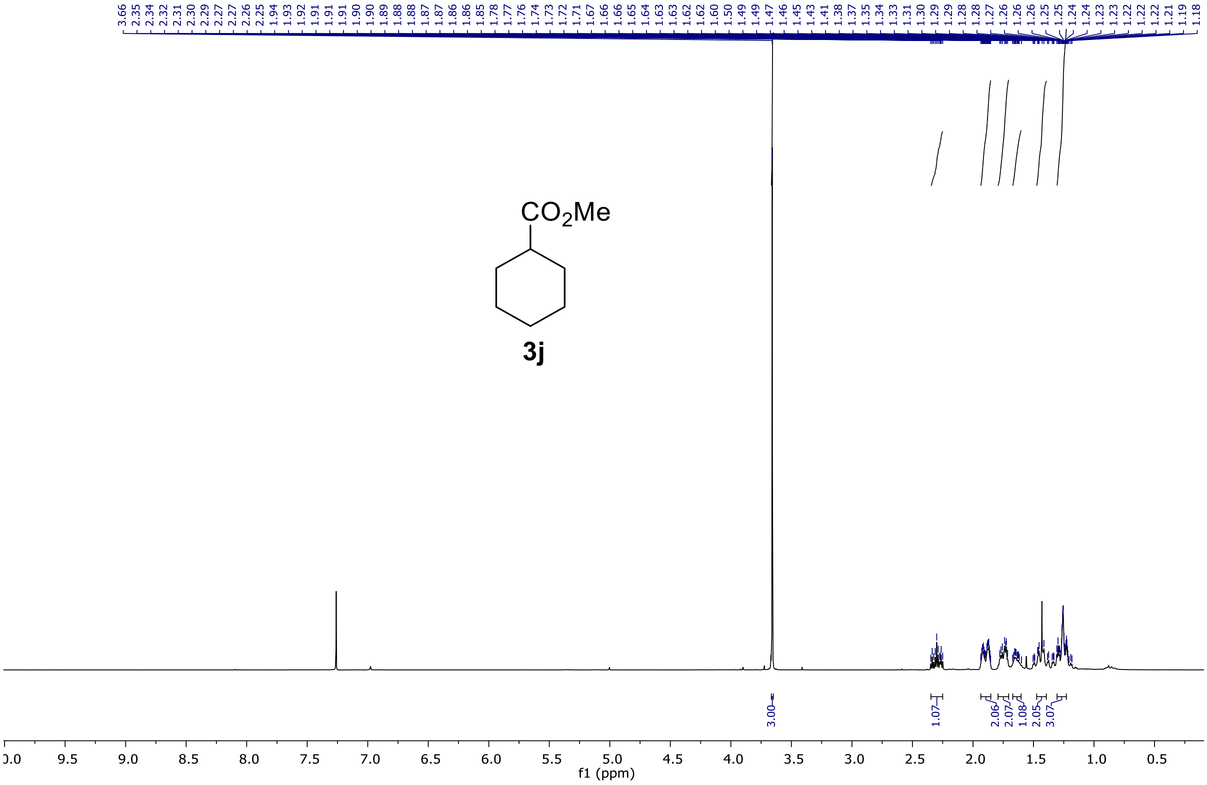

ebmc424char5.1.fid
13C 75.5MHz Job 92312 McLean Euan B 424CHAR5 CDCl3 24.9�C 3 hours 1 min
carbon characterisation

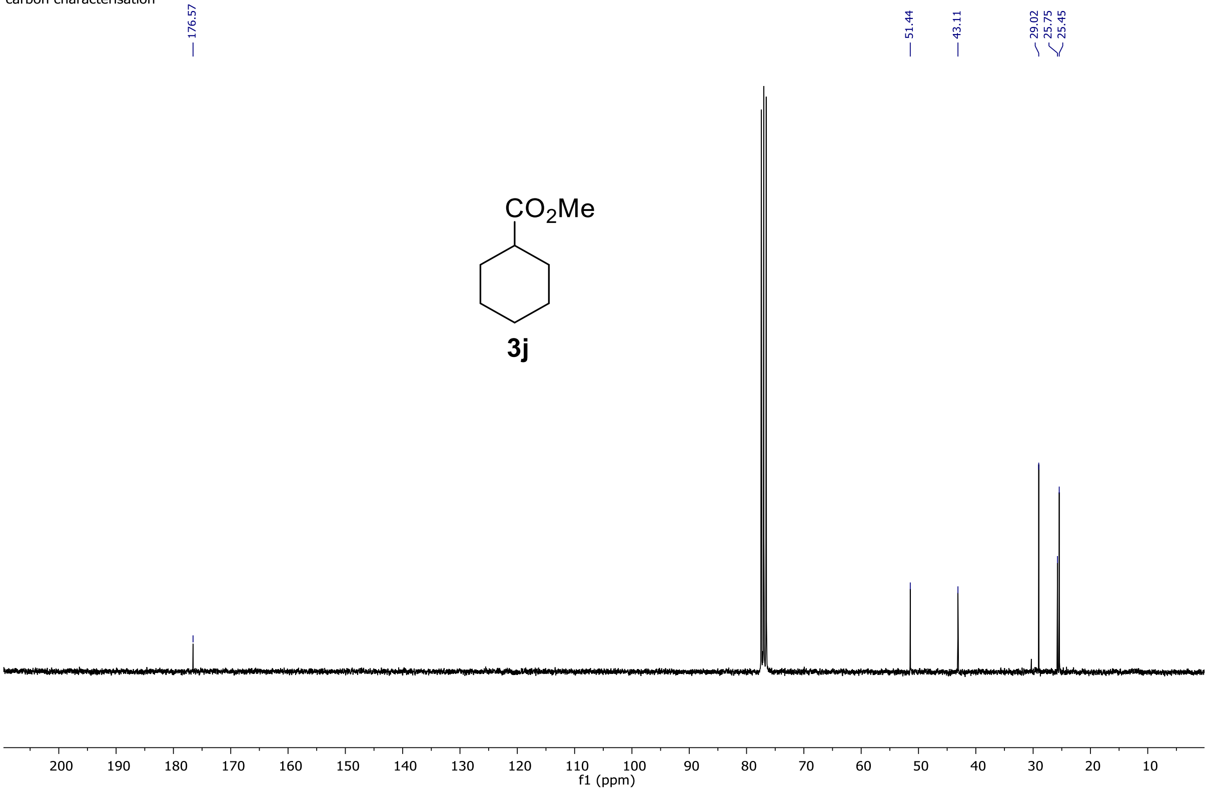

ebmh427char.1.fid
1H 300.1MHz Job 92481 McLean Euan B 427CHAR CDCl3 25.1�C
proton characterisation

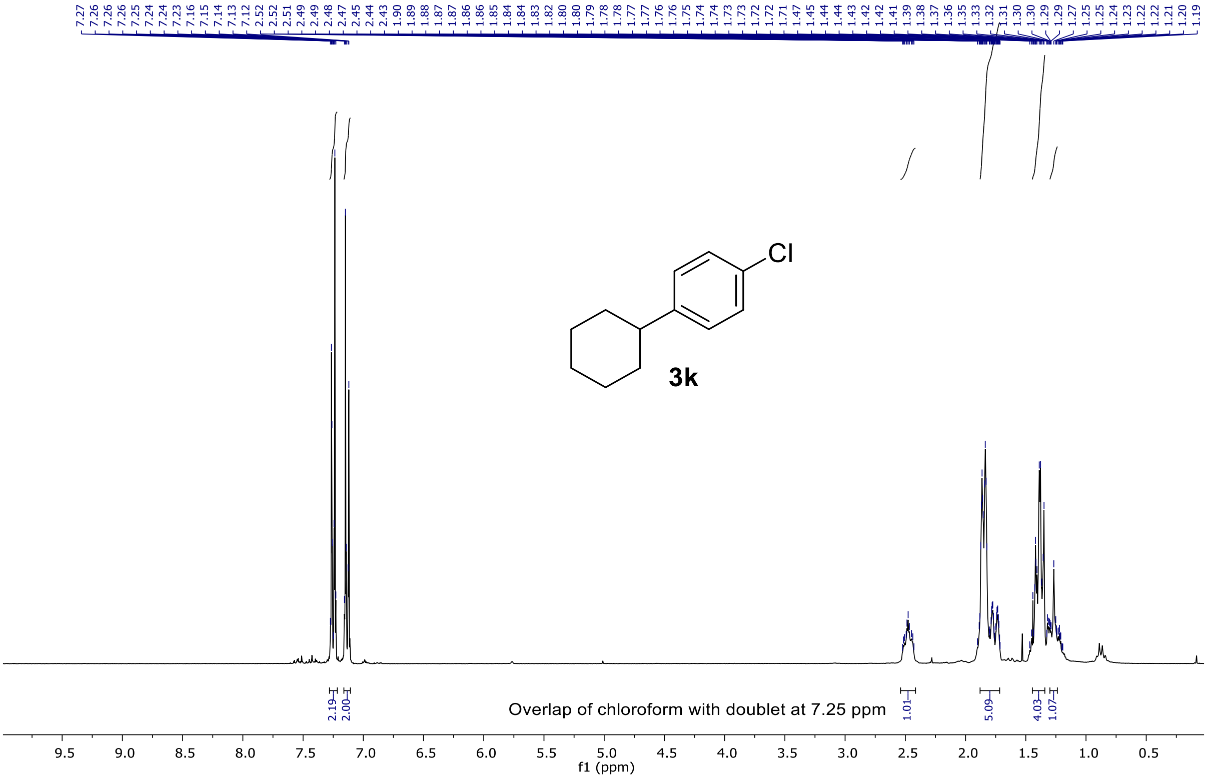

ebmc427char.1.fid
13C 75.5MHz Job 92487 McLean Euan B 427CHAR CDCl3 25.2�C 1 hour 12 min
carbon characterisation

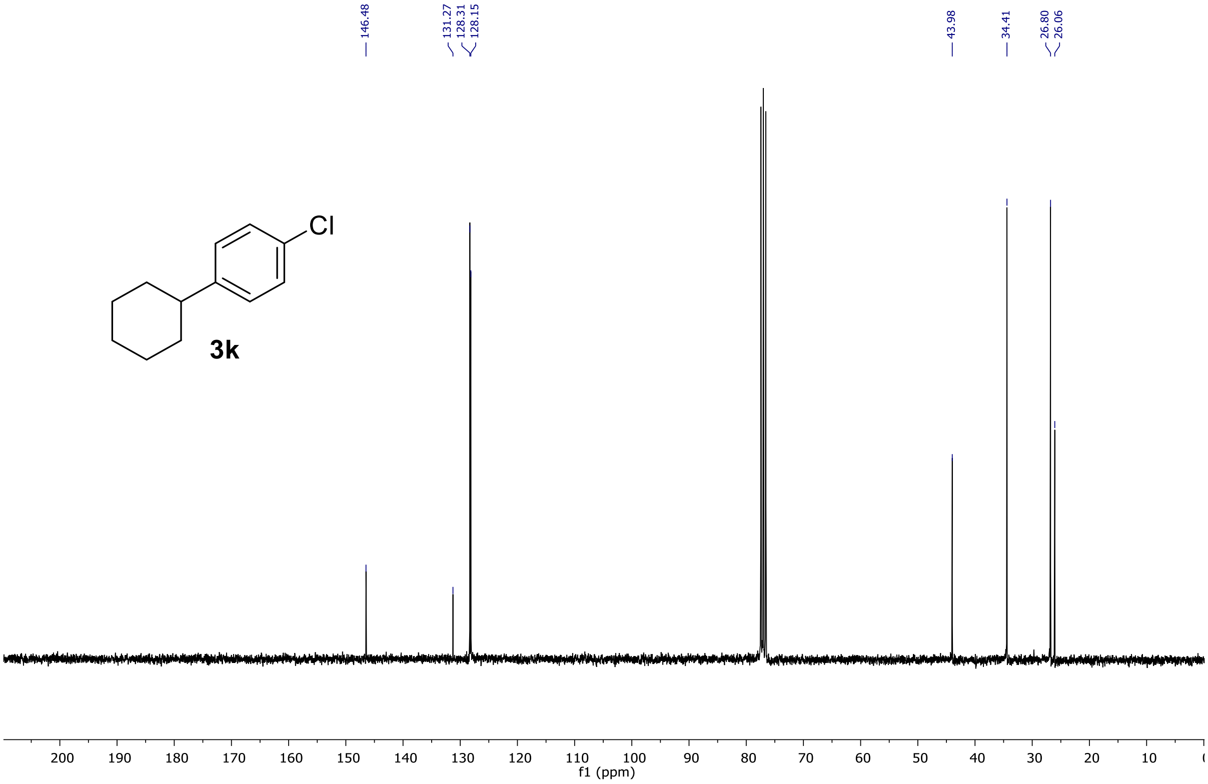

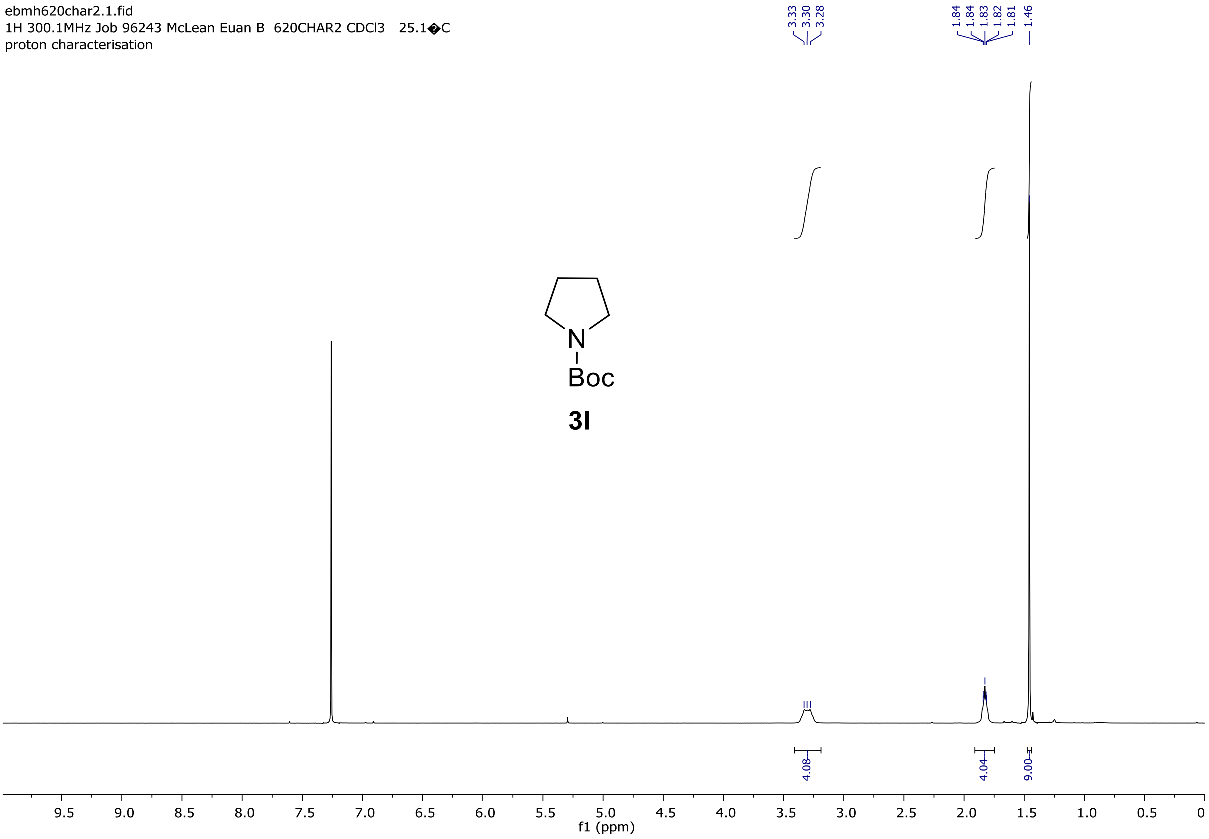
ebmh620char2.1.fid
1H 300.1MHz Job 96243 McLean Euan B 620CHAR2 CDCl3 25.1�C
proton characterisation
3.33
3.30
3.28
1.84
1.84
1.83
1.82
1.81
1.46
N
Boc
3l
4.08
4.04
9.00
9.5 9.0 8.5 8.0 7.5 7.0 6.5 6.0 5.5 5.0 4.5 4.0 3.5 3.0 2.5 2.0 1.5 1.0 0.5 0
f1 (ppm)

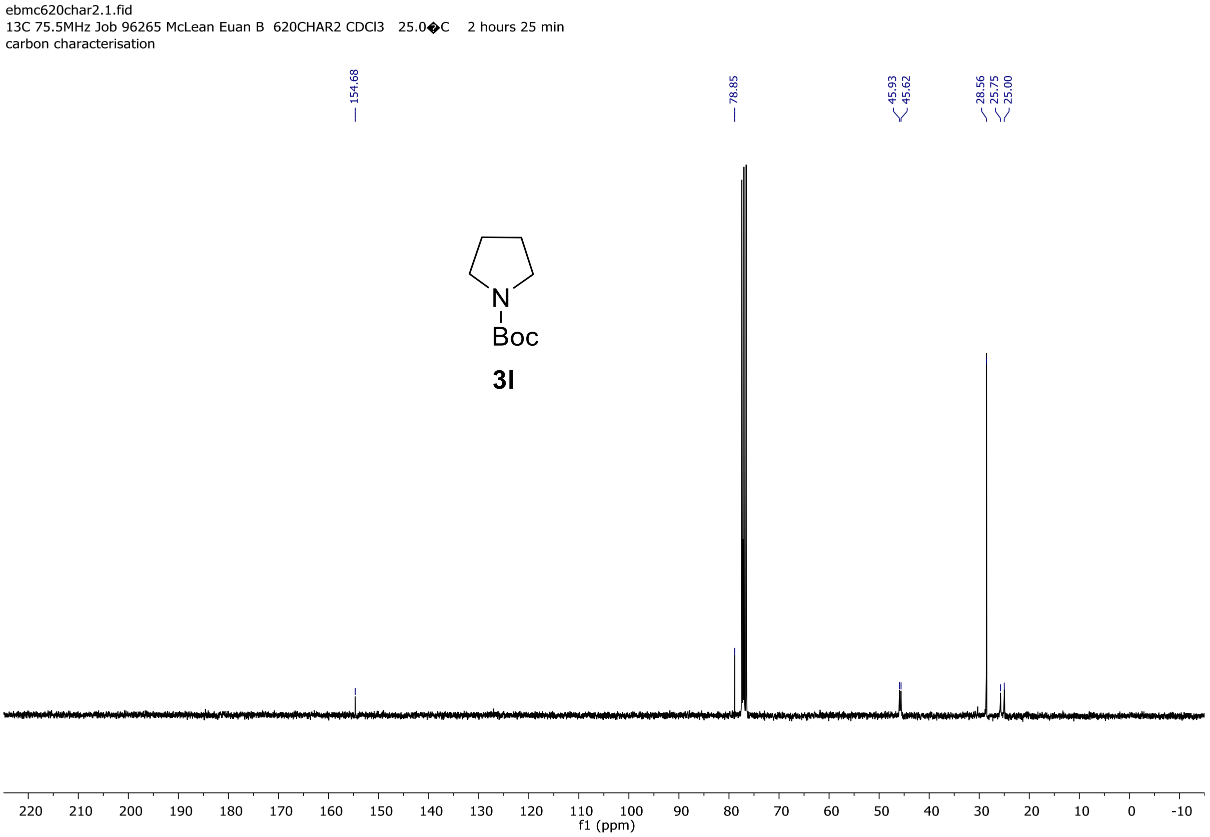
ebmc620char2.1.fid
13C 75.5MHz Job 96265 McLean Euan B 620CHAR2 CDCl3 25.0�C 2 hours 25 min
carbon characterisation
154.68
78.85
45.93
45.62
28.56
25.75
25.00
N
Boc
3l
220 210 200 190 180 170 160 150 140 130 120 110 100 90 80 70 60 50 40 30 20 10 0 -10
f1 (ppm)

ebmh625char.1.fid
1H 300.1MHz Job 96414 McLean Euan B 625CHAR CDCl3 25.4�C
proton characterisation

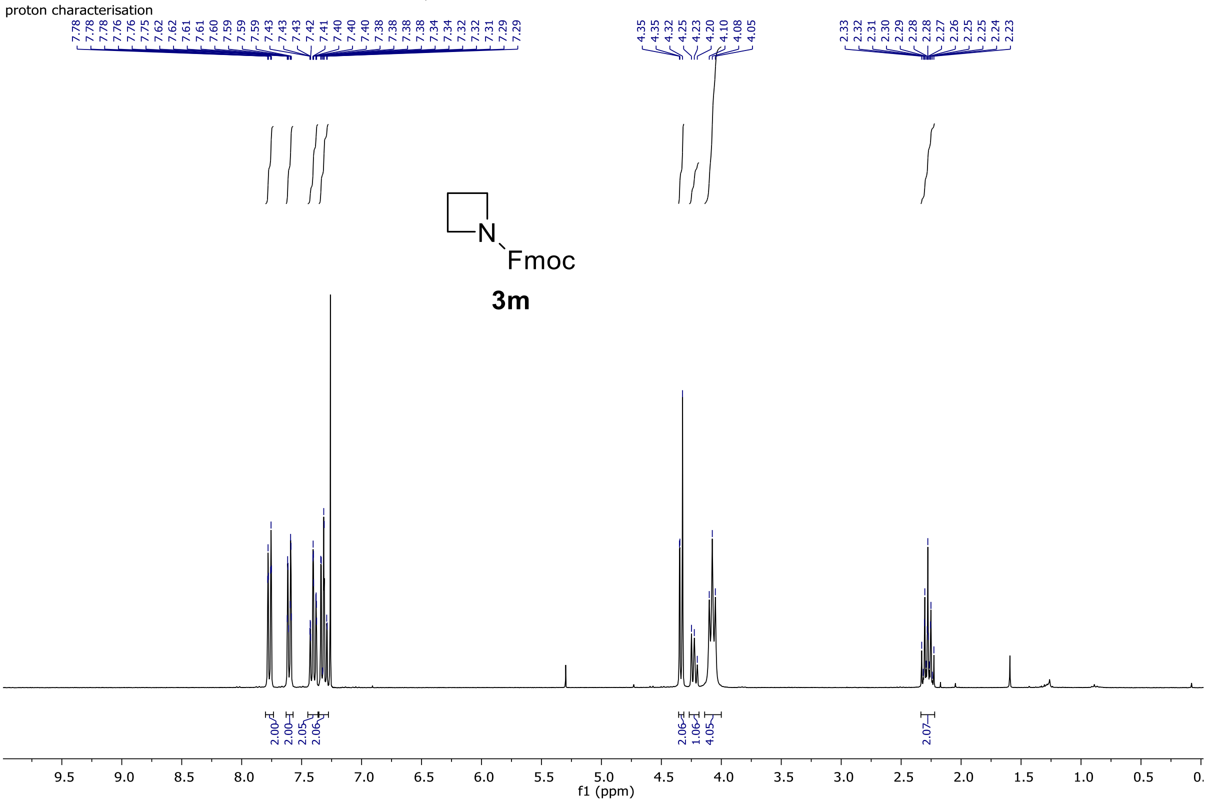

ebmc625char.1.fid
13C 75.5MHz Job 96426 McLean Euan B 625CHAR CDCl3 25.0�C 3 hours 1 min
carbon characterisation

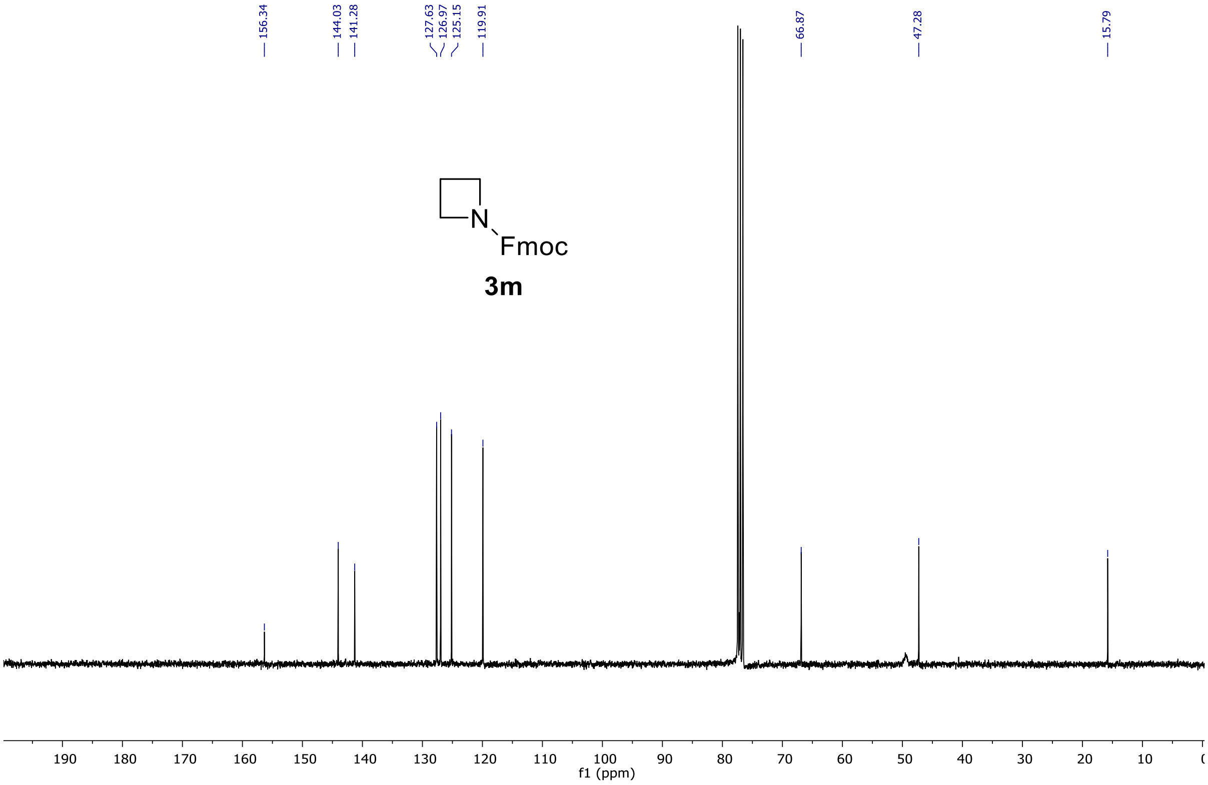

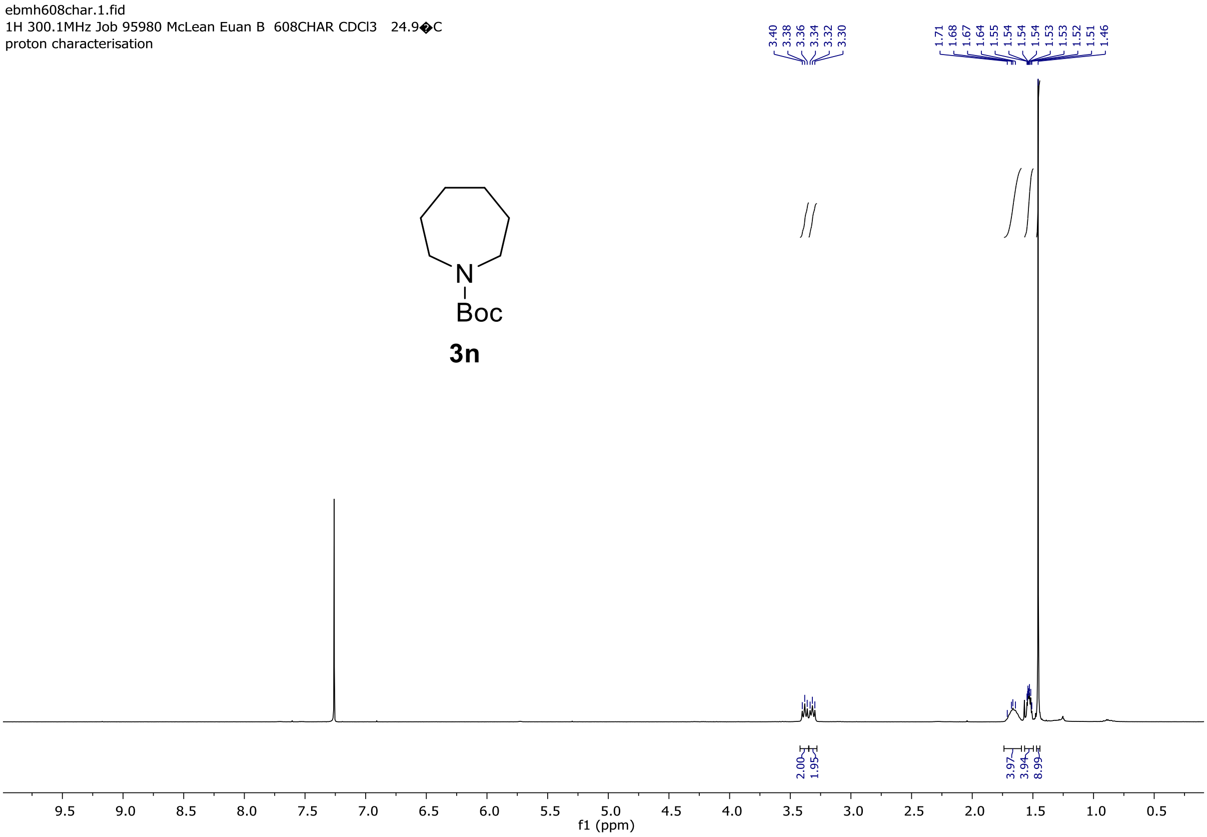
ebmh608char.1.fid
1H 300.1MHz Job 95980 McLean Euan B 608CHAR CDCl3 24.9�C
proton characterisation
3.40
3.38
3.36
3.34
3.32
3.30
1.71
1.68
1.67
1.64
1.55
1.54
1.54
1.54
1.53
1.53
1.52
1.51
1.46
N
Boc
3n
2.00
1.95
3.97
3.94
8.99
9.5
9.0
8.5
8.0
7.5
7.0
6.5
6.0
5.5
5.0
4.5
4.0
3.5
3.0
2.5
2.0
1.5
1.0
0.5
f1 (ppm)

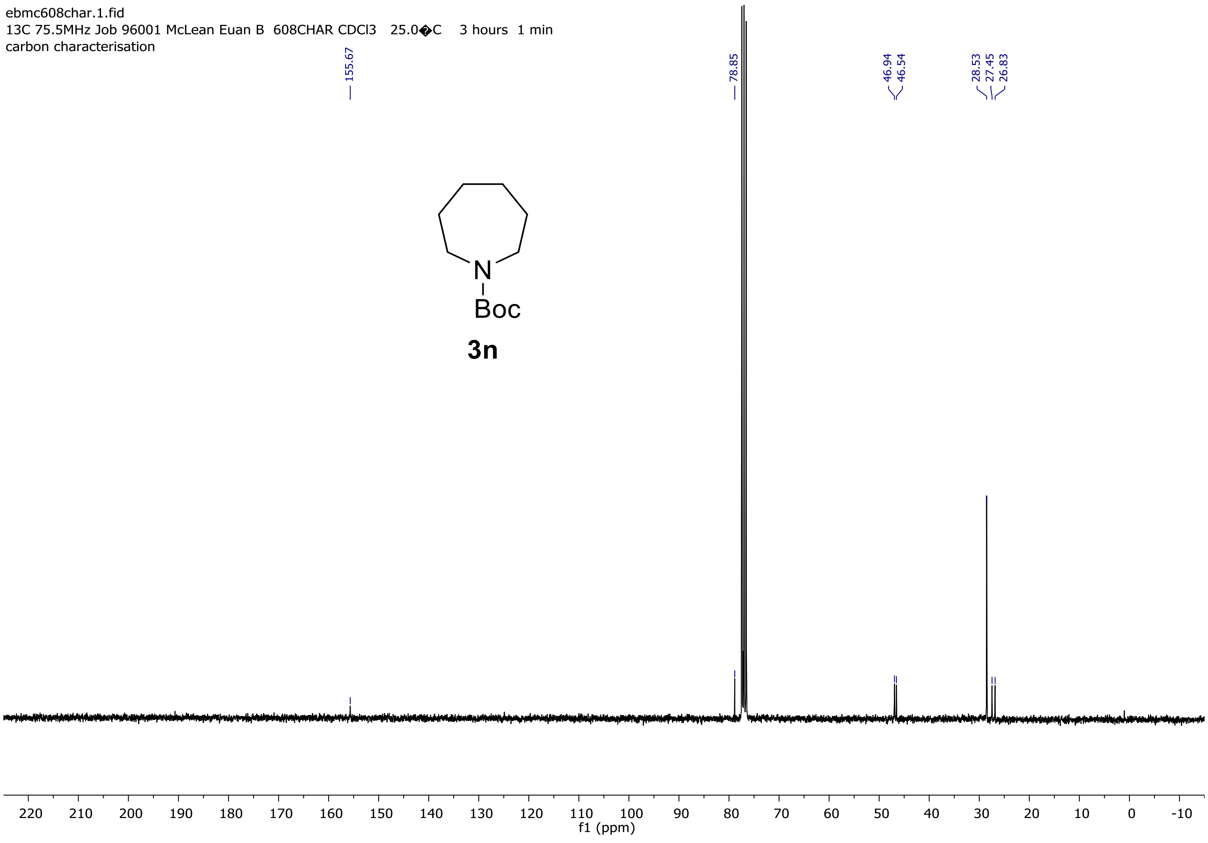
ebmc608char.1.fid
13C 75.5MHz Job 96001 McLean Euan B 608CHAR CDCl3 25.0�C 3 hours 1 min
carbon characterisation
155.67
78.85
46.94
46.54
28.53
27.45
26.83
N
Boc
3n
220
210
200
190
180
170
160
150
140
130
120
110
100
90
80
70
60
50
40
30
20
10
0
-10
f1 (ppm)

ebmh520char.1.fid
1H 300.1MHz Job 94266 McLean Euan B 520CHAR CDCl3   24.7�C
proton characterisation

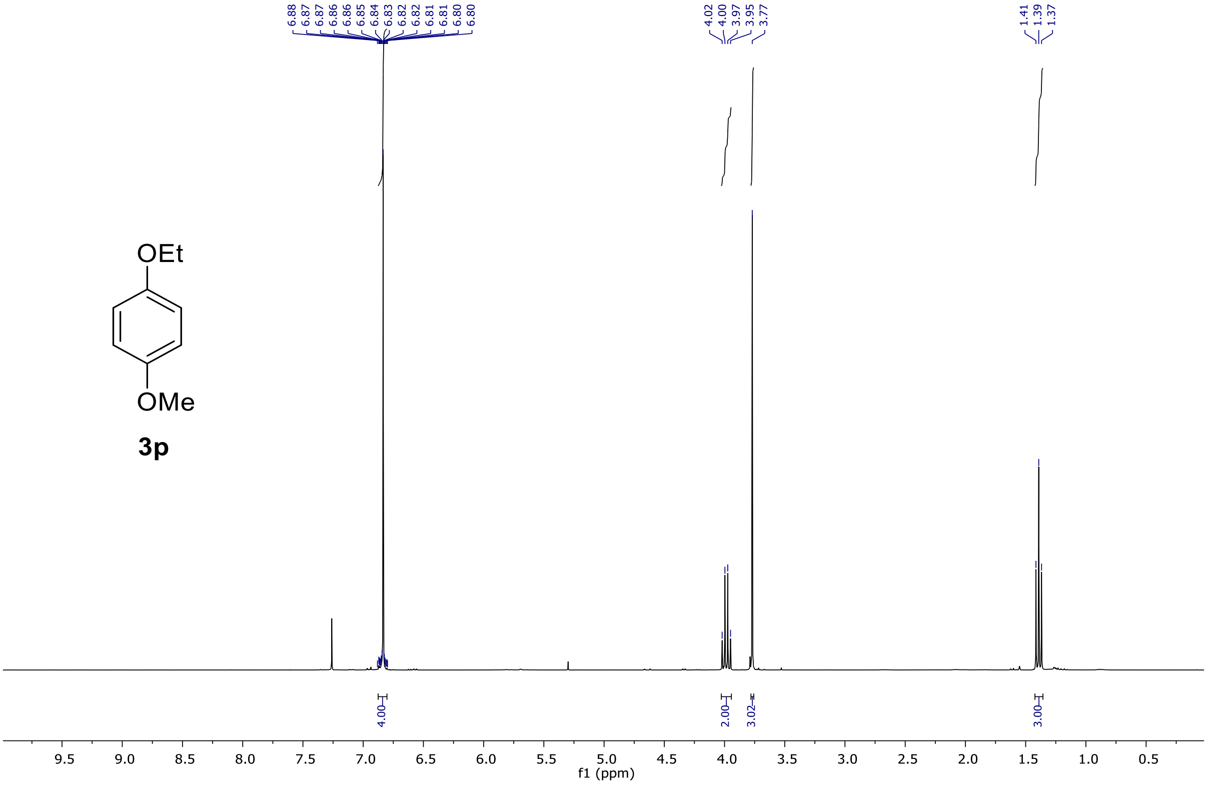

ebmc520char.1.fid
13C 75.5MHz Job 94285 McLean Euan B 520CHAR CDCl3   25.0�C   3 hours 1 min
carbon characterisation

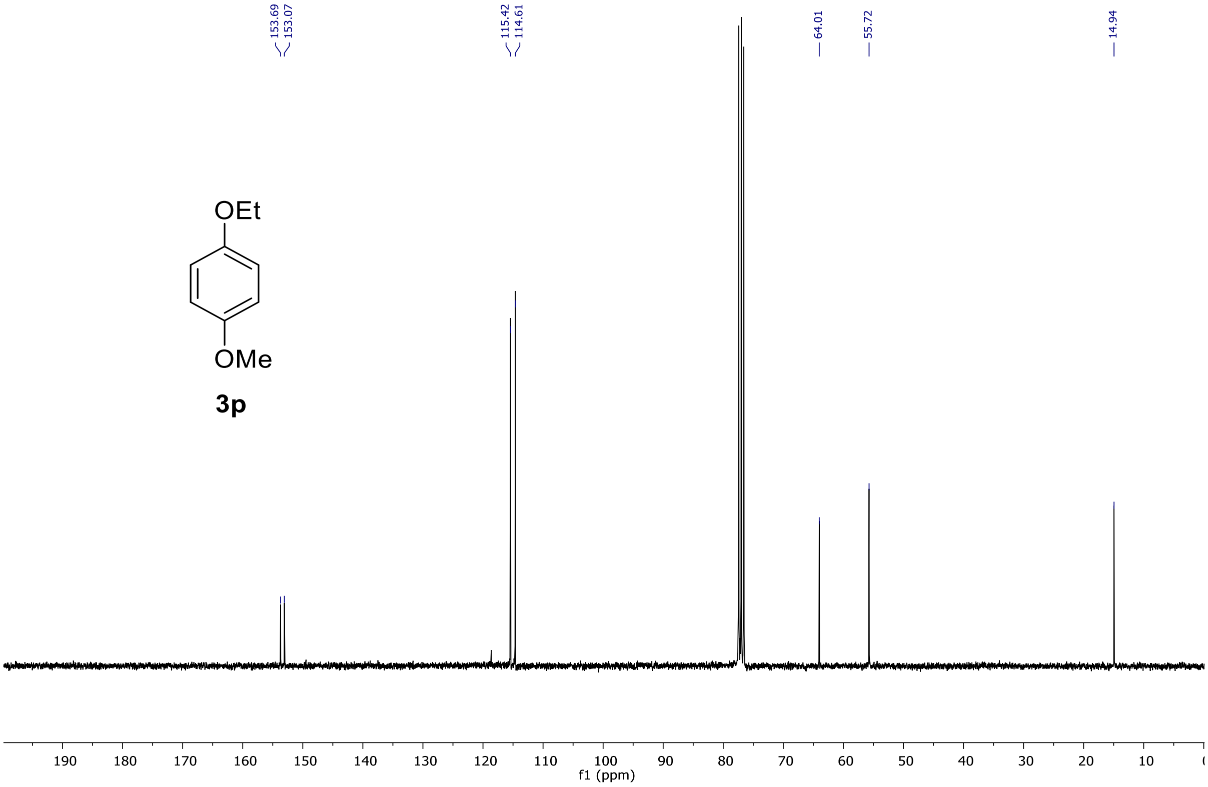

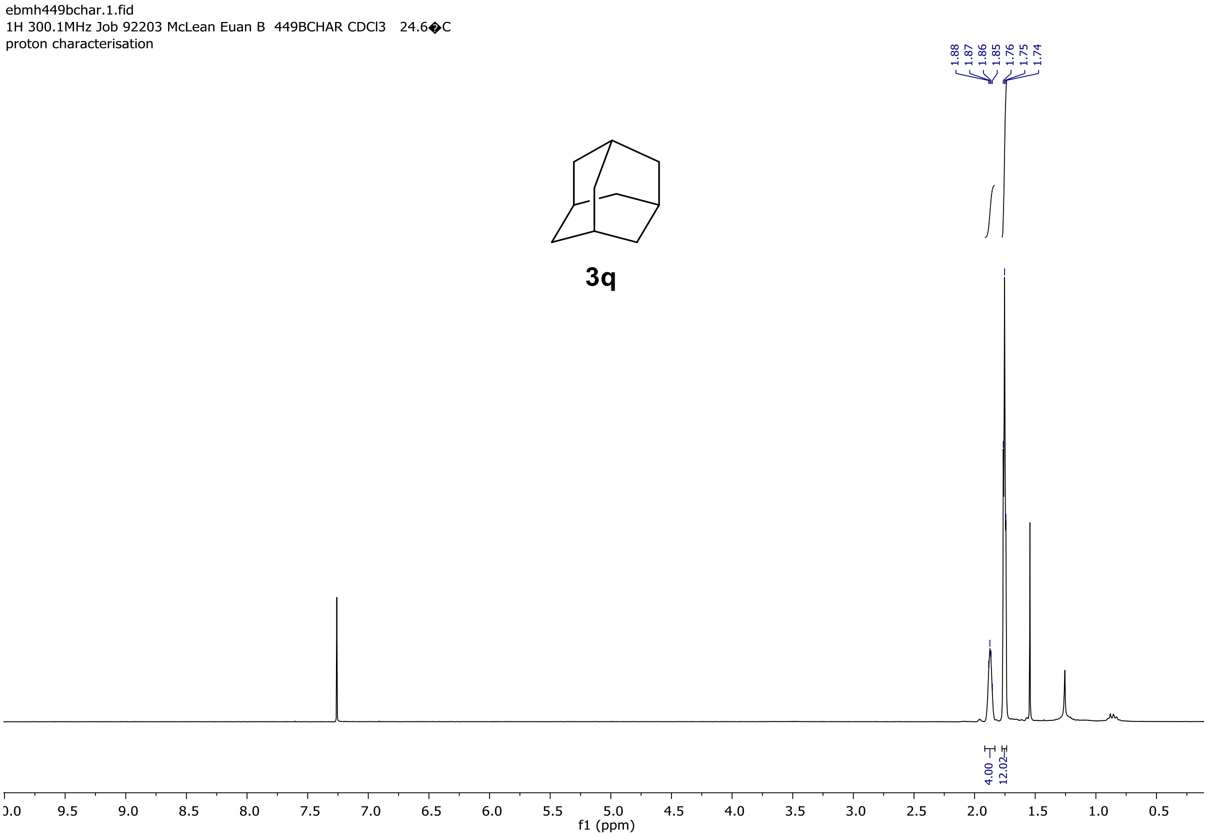
ebmh449bchar.1.fid
1H 300.1MHz Job 92203 McLean Euan B 449BCHAR CDCl3 24.6�C
proton characterisation
1.88
1.87
1.86
1.85
1.76
1.75
1.74
3q
4.00
12.02
f1 (ppm)

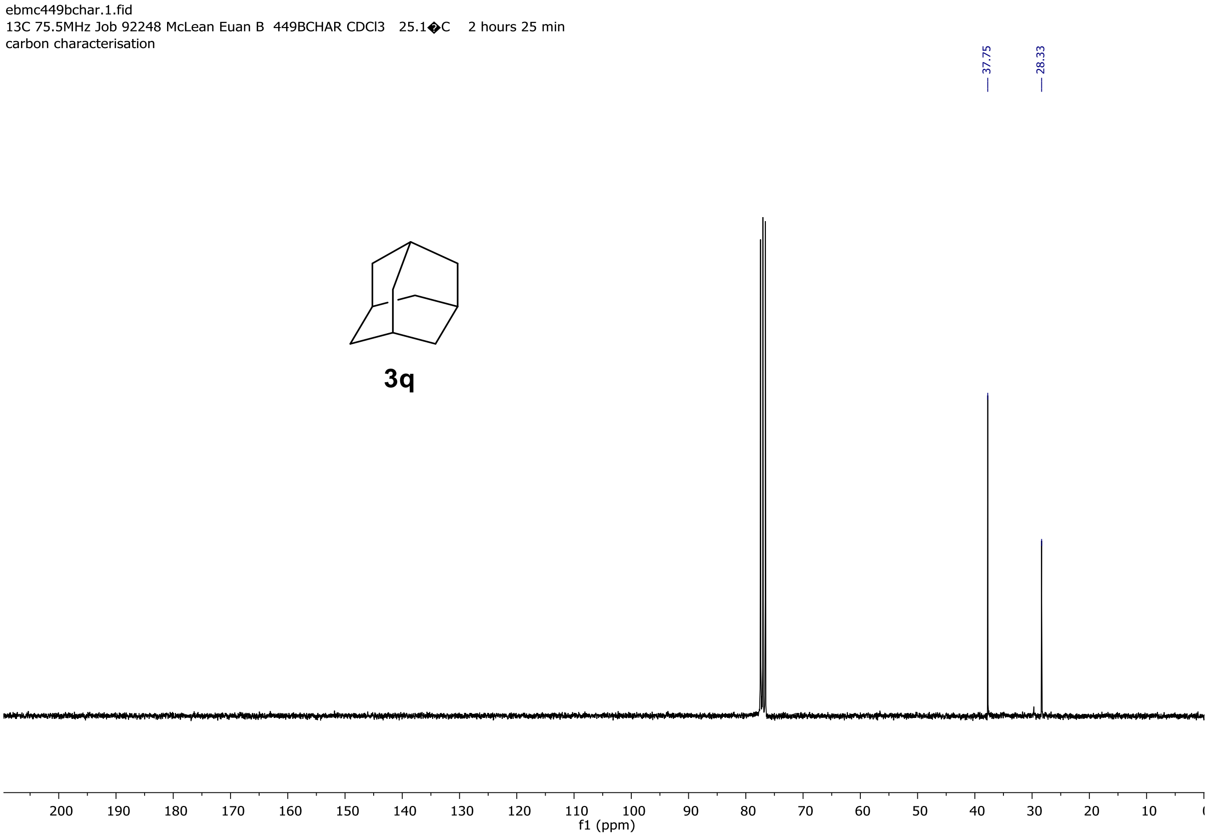
ebmc449bchar.1.fid
13C 75.5MHz Job 92248 McLean Euan B 449BCHAR CDCl3 25.1�C 2 hours 25 min
carbon characterisation
37.75
28.33
3q
f1 (ppm)

ebmh462char.1.fid
1H 300.1MHz Job 93079 McLean Euan B 462CHAR CDCl3 25.4�C
proton characterisation

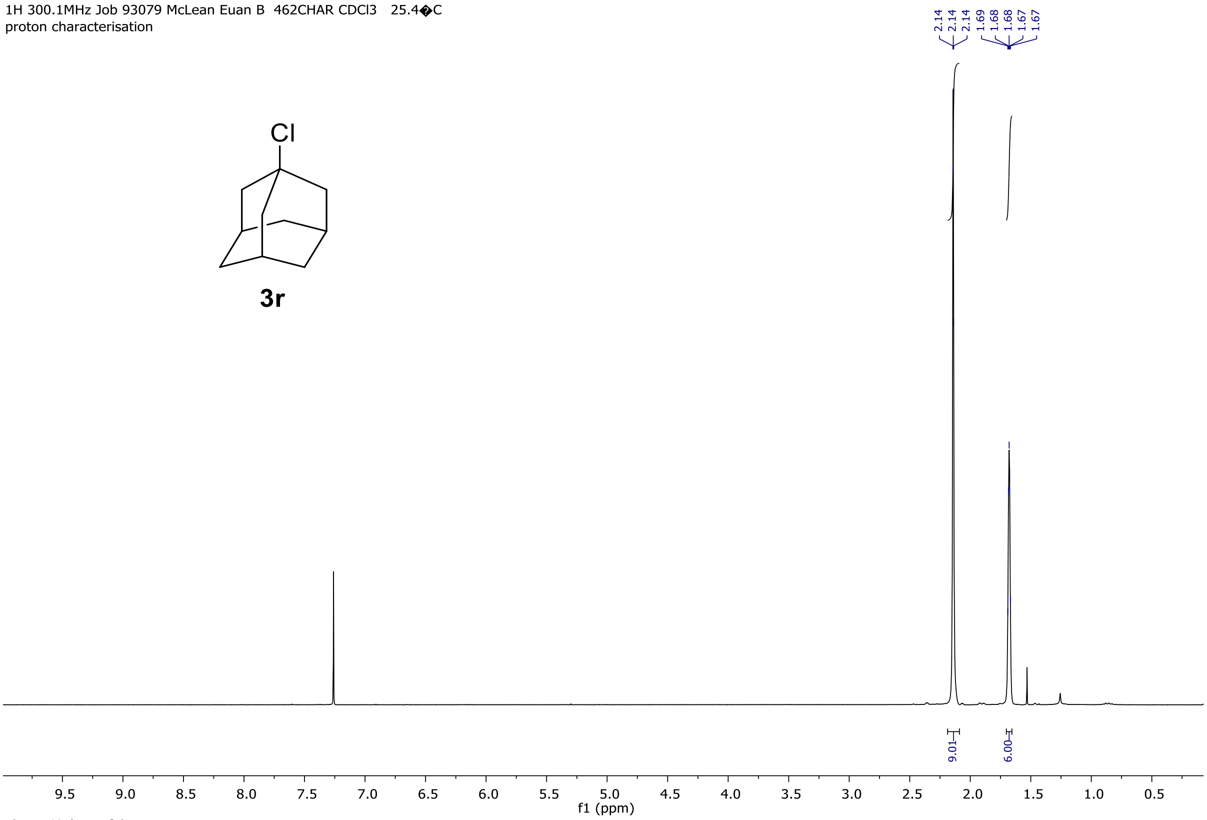

ebmc462char.1.fid
13C 75.5MHz Job 93124 McLean Euan B 462CHAR CDCl3 25.0�C 3 hours 1 min
carbon characterisation

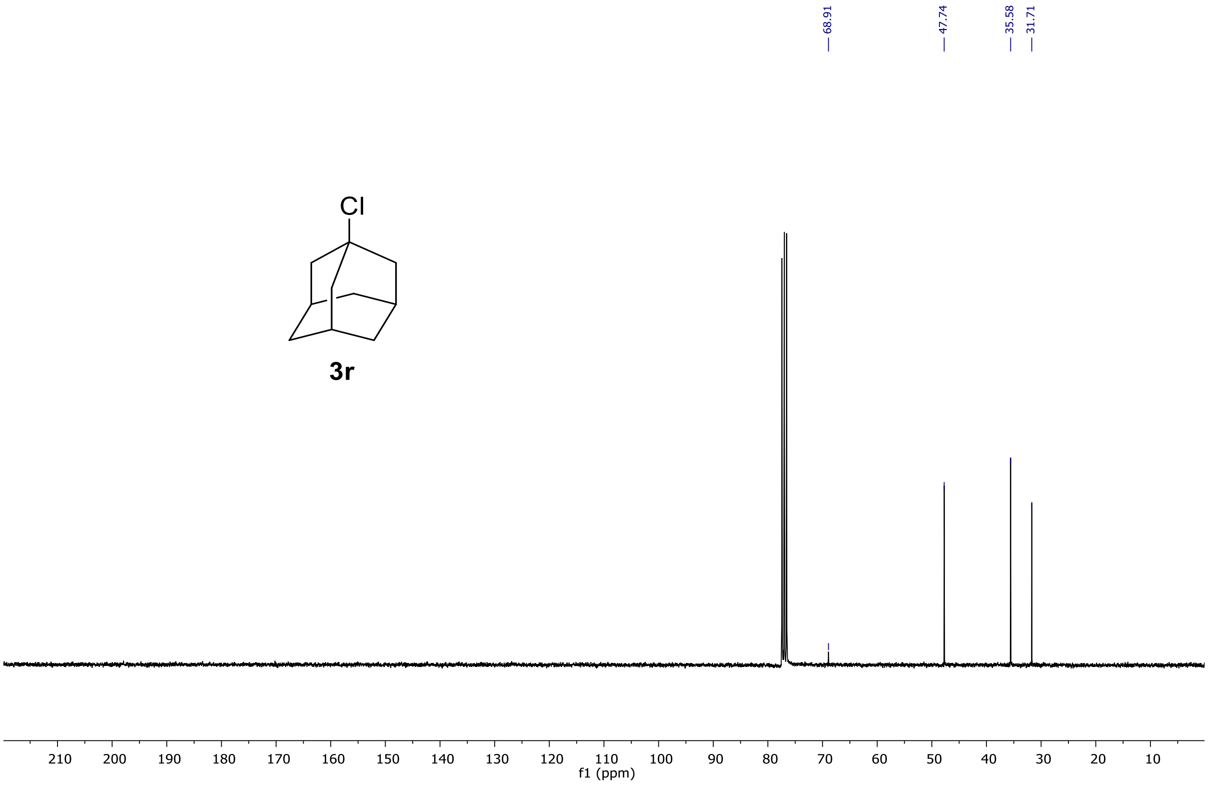

ebmh474bchar.1.fid
1H 400.1MHz Job 41562 McLean Euan B 474BCHAR CDCl3 24.8�C
proton characterisation

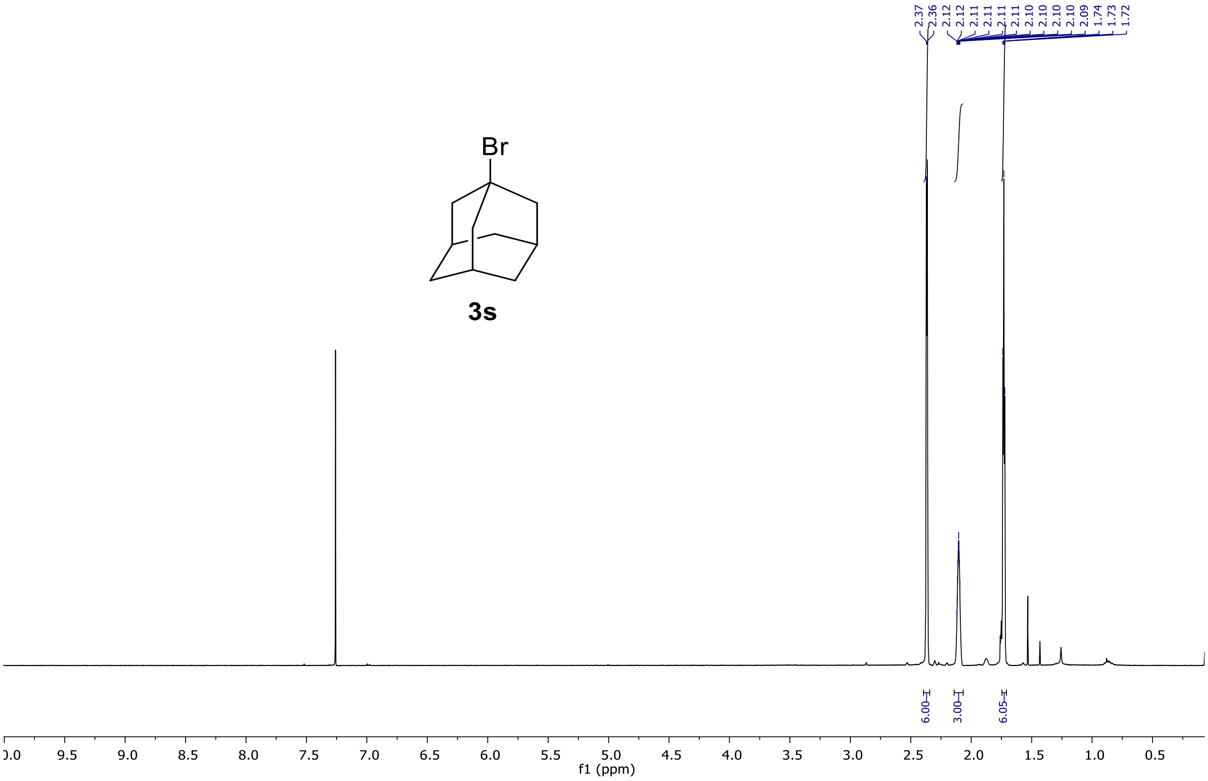

ebmc474bchar.1.fid
13C 100.6MHz Job 41575 McLean Euan B 474BCHAR CDCl3 26.3�C 0 hour 58 min
carbon characterisation

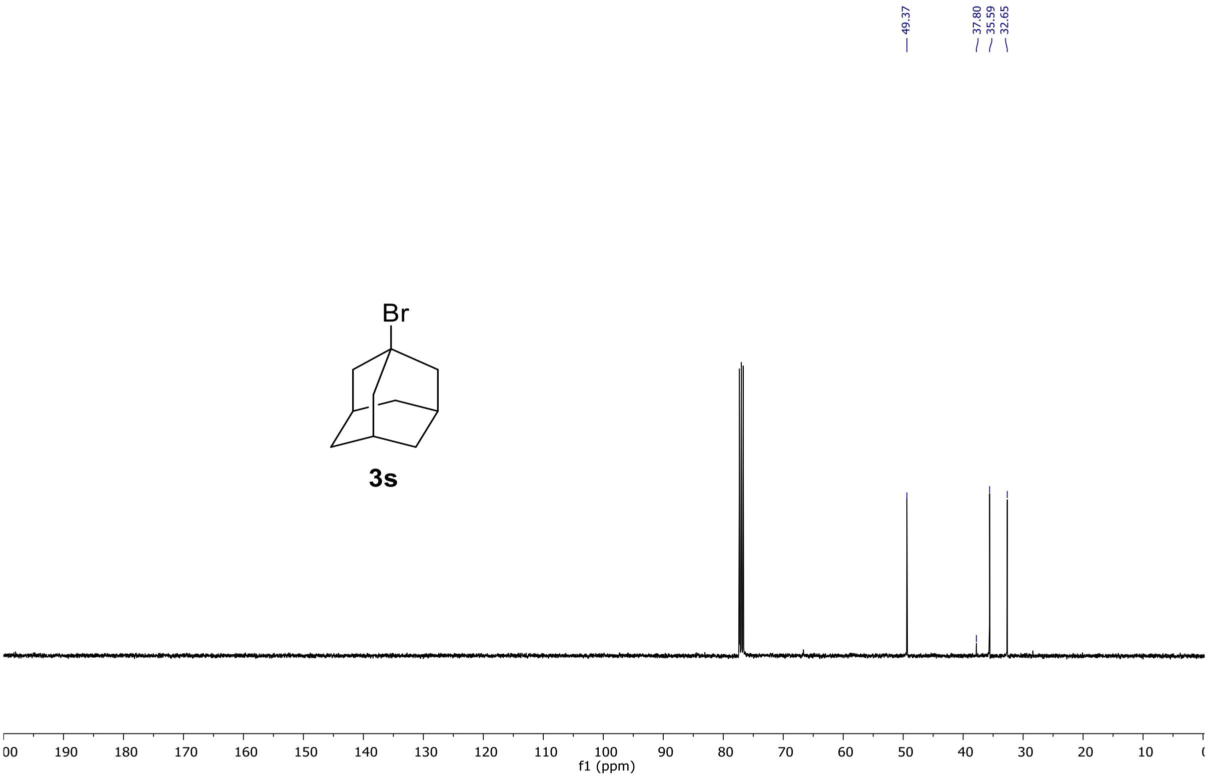

ebmh467char.1.fid
1H 300.1MHz Job 93081 McLean Euan B 467CHAR CDCl3 25.4�C
proton characterisation

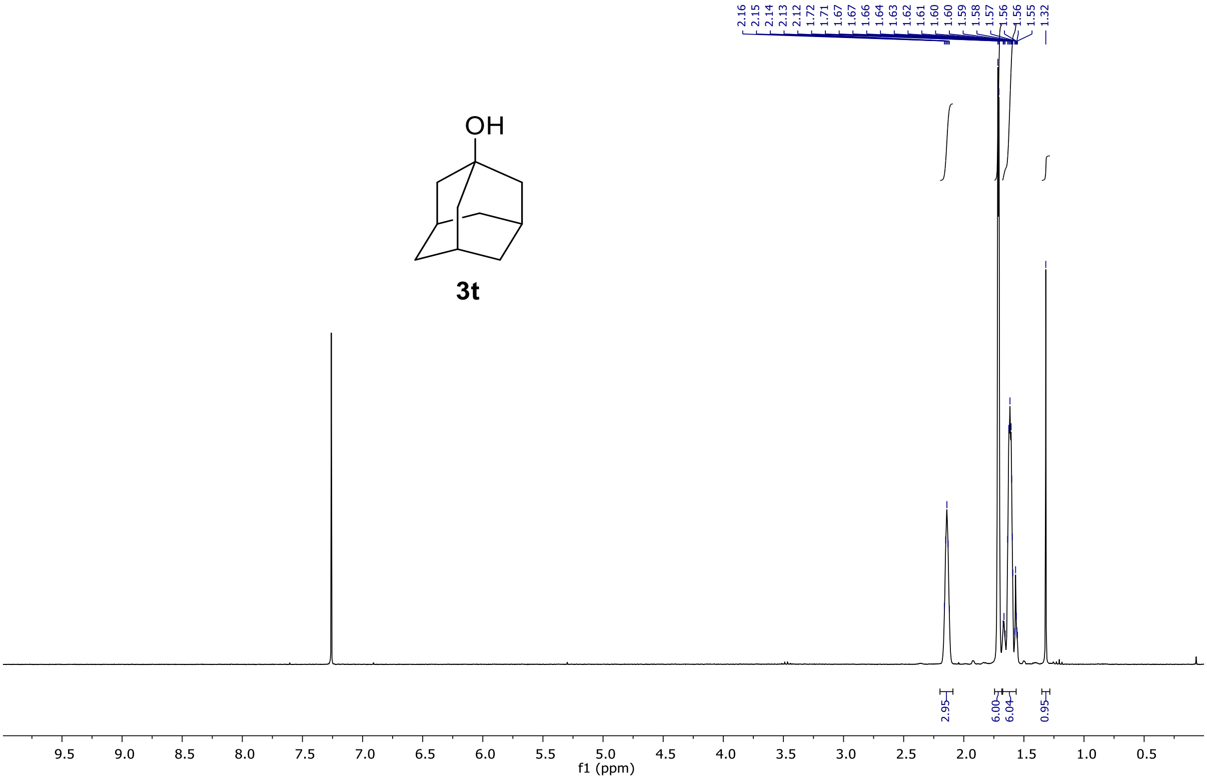

ebmc467char.1.fid
13C 75.5MHz Job 93125 McLean Euan B 467CHAR CDCl3 25.0�C 3 hours 1 min
carbon chracterisation

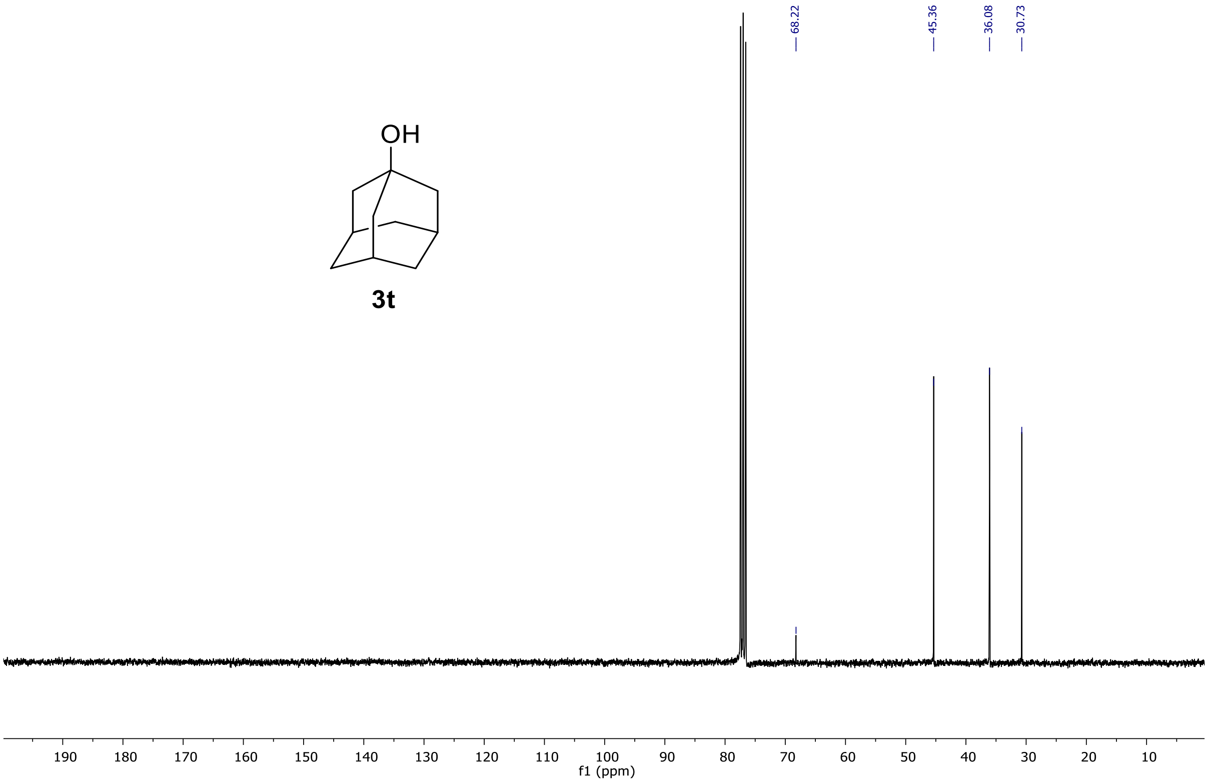

ebmh423char2.1.fid
1H 300.1MHz Job 92213 McLean Euan B 423CHAR2 CDCl3 24.6�C
proton characterisation

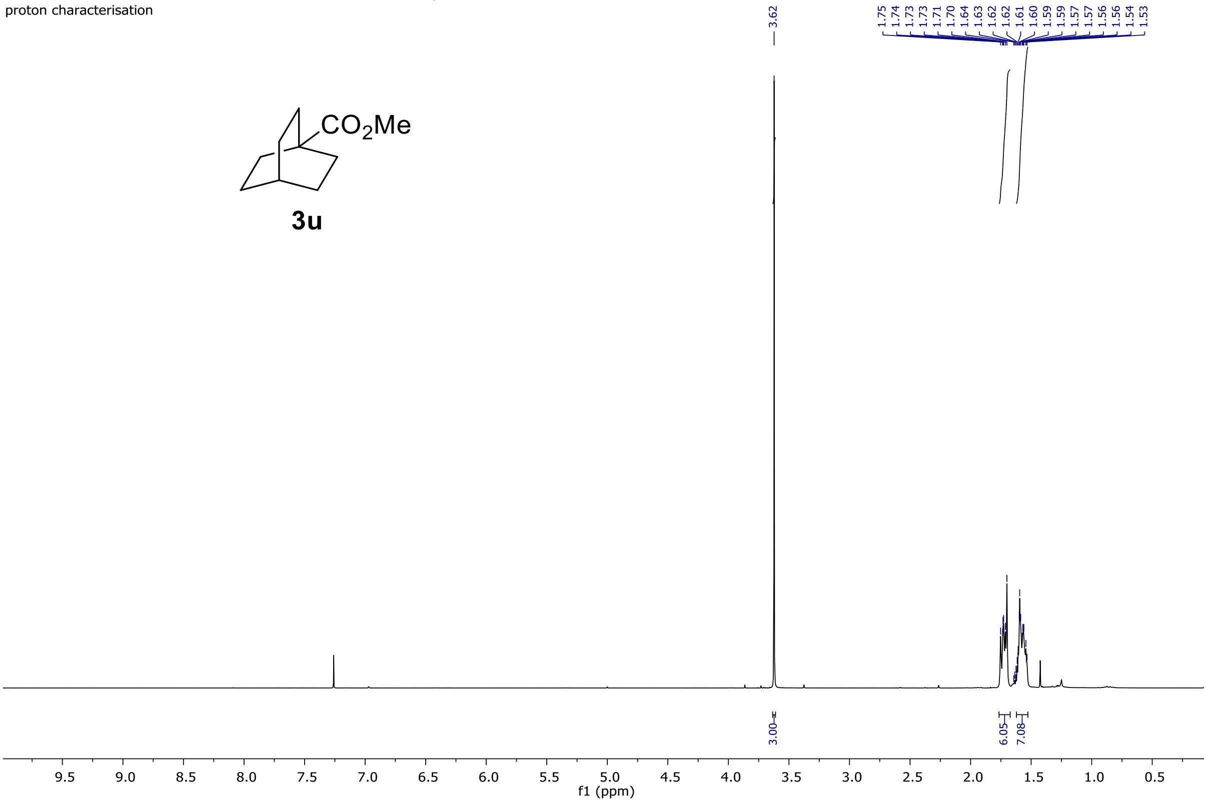

ebmc423char2.1.fid
13C 75.5MHz Job 92249 McLean Euan B 423CHAR2 CDCl3 25.0�C 0 hour 36 min
carbon characterisation

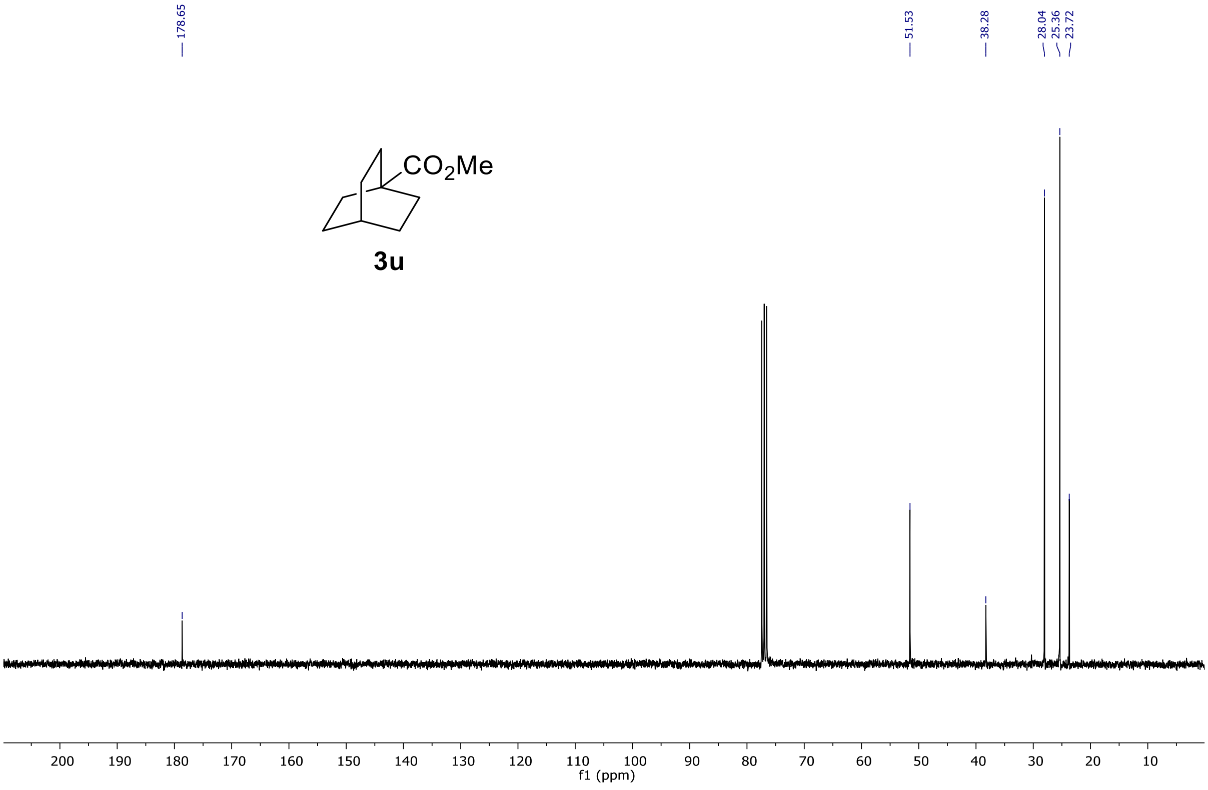

ebmh452char.1.fid
1H 300.1MHz Job 92790 McLean Euan B 452CHAR CDCl3 25.4�C
proton characterisation

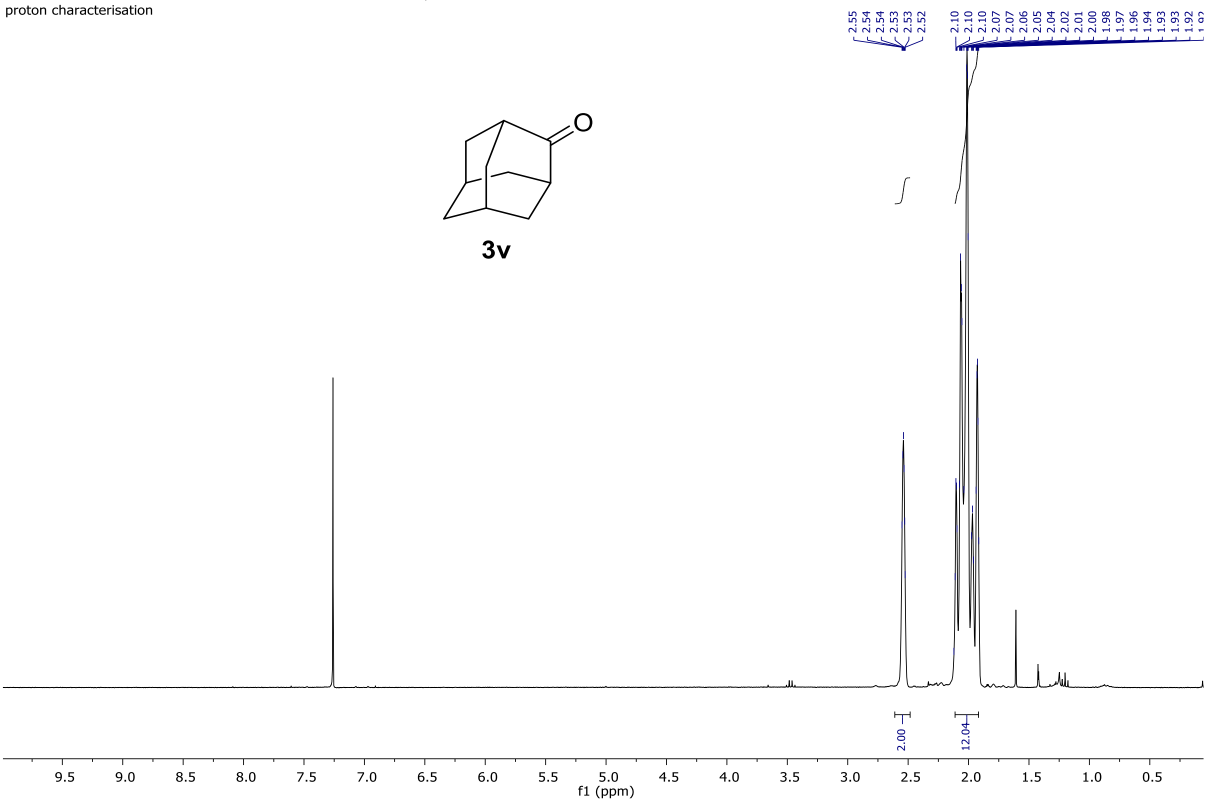

ebmc452char.1.fid
13C 75.5MHz Job 92852 McLean Euan B 452CHAR CDCl3 25.0�C 0 hour 54 min
carbon characterisation

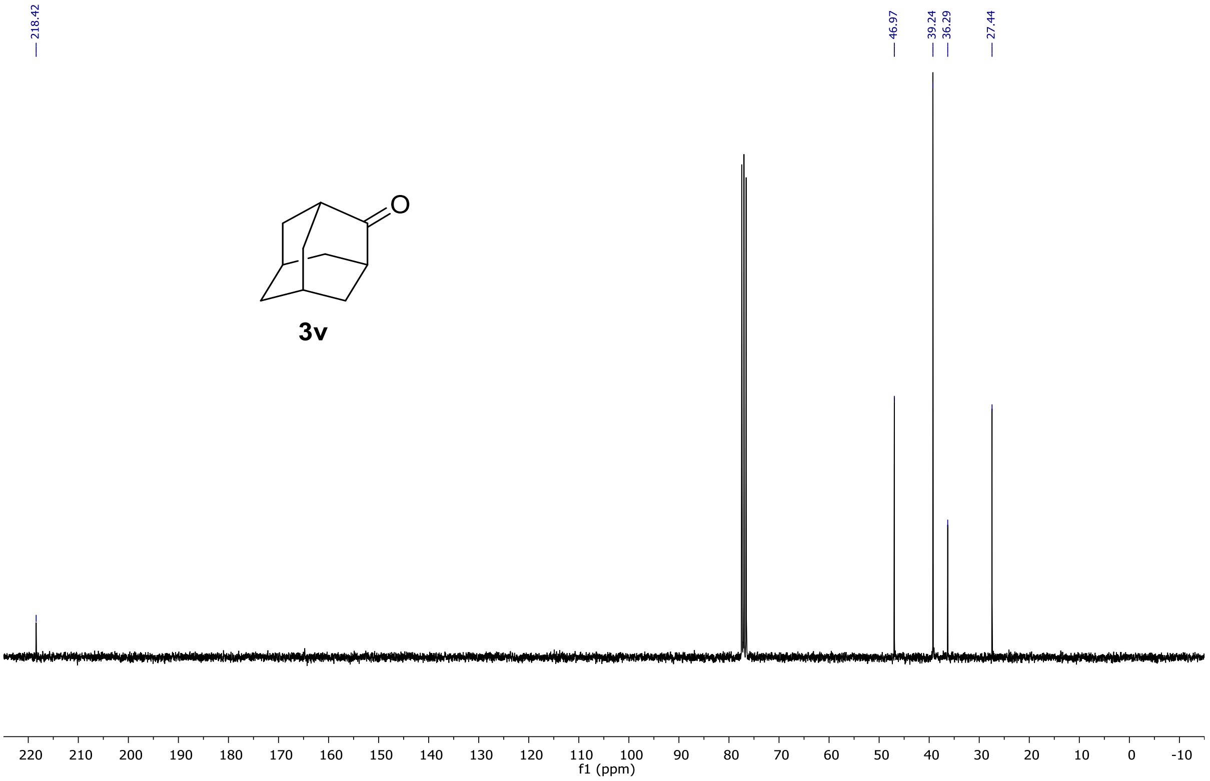

ebmh539char.1.fid
1H 300.1MHz Job 94606 McLean Euan B 539CHAR CDCl3 25.1�C
proton characterisation

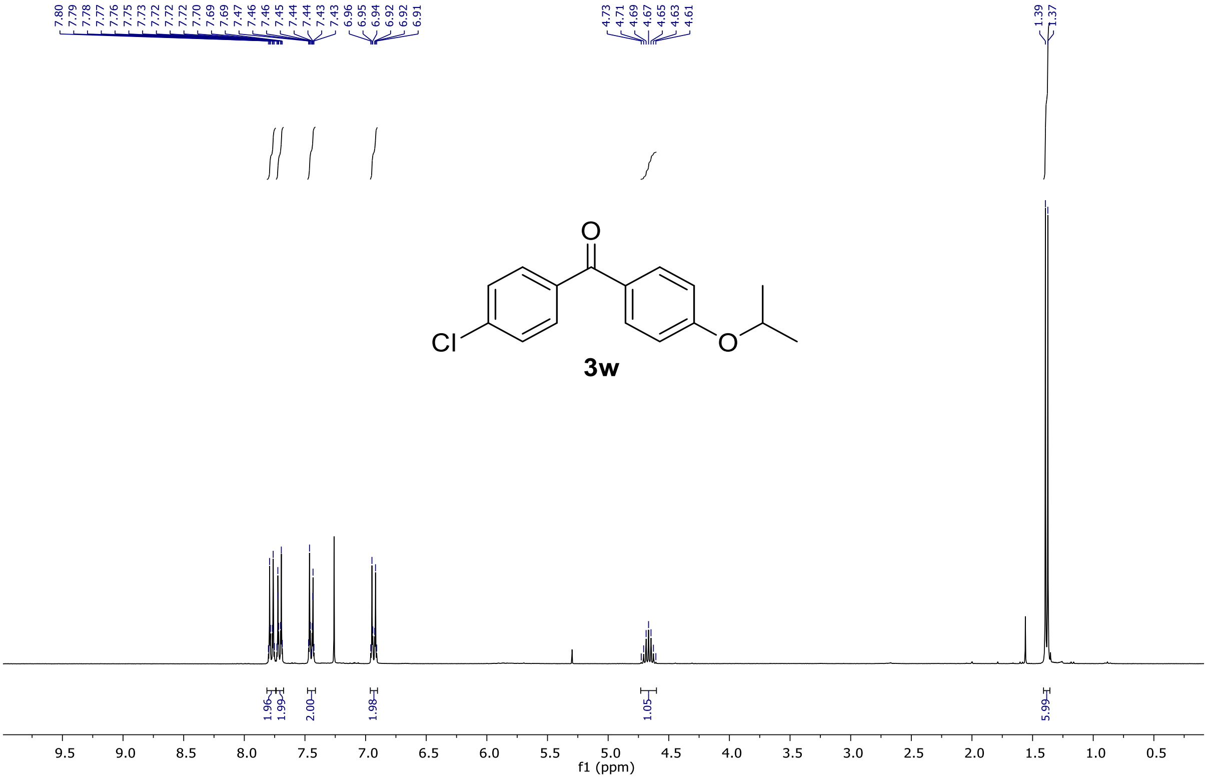

ebmc539char.1.fid
13C 75.5MHz Job 94617 McLean Euan B 539CHAR CDCl3 25.0�C 3 hours 1 min
carbon characterisation

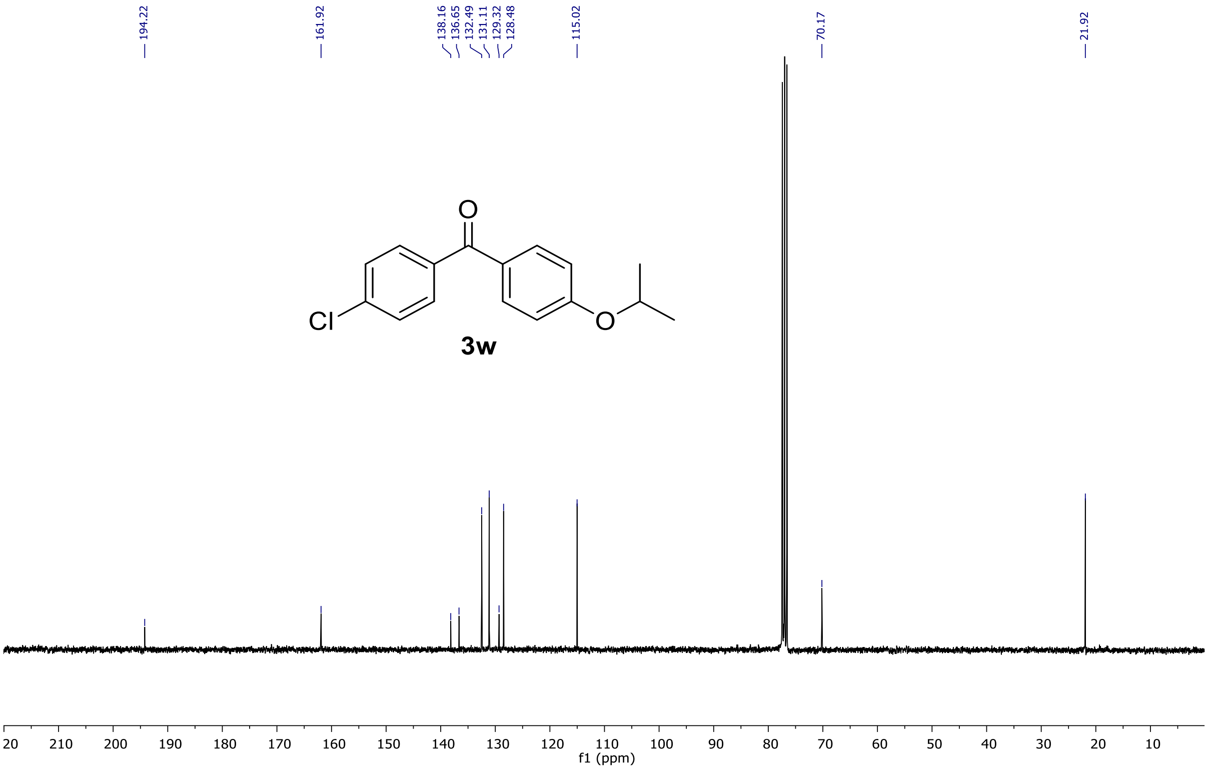

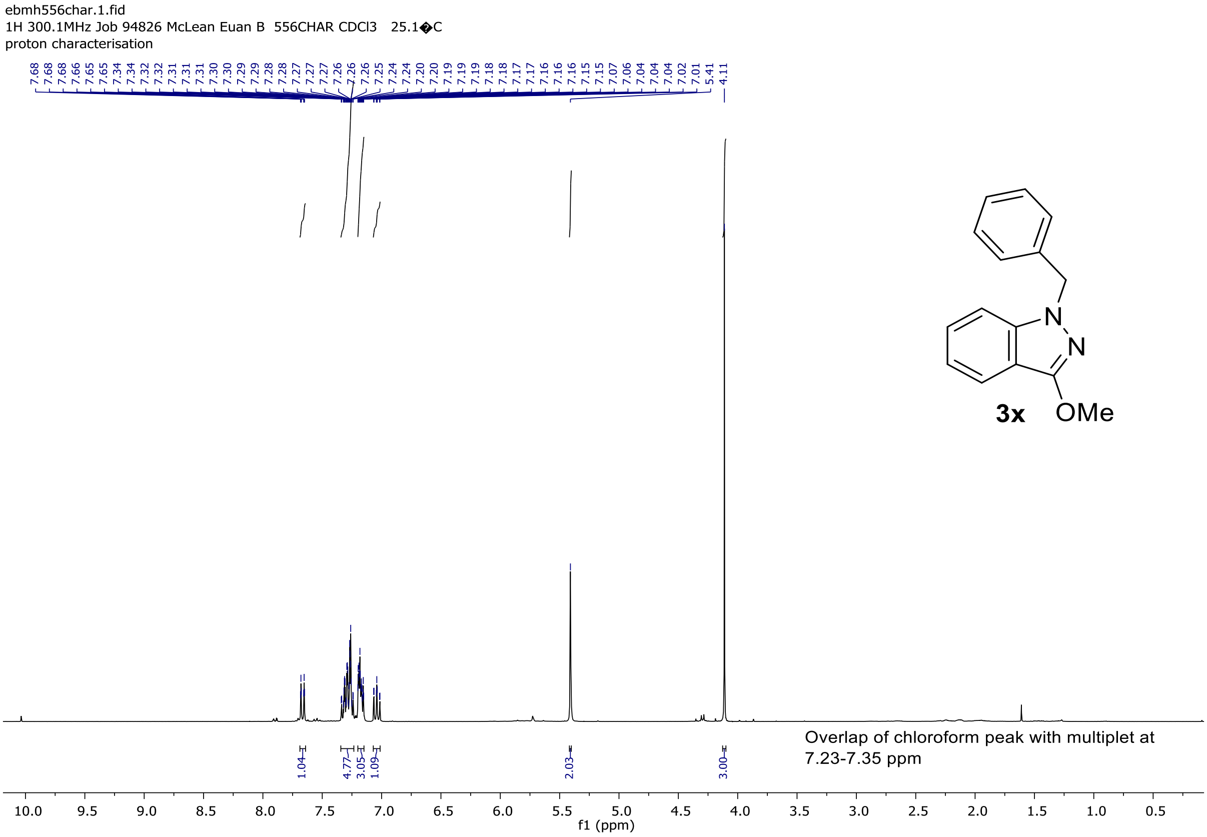
ebmh556char.1.fid
1H 300.1MHz Job 94826 McLean Euan B 556CHAR CDCl3 25.1�C
proton characterisation
5.41
4.11
1.04
4.77
3.05
1.09
2.03
3.00
3x
OMe
N
N
Overlap of chloroform peak with multiplet at 7.23-7.35 ppm
f1 (ppm)

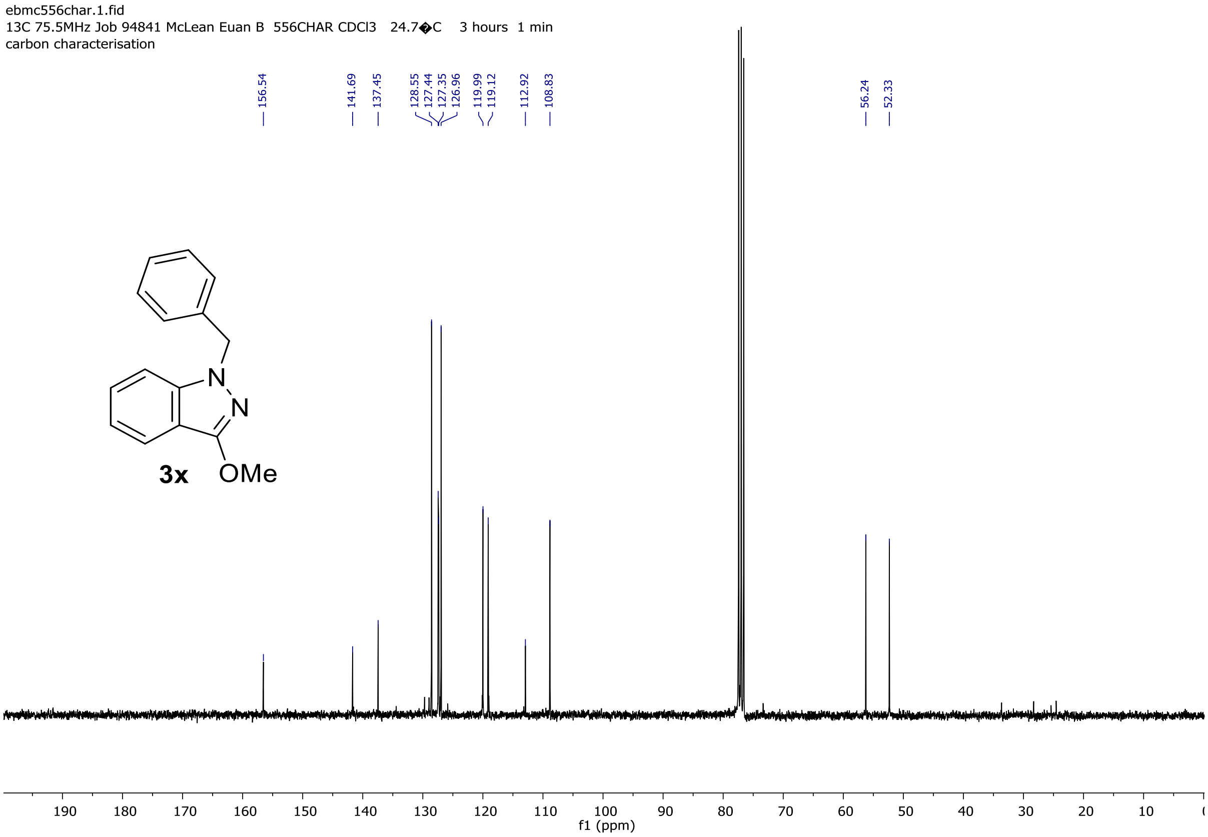
ebmc556char.1.fid
13C 75.5MHz Job 94841 McLean Euan B 556CHAR CDCl3 24.7�C 3 hours 1 min
carbon characterisation
156.54
141.69
137.45
128.55
127.44
127.35
126.96
119.99
119.12
112.92
108.83
56.24
52.33
3x
OMe
N
N
f1 (ppm)

ebmh604char2.1.fid
1H 300.1MHz Job 95820 McLean Euan B 604CHAR2 CDCl3 25.1�C
proton charcaterisation

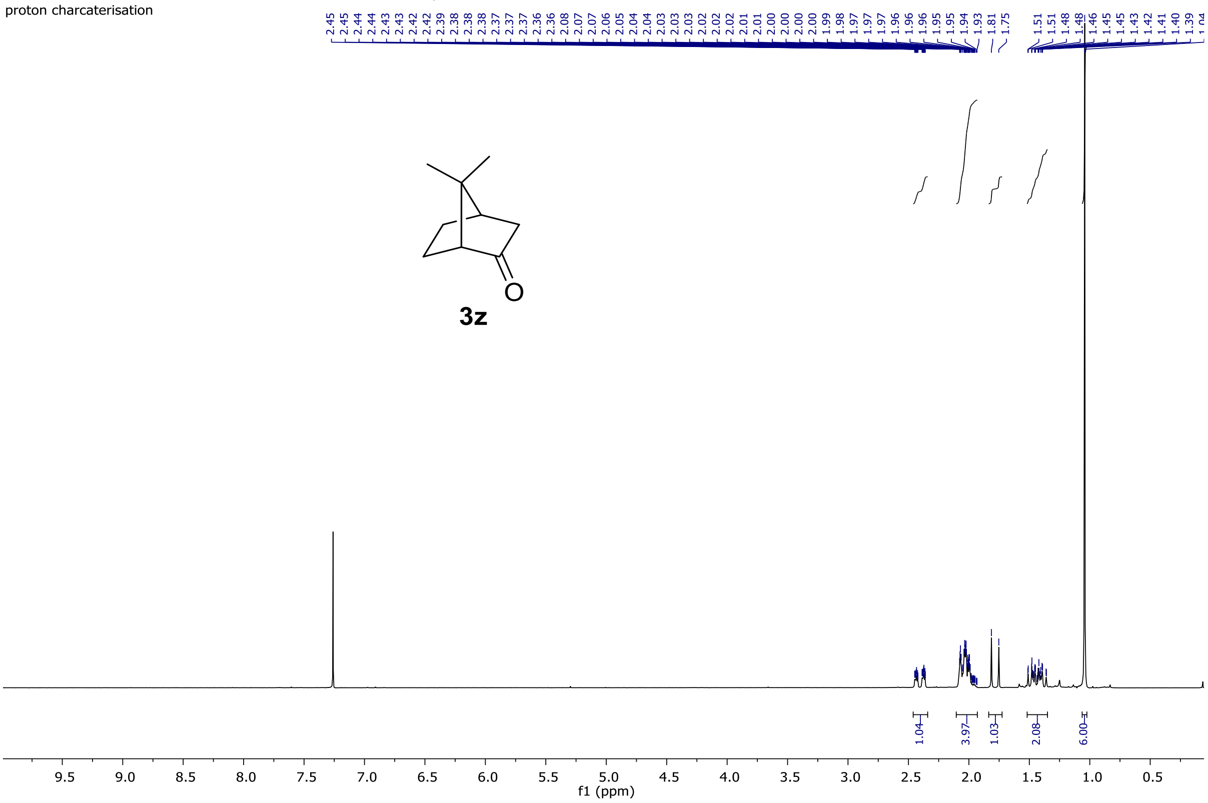

ebmc604char2.1.fid
13C 75.5MHz Job 95833 McLean Euan B 604CHAR2 CDCl3 25.0�C 3 hours 1 min
carbon characterisation

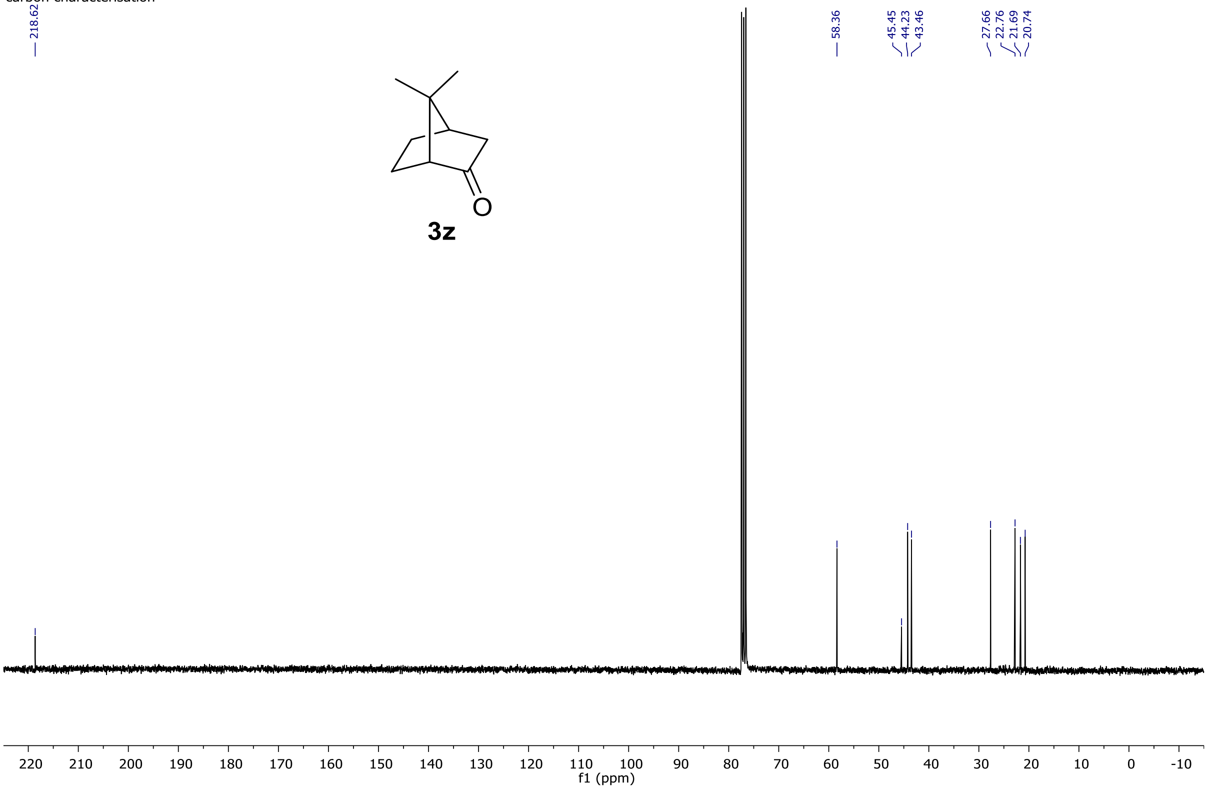

ebmh612char.1.fid
1H 300.1MHz Job 96165 McLean Euan B 612CHAR CDCl3 25.1�C
proton characterisation

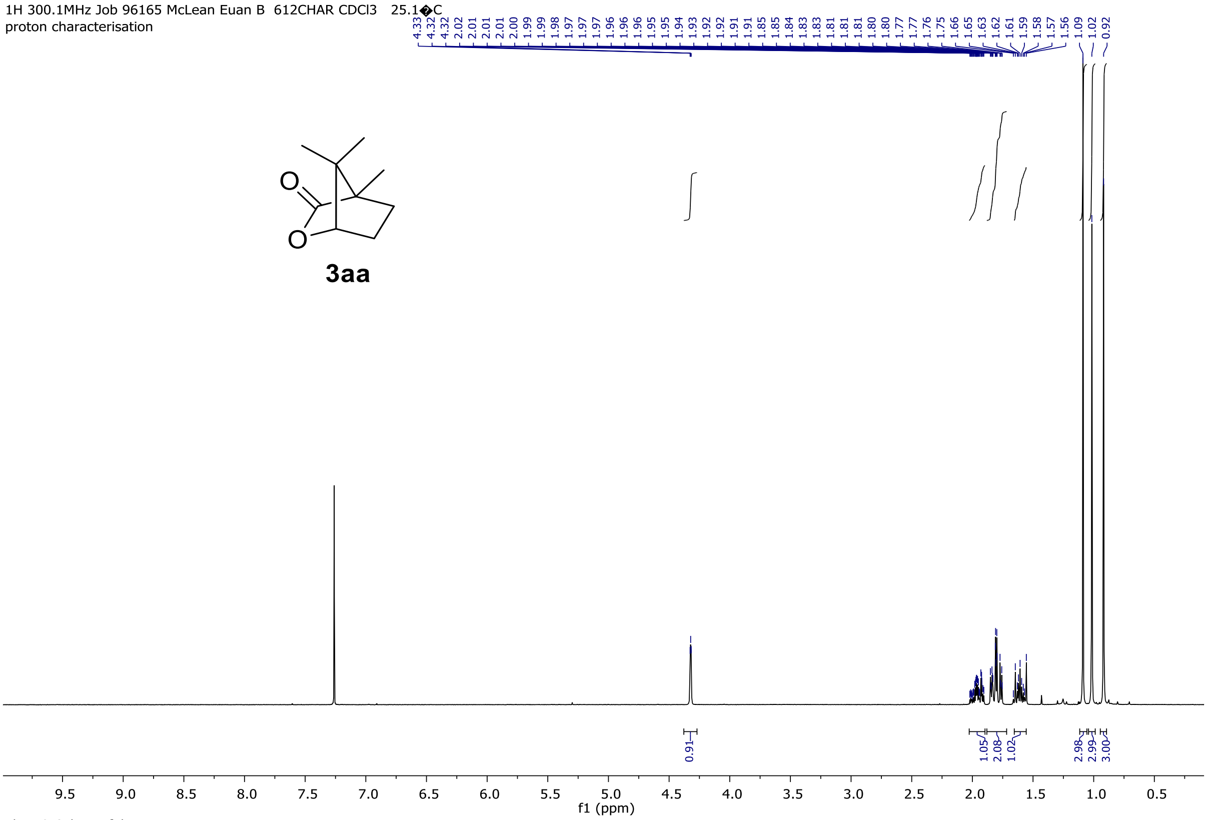

ebmc612char.1.fid
13C 75.5MHz Job 96186 McLean Euan B 612CHAR CDCl3 25.0�C 3 hours 1 min
carbon characterisation

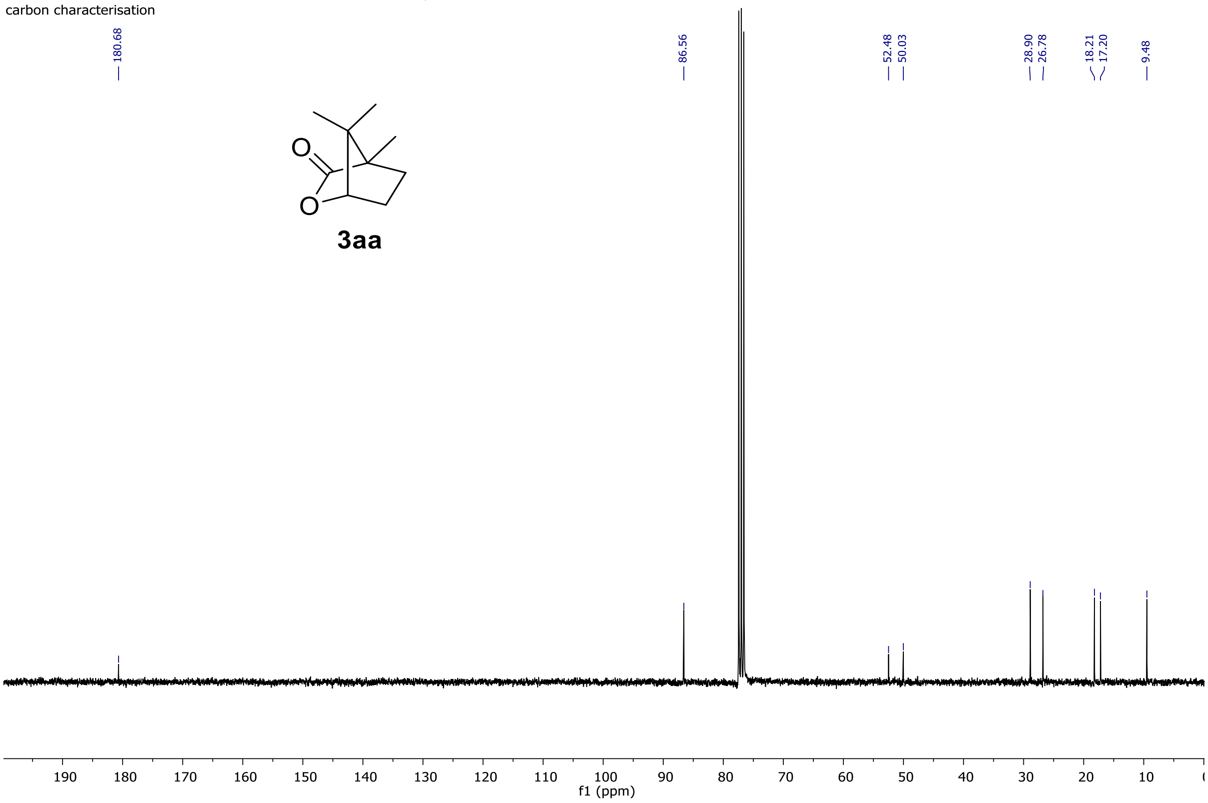

ebmh628bcha2.1.fid
1H 300.1MHz Job 96474 McLean Euan B 628BCHA2 CDCl3 25.4�C
proton characterisation

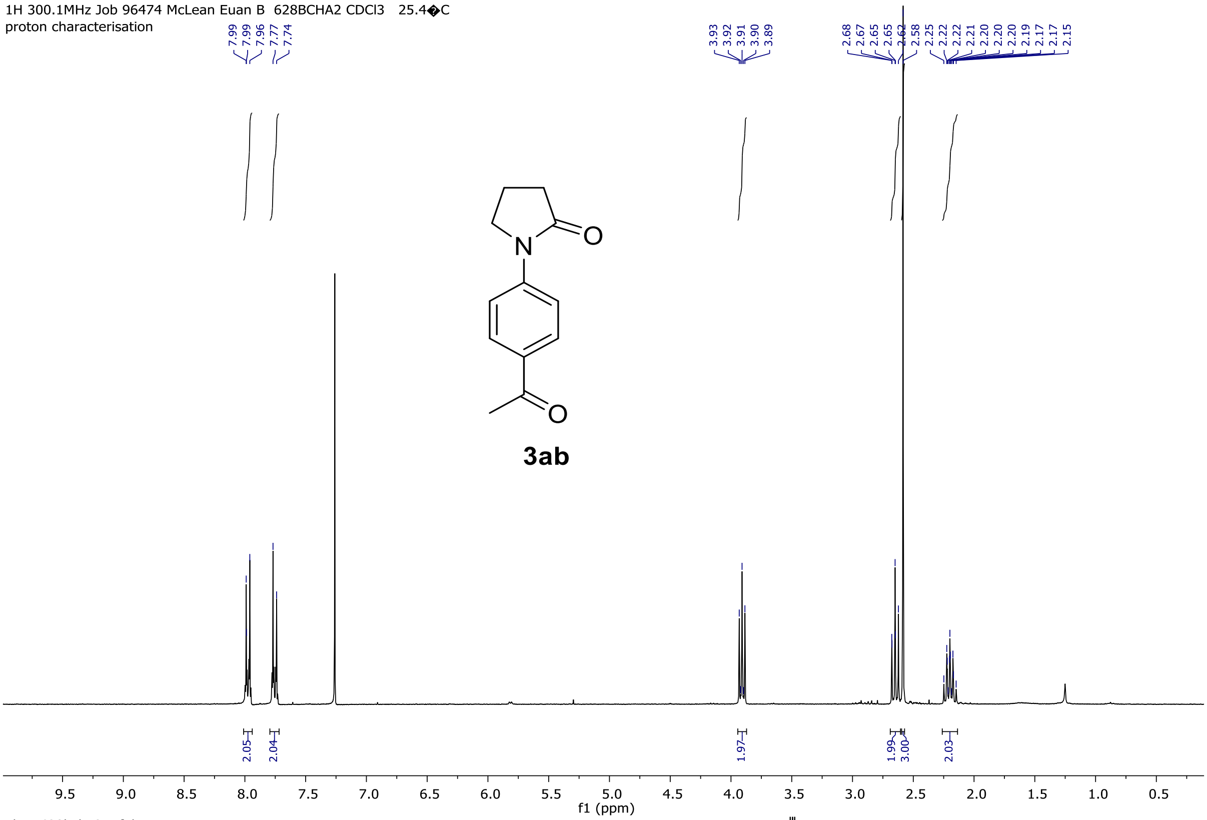

ebmc628bcha2.1.fid
13C 75.5MHz Job 96483 McLean Euan B 628BCHA2 CDCl3 25.1�C 3 hours 1 min
carbon characterisation

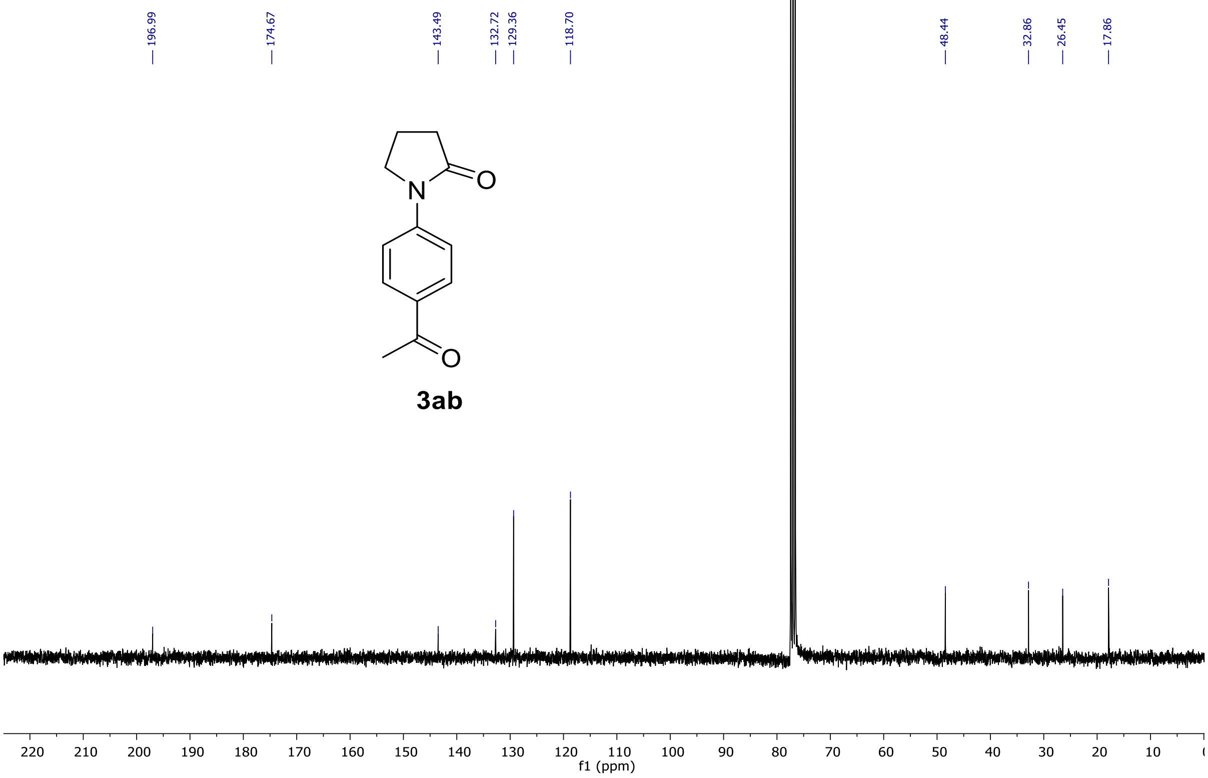

ebmh609char.1.fid
1H 300.1MHz Job 95948 McLean Euan B 609CHAR CDCl3 25.2�C
proton characterisation

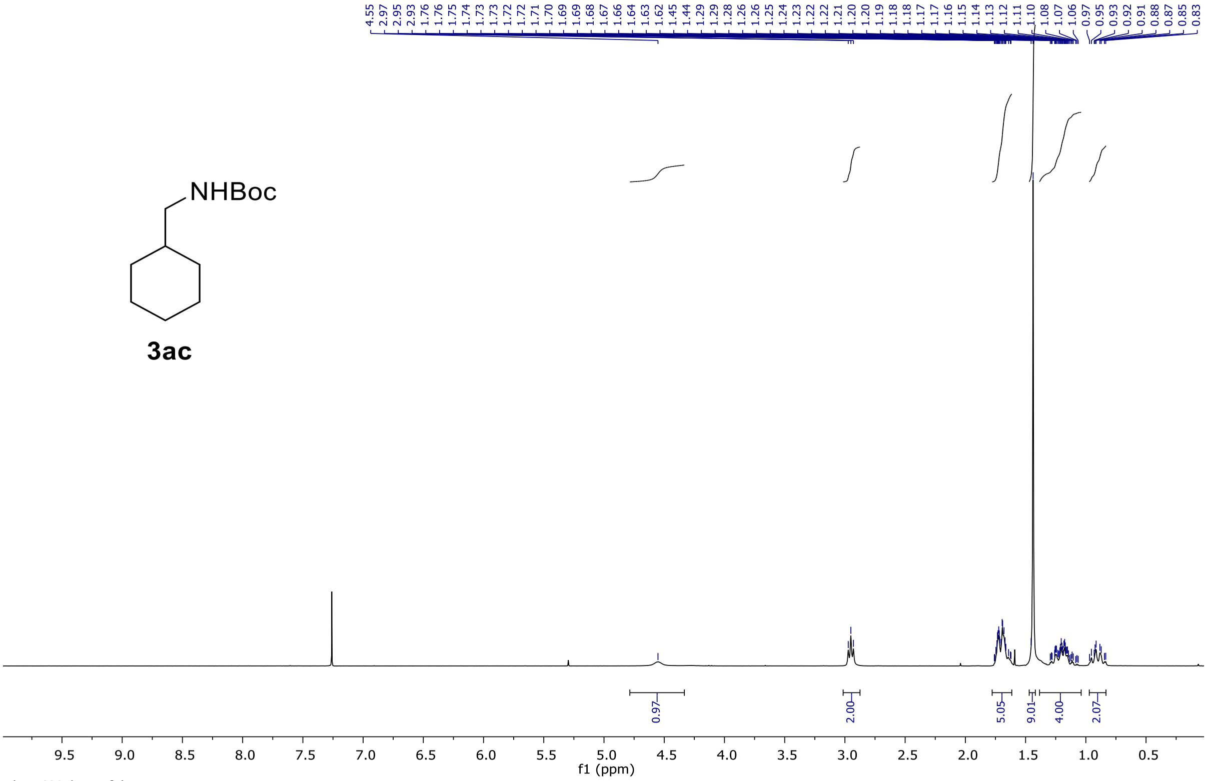

ebmc609char.1.fid
13C 75.5MHz Job 95960 McLean Euan B 609CHAR CDCl3 25.0�C 3 hours 1 min
carbon characterisation

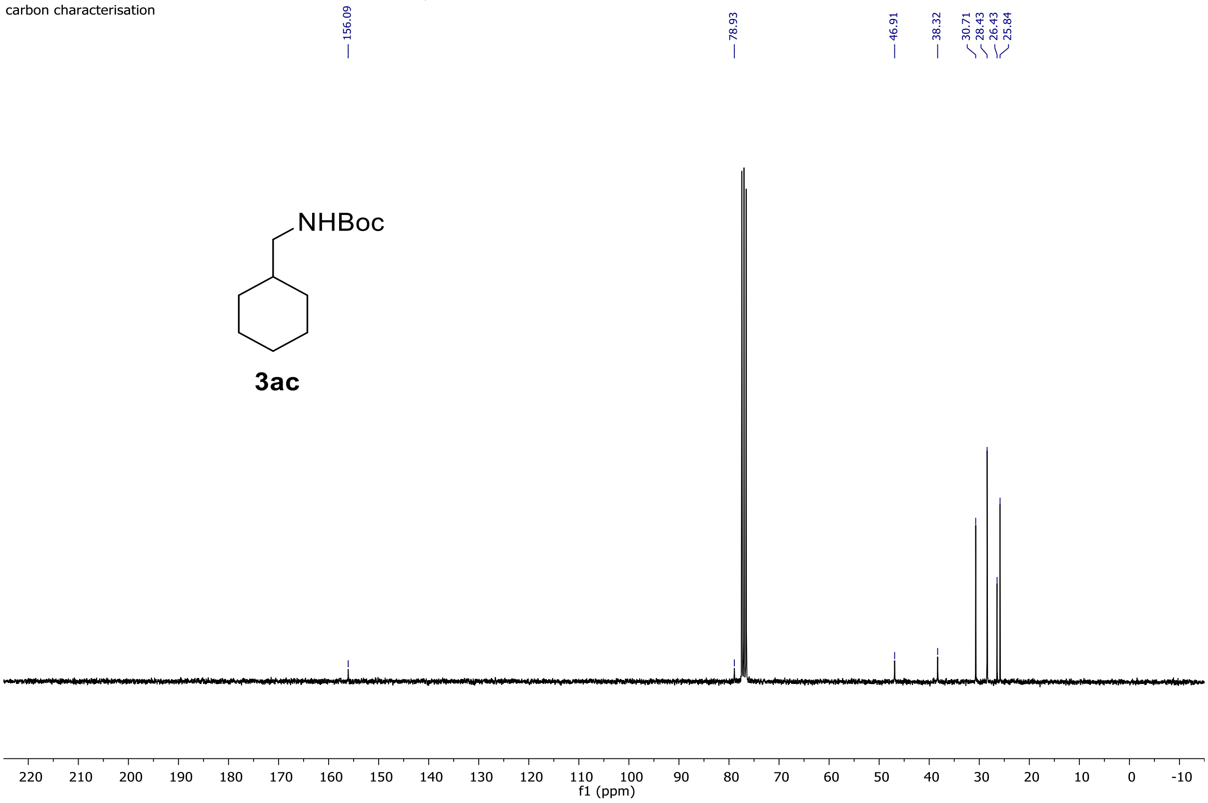

ebmh559char.1.fid
1H 300.1MHz Job 94876 McLean Euan B 559CHAR CDCl3 25.1�C
proton charcaterisation

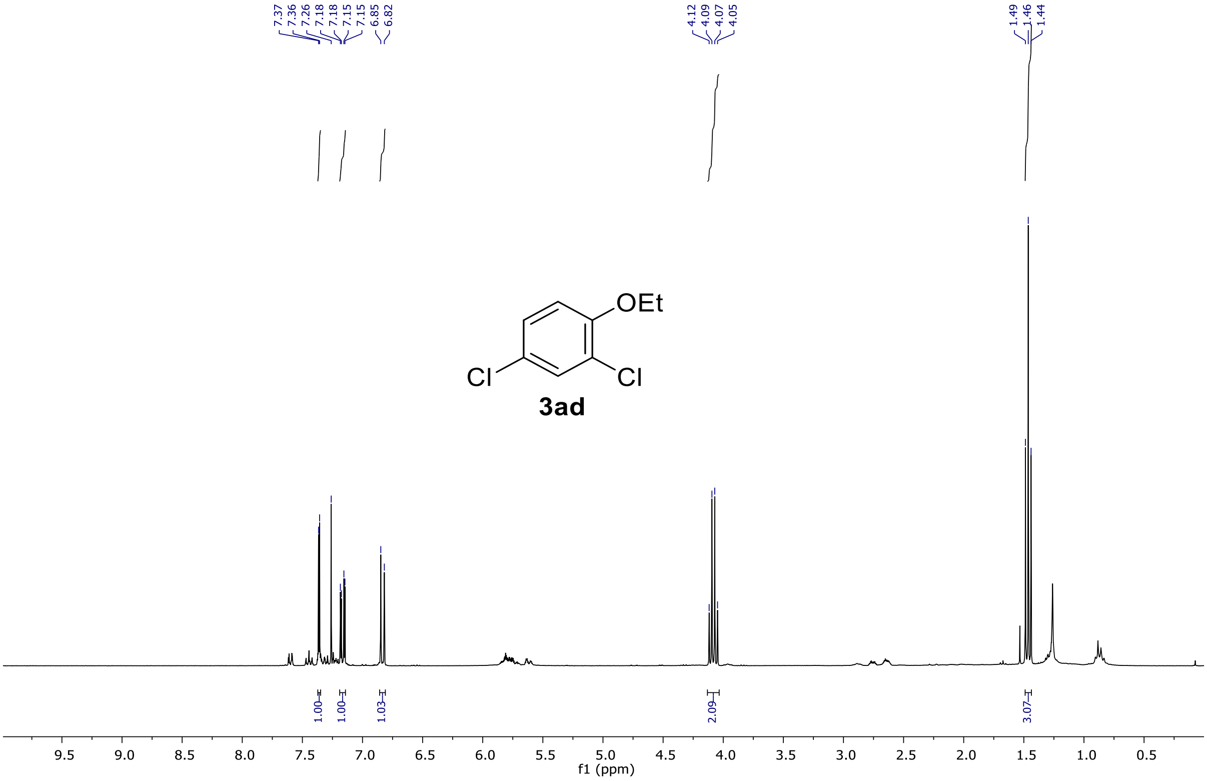

ebmc559char.1.fid
13C 75.5MHz Job 94887 McLean Euan B 559CHAR CDCl3 25.1�C 3 hours 1 min
carbon characterisation

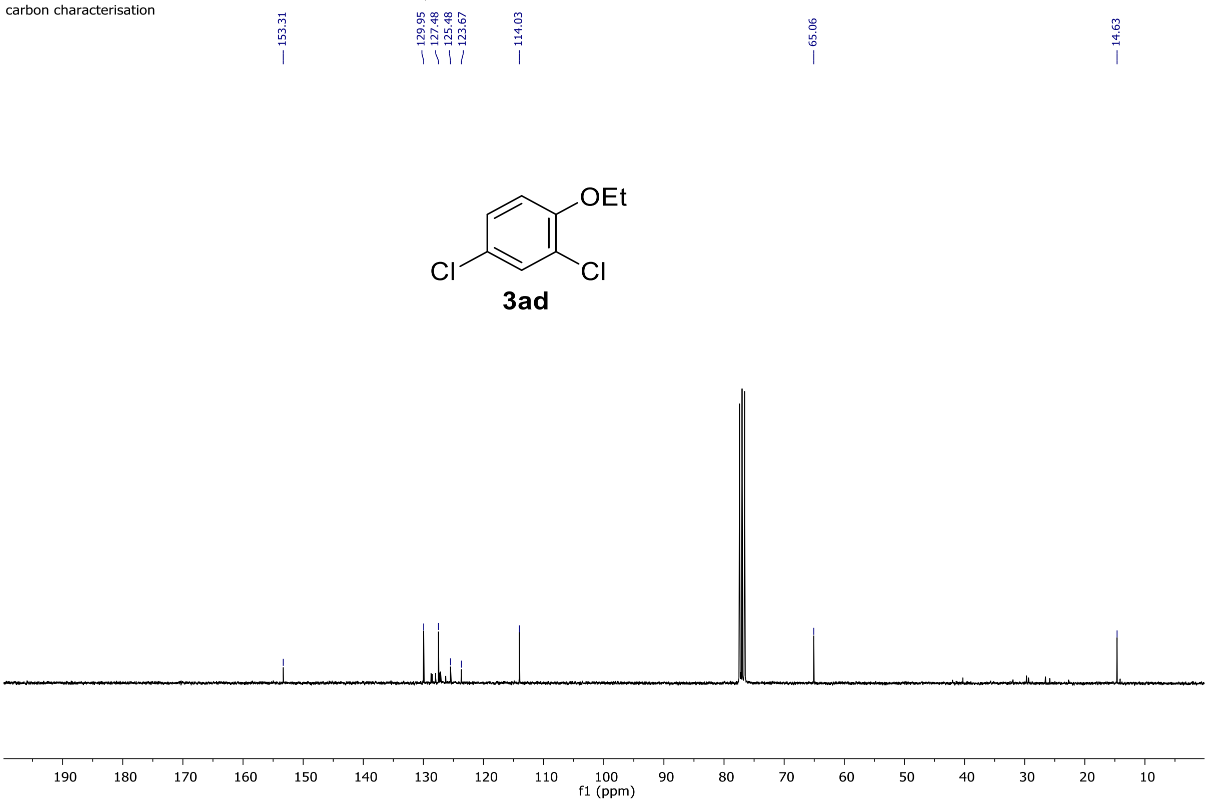

References

1. Sutherland, D. R.; Veguillas, M.; Oates, C. L.; Lee, A.-L., *Org. Lett.* **2018**, *20*, 6863.
2. Westwood, M. T.; Lamb, C. J. C.; Sutherland, D. R.; Lee, A.-L., *Org. Lett.* **2019**, *21*, 7119.
3. Kolthoff, I. M.; Meehan, E. J.; Carr, E. M., *J. Am. Chem. Soc.* **1953**, *75*, 1439.
4. Zil'berman, E. N.; Krasavina, N. B.; Navolokina, R. A.; Kharitonova, O. A., *Zh. Obshch. Khim.* **1986**, *56*, 937.
5. Griffin, J. D.; Cavanaugh, C. L.; Nicewicz, D. A., *Angew. Chem. Int. Ed.* **2017**, *56*, 2097.
6. Johnson, C. R., *J. Chem. Educ.* **1970**, *47*, 702.
7. A. D. Kirk, C. N., *Anal. Chem.* **1983**, *55*, 2428.
8. J. N. Demas, W. D. B., E. F. Zalewski, R. A. Velapoldi, *J. Phys. Chem.* **1981**, *85*, 2766.
9. Prier, C. K.; Rankic, D. A.; MacMillan, D. W. C., *Chem. Rev.* **2013**, *113*, 5322.
10. Dai, C.; Meschini, F.; Narayanam, J. M. R.; Stephenson, C. R. J., *J. Org. Chem.* **2012**, *77*, 4425.
11. Griffin, J. D.; Zeller, M. A.; Nicewicz, D. A., *J. Am. Chem. Soc.* **2015**, *137*, 11340.
12. Wu, X.; Wang, M.; Huan, L.; Wang, D.; Wang, J.; Zhu, C., *Angew. Chem. Int. Ed.* **2018**, *57*, 1640.
13. Ventre, S.; Petronijevic, F. R.; MacMillan, D. W. C., *J. Am. Chem. Soc.* **2015**, *137*, 5654.
14. Yang, P.; Xu, H.; Zhou, J., *Angew. Chem. Int. Ed.* **2014**, *53*, 12210.
15. Flowers, B. J.; Gautreau-Service, R.; Jessop, P. G., *Adv. Synth. Catal.* **2008**, *350*, 2947.
16. Zhang, H.; Jiang, C.; Tan, J.-P.; Hu, H.-L.; Chen, Y.; Ren, X.; Zhang, H.-S.; Wang, T., *ACS Catal.* **2020**, *10*, 5698.
17. Betzi, S.; Combes, S.; Collette, Y.; Hoffer, L.; Morelli, X.; Raux, B.; Roche, P.; Voitovich, I. 2017.
18. Svahn, C. M.; Merenyi, F.; Karlson, L., *J. Med. Chem.* **1986**, *29*, 448.
19. Balaban, A. T., *J. Label. Compd. Radiopharm.* **1981**, *18*, 1621.
20. Borah, A.; Goswami, L.; Neog, K.; Gogoi, P., *J. Org. Chem.* **2015**, *80*, 4722.
21. Yang, M.; Xing, Z.; Fang, B.; Xie, X.; She, X., *Org. Biomol. Chem.* **2020**, *18*, 288.
22. Baek, H.; Kashimura, K.; Fujii, T.; Tsubaki, S.; Wada, Y.; Fujikawa, S.; Sato, T.; Uozumi, Y.; Yamada, Y. M. A., *ACS Catal.* **2020**, *10*, 2148-2156.
23. Zerbi, G.; Magni, R.; Gussoni, M.; Moritz, K. H.; Bigotto, A.; Dirlikov, S., *J. Chem. Phys.* **1981**, *75*, 3175.
24. Qin, T.; Malins, L. R.; Edwards, J. T.; Merchant, R. R.; Novak, A. J. E.; Zhong, J. Z.; Mills, R. B.; Yan, M.; Yuan, C.; Eastgate, M. D.; Baran, P. S., *Angew. Chem. Int. Ed.* **2017**, *56*, 260.
25. Diéguez, H. R.; López, A.; Domingo, V.; Arteaga, J. F.; Dobado, J. A.; Herrador, M. M.; Quílez del Moral, J. F.; Barrero, A. F., *J. Am. Chem. Soc.* **2010**, *132*, 254.
26. Antoniak, D.; Barbasiewicz, M., *Org. Lett.* **2019**, *21*, 9320.
27. Wilden, J. D.; Geldeard, L.; Lee, C. C.; Judd, D. B.; Caddick, S., *Chem. Commun.* **2007**, 1074.
28. Nishikawa, M.; Inaba, Y.; Furukawa, M., *Chem. Pharm. Bull.* **1983**, *31*, 1374.
29. Guin, J.; Fröhlich, R.; Studer, A., *Angew. Chem. Int. Ed.* **2008**, *47*, 779.
30. Sang, R.; Kucmierczyk, P.; Dong, K.; Franke, R.; Neumann, H.; Jackstell, R.; Beller, M., *J. Am. Chem. Soc.* **2018**, *140*, 5217.
31. Liu, D.; Li, Y.; Qi, X.; Liu, C.; Lan, Y.; Lei, A., *Org. Lett.* **2015**, *17*, 998.
32. Barker, G.; O'Brien, P.; Campos, K. R., *Org. Lett.* **2010**, *12*, 4176.
33. Naidu, A. B.; Jaseer, E. A.; Sekar, G., *J. Org. Chem.* **2009**, *74*, 3675.
34. Lam, K.; Markó, I. E., *Org. Lett.* **2011**, *13*, 406.
35. Longwitz, L.; Jopp, S.; Werner, T., *J. Org. Chem.* **2019**, *84*, 7863.
36. Olah, G. A.; Welch, J. T.; Vankar, Y. D.; Nojima, M.; Kerekes, I.; Olah, J. A., *J. Org. Chem.* **1979**, *44*, 3872.
37. Denmark, S. E.; Henke, B. R., *J. Am. Chem. Soc.* **1991**, *113*, 2177.
38. Molle, G.; Bauer, P., *J. Am. Chem. Soc.* **1982**, *104*, 3481.
39. Smith, G. W.; Williams, H. D., *J. Org. Chem.* **1961**, *26*, 2207.

40. Keenan, M.; Abbott, M. J.; Alexander, P. W.; Armstrong, T.; Best, W. M.; Berven, B.; Botero, A.; Chaplin, J. H.; Charman, S. A.; Chatelain, E.; von Geldern, T. W.; Kerfoot, M.; Khong, A.; Nguyen, T.; McManus, J. D.; Morizzi, J.; Ryan, E.; Scandale, I.; Thompson, R. A.; Wang, S. Z.; White, K. L., *J. Med. Chem.* **2012**, *55*, 4189.
41. Bruneau, P.; Delvare, C.; Edwards, M. P.; McMillan, R. M., *J. Med. Chem.* **1991**, *34*, 1028.
42. Guan, B.-T.; Xiang, S.-K.; Wang, B.-Q.; Sun, Z.-P.; Wang, Y.; Zhao, K.-Q.; Shi, Z.-J., *J. Am. Chem. Soc.* **2008**, *130*, 3268.
43. Pérez-Prieto, J.; Galian, R. E.; Morant-Miñana, M. C., *ChemPhysChem* **2006**, *7*, 2077.
44. Ho, J.; Zheng, J.; Meana-Pañeda, R.; Truhlar, D. G.; Ko, E. J.; Savage, G. P.; Williams, C. M.; Coote, M. L.; Tsanaktsidis, J., *J. Org. Chem.* **2013**, *78*, 6677.
45. Patrick, T. B.; Khazaeli, S.; Nadji, S.; Hering-Smith, K.; Reif, D., *J. Org. Chem.* **1993**, *58*, 705.
46. De, S.; Yin, J.; Ma, D., *Org. Lett.* **2017**, *19*, 4864.
47. Wang, C.; Liu, L.; Wang, W.; Ma, D.-S.; Zhang, H., *Molecules* **2010**, *15*, 1154.
48. Miyamura, H.; Suzuki, A.; Yasukawa, T.; Kobayashi, S., *J. Am. Chem. Soc.* **2018**, *140*, 11325.
49. Sheridan, T.; Yayla, H. G.; Lian, Y.; Genovino, J.; Monck, N.; Burton, J. W., *Eur. J. Org. Chem.* **2020**, *2020*, 2766.
